# Single-cell transcriptomics reveals EpCAM regulates the development and morphology of intestinal epithelium via controlling the EGFR pathway

**DOI:** 10.1016/j.gendis.2026.102072

**Published:** 2026-02-09

**Authors:** Zili Lei, Ya Nie, Lulu Liu, Yanhong Yang, Xuan Liu, Guibin Chen, Yunjuan Wang, Wanwan Liu, Qing Hu, Ting Lin, Jiao Guo

**Affiliations:** a Guangdong Metabolic Diseases Research Center of Integrated Chinese and Western Medicine, Guangdong Pharmaceutical University, Guangzhou, Guangdong 510006, China; b School of Traditional Chinese Medicine, Guangdong Pharmaceutical University, Guangzhou, Guangdong 510006, China; c The First Affiliated Hospital (The First Clinical Medical School), Guangdong Pharmaceutical University, Guangzhou, Guangdong 510080, China

Congenital tufting enteropathy (CTE), which is a rare inherited intractable diarrhea of infancy and is characterized by intestinal epithelial cell (IEC) dysplasia and villus tufting, is mostly caused by the loss-of-function mutation of EpCAM.^1^ However, the mechanisms of EpCAM on regulating the development and morphology of the intestinal epithelium remain unclear. The expression level of EpCAM is higher in the crypts than the villi of small intestines,^1^ indicating that it plays important roles in the intestinal stem cells (ISCs). We recently found that EpCAM maintains the longevity of ISCs.^2^ However, the functions of EpCAM in the differentiation and division of ISCs still need to be explored. Here, we performed single-cell RNA sequencing (scRNA-seq) to compare the developmental potential of IECs from wild type (WT), EpCAM^+/−^ and EpCAM^−/−^ embryos at the E18.5 stage and uncovered the pathological mechanism of CTE that EpCAM deficiency might elevate the activation of EGFR in the ISCs and transit-amplifying (TA) cells to cause the tufting intestinal epithelium and the immaturity of IECs.

The phenotype of EpCAM^−/−^ mice was similar to that in previous reports^1^^,^^2^ (Fig. S1). According to reported methods,^3^ IECs from WT, EpCAM^+/−^ and EpCAM^−/−^ embryos were separated into 15 clusters via scRNA-seq (Fig. 1A; Fig. S2A and S3). Depending on reported marker genes,^4^ these IECs were considered as enterocytes (Clusters 0, 2, 6 and 13), pre-mature enterocytes (Clusters 1, 4, 5, 7 and 12), ISCs or TA cells (Cluster 3), TA cells with a later developmental stage (Cluster 11), goblet or Paneth cells (Cluster 8), pre-mature goblet cells or Paneth cells (Clusters 9 and 14), and enteroendocrine cells (Cluster 10) (Fig. 1B; Fig. S2B). The cell numbers from most clusters related to enterocytes were lower in the mutant (Fig. S2C and S2D), and marker genes of enterocytes, including *Alpi*, *Fabp1*, *Fabp2*, *Rbp2*, *Aqp11*, *Slc17a5* and *Slc2a2*, were significantly down-regulated in the small intestines of EpCAM^−/−^ E18.5 embryos (Fig. 1C; Fig. S4A, S4B, S5, S6 and Table S1). Importantly, *Hnf4a* and *Hnf4g*, encoding the transcription factors HNF4A and HNF4G, which control the differentiation towards enterocytes,^5^ were significantly reduced in the mutant (Fig. 1D; Fig. S4C, S4G, S7 and Table S1). The cell numbers of clusters related to goblet or Paneth cells were higher in the mutant (Fig. S2C and S2D), and marker genes of goblet or Paneth cells, including *Muc2*, *Clca1*, *Tff3*, *Agr2*, *Lyz1*, *Defa17*, *Defa24* and *Ang4*, were all significantly up-regulated in the mutant intestines (Fig. 1C and D; Fig. S4D, S4E, S8 and Table S1). Furthermore, *Sox9*, *Spdef*, *Klf4* and *Gfi1*, which encode transcription factors for the differentiation of goblet cells or Paneth cells,^5^ were increased in the mutant (Fig. S4F, S4G, S9 and Table S1). However, the size of most Periodic Acid-Schiff positive cells was smaller in the intestines of EpCAM^−/−^ mice than in those of the WT mice, although the number of these cells was increased in the mutant (Fig. S10), indicating the immaturity of goblet cells in the mutant intestines.

**Figure 1:**
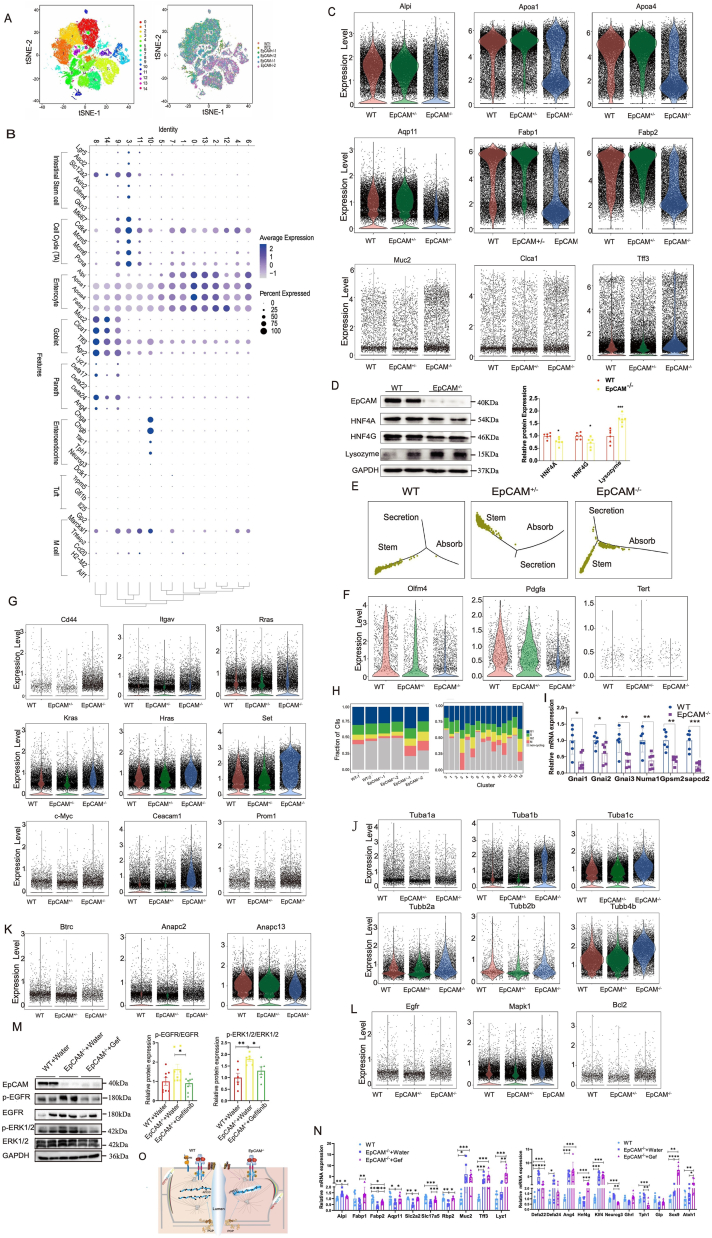
Functional analysis of EpCAM on regulating the development and morphology of the intestinal epithelium. **(A)** The tSNE plot showed 48,733 IECs separated into 15 clusters (left) and the distribution of IECs from 2 wild type (WT), 2 EpCAM^+/−^, and 2 EpCAM^−/−^ E18.5 embryos of littermate in each cluster (right). **(B)** Dot plot showed the average expression levels of well-defined IEC marker genes. **(C)** Violin plots compared the expression levels of *Alpi*, *Apoa1*, *Apoa4*, *Aqp11*, *Fabp1*, *Fabp2*, *Muc2*, *Clca1* and *Tff3* in the IECs from WT (Red), EpCAM^+/−^ (Green) and EpCAM^−/−^ (Blue) E18.5 embryos. **(D)** Western blot analysis of the levels of EpCAM, HNF4A, HNF4G and Lysozyme in the small intestines from the WT and EpCAM^−/−^ groups of embryos at the E18.5 stage. Right panels: quantification data. Six mice in each group were used for 3 independent experiments. **(E)** Developmental trajectories of IECs in Cluster 3 from WT, EpCAM^+/−^ and EpCAM^−/−^ E18.5 embryos respectively. **(F)** Violin plots compared the mRNA levels of *Olfm4*, *Pdgfa*, and *Tert* in the IECs from Cluster 3 of WT (Red), EpCAM^+/−^ (Green) and EpCAM^−/−^ (Blue) E18.5 embryos. **(G)** Violin plots compared the expression levels of *Cd44*, *Itgav*, *Rras*, *Kras*, *Hras*, *Set*, *c-Myc*, *Ceacam1* and *Prom1* in IECs from WT (Red), EpCAM^+/−^ (Green) and EpCAM^−/−^ (Blue) E18.5 embryos. **(H)** The ratios of IECs at each phase of the cell cycle from every sample and cluster. **(I)** The qPCR results of *Gnai1*, *Gnai2*, *Gnai3*, *Numa1*, *Gpsm2* and *Sapcd2* from the small intestines of the WT and EpCAM^−/−^ groups. **(J)** Violin plots compared the expression levels of *Tuba1a*, *Tuba1b*, *Tuba1c*, *Tubb2a*, *Tubb2b* and *Tubb4b* in IECs from WT (Red), EpCAM^+/−^ (Green) and EpCAM^−/−^ (Blue) E18.5 embryos. **(K)** Violin plots compared the expression levels of *Btrc*, *Anapc2* and *Anapc13* in IECs from WT (Red), EpCAM^+/−^ (Green) and EpCAM^−/−^ (Blue) E18.5 embryos. **(L)** Violin plots compared the expression levels of *Egfr*, *Mapk1* and *Bcl2* in IECs from WT (Red), EpCAM^+/−^ (Green) and EpCAM^−/−^ (Blue) E18.5 embryos. **(M)** Western blot analysis of the levels of EpCAM, p-EGFR, EGFR, p-ERK1/2 and ERK1/2 in the small intestines from the WT + Water, EpCAM^−/−^ + Water and EpCAM^−/−^ + Gef groups of embryos at the E18.5 stage. Right panels: quantification data. Six mice in each group were used for 3 independent experiments. **(N)** The relative mRNA expression of *Alpi*, *Fabp1*, *Fabp2*, *Aqp11*, *Slc2a2*, *Slc17a5*, *Rbp2*, *Muc2*, *Tff3*, *Lyz1*, *Defa22*, *Defa24*, *Ang4*, *Hnf4g*, *Klf4*, *Neurog3*, *Ghrl*, *Tph1*, *Gip*, *Sox9* and *Atoh1* in the small intestines from the WT + Water, EpCAM^−/−^ + Water and EpCAM^−/−^ + Gef groups. **(O)** The deficiency of EpCAM causes the hyper-activation of the EGFR signaling pathway in intestinal stem cells and transit-amplifying cells, then the hyper-activated EGFR signal induces the decrease of APC/C E3 ubiquitin ligases to arrest these proliferating cells at the M phase of the cell cycle, and finally the incomplete division causes the dysplasia of intestinal epithelial cells. On the other hand, the hyper-activated EGFR signaling interferes with the orientation and position of the mitotic spindle to induce the villus tufting in the intestines of EpCAM deficient mice via affecting the Gαi/LGN/NuMA complex and astral microtubules. ∗*p* < 0.05, ∗∗*p* < 0.01, ∗∗∗*p* < 0.001; IEC, intestinal epithelial cells; Gef, Gefitinib; APC/C, anaphase-promoting complex/cyclosome.

Pseudo-temporal ordering demonstrated the exacerbation of the differentiation of ISCs and TA cells from Cluster 3 of the mutant (Fig. 1E; Fig. S11A, S12, S13). The decrease of *Olfm4*, *Sox9*, *Pdgfa*, *Tert* and *Smoc2* in Cluster 3 of EpCAM^−/−^ mice indicated the exhaustion of their ISCs (Fig. 1F; Fig. S11B, S11D, S14A, S14B and Table S1). However, genes related to tumor formation, such as *Cd44*, *Rras*, *Kras*, *c-Myc* and *Ceacam1*, were significantly up-regulated in the small intestines of EpCAM^−/−^ mice (Fig. 1G; Fig. S11E, S14C–S14E, S17 and Table S1).

The percentage of IECs at G1, G2 and M phases was significantly higher, but the percentage of non-cycling IECs was considerably lower in EpCAM^−/−^ mice (Fig. 1H; Fig. S15A, S15E, S15F, S16). Most genes and markers related to cell proliferation were significantly increased in the mutant intestines (Fig. S15B–S15D, S17 and Table S1). Moreover, KEGG analysis results showed that abilities of DNA replication, base excision repair, nucleotide excision repair and homologous recombination were significantly reduced in Cluster 3 of the mutant (Fig. S15G).

The transcription of genes related to apical–basal polarity and planar cell polarity, such as *Pard3b*, *Pard6b*, *Prkci*, *Prkcz*, *Fzd2*, *Fzd4*, *Fzd8*, *Dvl1* and *Dvl2*, was significantly affected in the mutant IECs or some clusters of them (Fig. S18A–S18F, S19A–S19D, S20, S21 and Table S1). Genes related to the Gαi/LGN/NuMA complex, including *Numa1*, *Gpsm2* and *Sapcd2*, were all significantly down-regulated in the small intestines of EpCAM^−/−^ mice (Fig. 1I; Fig. S18G, S18H, S19E, S19F, S22 and Table S1). Genes encoding components of astral microtubules, such as *Tuba1b*, *Tuba1c*, *Tuba4a*, *Tubb2a*, *Tubb2b*, *Tubb4b*, *Tubb5* and *Tubg1*, were significantly increased in IECs of EpCAM^−/−^ mice (Fig. 1J; Fig. S18I, S18J, S19G, S23 and Table S1).

Several E3 ubiquitin ligases that play important roles in the WNT pathway or cell cycle, including β-TrCP, anaphase-promoting complex/cyclosome (APC/C) and Skp1-Cul1-Fbox were affected because genes encoding components of them, such as *Btrc*, *Anapc2*, *Anapc11*, *Anapc5*, *Anapc13* and *Fzr1*, were significantly down-regulated in the IECs or some clusters of EpCAM^−/−^ mice (Fig. 1K; Fig. S24–S27 and Table S1). However, most targeting genes of these E3 ubiquitin ligases and the WNT pathway were increased in the mutant IECs (Fig. S24–S30 and Table S1).

The up-regulation of *Egfr*, *Mapk1*, *Rps6*, *Eif4e*, *Eif4ebp1*, *Bcl2* and *Bcl2l1* in the mutant IECs (Fig. 1L; Fig. S31A–S31C, S32 and Table S1) and the increase of EGFR, p-EGFR and p-ERK1/2 in the mutant small intestines (Fig. 1M; Fig. S33) demonstrated the hyper-activation of the EGFR pathway in the mutant intestinal epithelium. After a decrease in the activity of EGFR with gefitinib (Fig. 1M; Fig. S31D), the transcriptions of *Alpi*, *Fabp1*, *Fabp2*, *Aqp11*, *Slc2a2*, *Slc17a5*, *Rbp2* and *Hnf4g* were all significantly rescued in the small intestines of EpCAM^−/−^ mice (Fig. 1N). Gefitinib also improved the exhaustion of ISCs and the immaturity of goblet cells and Paneth cells, up-regulated genes encoding components of the Gαi/LGN/NuMA complex and E3 ubiquitin ligases, and down-regulated genes related to tumor formation (Fig. 1N; Fig. S31E–S31H). These results demonstrated that the hyper-activation of EGFR signaling might be the mechanism on the IEC dysplasia and the villus tufting of EpCAM^−/−^ mice.

In summary, EpCAM deficiency induces the hyper-activation of EGFR in the ISCs and TA cells, which induces the decrease of APC/C E3 ubiquitin ligases to arrest these cells at the M phase of the cell cycle and interferes with the orientation and position of mitotic spindles of them, then finally causing dysplasia of IECs and villus tufting (Fig. 1O).

## CRediT authorship contribution statement

**Zili Lei:** Writing – review & editing, Writing – original draft, Visualization, Validation, Supervision, Software, Resources, Project administration, Methodology, Investigation, Funding acquisition, Formal analysis, Data curation, Conceptualization. **Ya Nie:** Visualization, Validation, Supervision, Resources, Project administration, Methodology, Investigation, Data curation, Conceptualization. **Lulu Liu:** Visualization, Validation, Methodology, Formal analysis, Data curation. **Yanhong Yang:** Writing – review & editing, Writing – original draft, Visualization, Resources, Project administration, Methodology, Investigation, Funding acquisition, Conceptualization. **Xuan Liu:** Data curation, Formal analysis, Project administration, Supervision. **Guibin Chen:** Validation, Methodology, Data curation, Conceptualization. **Yunjuan Wang:** Visualization, Validation, Resources, Methodology, Investigation. **Wanwan Liu:** Software, Resources, Methodology, Investigation, Funding acquisition. **Qing Hu:** Software, Resources, Project administration, Funding acquisition, Formal analysis. **Ting Lin:** Project administration, Methodology, Investigation, Formal analysis. **Jiao Guo:** Funding acquisition.

## Ethics declaration

All animal experimental procedures were approved by the Experimental Animal Ethics Committee of Guangdong Pharmaceutical University.

## Data availability

The scRNA-seq data have been deposited at SRA and the accession number is PRJNA1127855.

## Funding

This work was supported by the 10.13039/501100001809National Natural Science Foundation of China (No. 82171855, 81830113); the 10.13039/501100021171Guangdong Basic and Applied Basic Research Foundation (No. 2021A1515012383); and the Key Field Special Project for Colleges and Universities of Guangdong Province (Biomedicine and Health) (No. 2023ZDZX2030).

## Conflict of interests

The authors have declared that no conflict of interests exists.
