# Supplementary material for: Single-cell transcriptomics reveals EpCAM regulates the development and morphology of intestinal epithelium via controlling the EGFR pathway
Source: Genes Dis. 2026 Feb 9;13(5):102072. doi: 10.1016/j.gendis.2026.102072 (PMC13157056; doi:10.1016/j.gendis.2026.102072)
Supplement: Multimedia component 2 [file mmc2.doc]

**Index**

**• Supplemental tables description**

**• Methods**

**• References to Supplemental Material**

**Supplemental tables description**

**Table S2. Sequences of primers for qPCR experiments**

| OligomerName | Forward primer | Reverse primer |
| --- | --- | --- |
| EpCAM | CCTGAGAGTGAACGGAGAGC | CACAATGACAGCGATGATCC |
| Pdgfb | AATGCTGAGCGACCACTCCATC | TCGGGTCATGTTCAAGTCCAGC |
| Gas6 | GAACTTGCCAGGCTCCTACTCT | GGAGTTGACACAGGTCTGCTCA |
| MAPK1 | TCAAGCCTTCCAACCTCCTGCT | AGCTCTGTACCAACGTGTGGCT |
| Rps6 | TGTCCGCCAGTATGTTGTCAGG | CACGAGGAGTAACAAGTCGCTG |
| Sos1 | CAACATACAGGTCCTTTTGCAGAC | TGCGATCAGCTTCTGTTGGCTC |
| Eif4ebp1 | GGAGAGCTGCACAGCATTCAGG | GGAGGTATGTGCTGGTGTTCAC |
| Pdgfa | CTGGCTCGAAGTCAGATCCACA | GACTTGTCTCCAAGGCATCCTC |
| Kras | GGAGTACAGTGCAATGAGGGAC | CCAGGACCATAGGCACATCTTC |
| Axl | GGTGTTTGAGCCAACCGTGGAA | GCCACCTTATGCCGATCTACCA |
| Hras | TCGCACTGTTGAGTCTCGGCAG | TATGCTGCCGAATCTCACGGAC |
| Eif4e | ATGCCTGGCTGTGACTACTCAC | GTGTCTCTAGCCAGAAGCGATC |
| Bcl2l1 | GCCACCTATCTGAATGACCACC | AGGAACCAGCGGTTGAAGCGC |
| Bcl2 | CCTGTGGATGACTGAGTACCTG | AGCCAGGAGAAATCAAACAGAGG |
| Egfr | GGACTGTGTCTCCTGCCAGAAT | GGCAGACATTCTGGATGGCACT |
| Alpi | CCAGCAGTAACTCACCTCATGG | GAAGCCTTGTGGATTCCTGCTG |
| Apoa4 | CAGAAGACGGATGTCACTCAGC | AGCTGTACGACAAAGGGCACCA |
| Fabp2 | TCCCTACAGTCTAGCAGACGGA | CCAGAAACCTCTCGGACAGCAA |
| Rbp2 | CTACGACCTGGATTTCACCGTC | ACTGCTTCCAGCCACGGTTCTC |
| Lyz1 | TACAACCGTGGAGACCGAAGCA | TGGCTGCAGTGATGTCATCCTG |
| Defa17 | GACCAGGCTGTGTCTGTCTC | ACCAGATCTCTCAACGATTCCT |
| Defa22 | CTAATACTGAGGAGCAGCCAGG | GCAGCGACGTTTTCTACAAAGGC |
| Defa24 | CTAAAACTGAGGAGCAGCCAGG | CTTCCTTTGCAGCCTCTTGCTC |
| Ang4 | GGCACCAAGAAAAACATCAGGGC | GTGCGTACAAGTGGTGATCTGG |
| Lgr5 | AGAGCCTGATACCATCTGCAAAC | TGAAGGTCGTCCACACTGTTGC |
| Ascl2 | TTTCCTGTGCCGCACCAGAACT | CAGCGACTCCAGACGAGGTGG |
| Slc12a2 | GATTCGCAGAGACTGTGGTGGA | CTCCATTCCAGCCACTGAGATG |
| Axin2 | ATGGAGTCCCTCCTTACCGCAT | GTTCCACAGGCGTCATCTCCTT |
| Olfm4 | GCCTCCAAAAGTGACCTTGTGC | TGCGTGTGCTGGTGGAAAAGAG |
| Gkn3 | GCCTGTGTTCTGGCAAAGATGG | GGCTGGGTAAAACTGTGTAGGTC |
| Smoc2 | GCCAAGTGCAAAGATCCACAGC | ACACTTGCTGGAACTCCTTCCG |
| Tnfrsf19 | TGTGTCCTCTGCAAACAGTGCG | CCAGTCTTCCTTGAACCGTTGC |
| Rnf43 | CTGGCTATACCAGCATCGGACT | ATGCTGGCGAATGAGGTGGAGT |
| Msi1 | GTTCATCGGAGGACTCAGTTGG | CTGGTCCATGAAAGTGACGAAGC |
| Prom1 | CTGCGATAGCATCAGACCAAGC | CTTTTGACGAGGCTCTCCAGATC |
| Sox9 | CACACGTCAAGCGACCCATGAA | TCTTCTCGCTCTCGTTCAGCAG |
| BMPr1a | ATCCGATGGCTGGTTGTGCTCA | CCAAATCACGGTTGTAACGACCC |
| Ppm1d- | TCACAGTGGACCTGTCAGAAGG | AGAGTGTGGACACTGGTGTCTG |
| Sox4 | GATCTCCAAGCGGCTAGGCAAA | GTAGTCAGCCATGTGCTTGAGG |
| Sfrp5 | GAGATGCTGCACTGCCACAAGT | TGCTCCATCTCACACTGGGCAC |
| Dclk1 | TACCGACGCTATCAAGCTGGAC | GGTAACGGAACTTCTCTGGTCC |
| Bmi1 | ACTACACGCTAATGGACATTGCC | CTCTCCAGCATTCGTCAGTCCA |
| Chga | AGAACCAGAGCCCTGATGCCAA | CTCTGTGGTTGCCTCAAAGCCA |
| Chgb | TCTACGACTCGGAGGAGCAGAT | CCGCTATCTTCTGGAGTTCCAG |
| Tac1 | TAATGGGCAAGCGGGATGCTGA | CCATTAGTCCAACAAAGGAATCTG |
| Tph1 | TGTTGACTGCGACATCAGCCGA | GGAAACCAAGGGACAGTCTCCA |
| Neurog3 | TCCGAAGCAGAAGTGGGTGACT | CGGCTTCTTCGCTGTTTGCTGA |
| Sct | CTCAGCCGCTTGCAGGACAGT | CTGAGTGTTCGACCACAGCAAG |
| Gcg | CCTTCAAGACACAGAGGAGAACC | CTGTAGTCGCTGGTGAATGTGC |
| Cck | TAGCGCGATACATCCAGCAGGT | GGTATTCGTAGTCCTCGGCACT |
| Ghrl | GAAGCCACCAGCTAAACTGCAG | CTGACAGCTTGATGCCAACATCG |
| Nts | AGAAGATGTGAGAGCCCTGGAG | TGAGGCTGCAAACATTTAGCAAGG |
| Pyy | GCCACTACCTCAACCTGGTCAC | TCGCTGTCGTCTGTGAAGAGCA |
| Gip | CAAGACTTCGTGAACTGGCTGC | GTCCTCCTTTCCCTGAGATTGC |
| Gast | GAACAGCGCCAGTTCAACAAGC | GCCAAAGTCCATCCATCCGTAG |
| Sst | CCAGACTCCGTCAGTTTCTGCA | GGCATCATTCTCTGTCTGGTTGG |
| Mki67 | GAGGAGAAACGCCAACCAAGAG | TTTGTCCTCGGTGGCGTTATCC |
| Cdk4 | CATACCTGGACAAAGCACCTCC | GAATGTTCTCTGGCTTCAGGTCC |
| Mcm5 | GGAGGCATTGAGACTGTTCCAG | AGACACCTGAGAGCCAATGGCA |
| Mcm6 | CGACAGCTTGAGAGCATGATCC | TGACATCAGGCGTCTCTACACG |
| Pcna | CAAGTGGAGAGCTTGGCAATGG | GCAAACGTTAGGTGAACAGGCTC |
| Cd44 | CGGAACCACAGCCTCCTTTCAA | TGCCATCCGTTCTGAAACCACG |
| Rras | GGCAAGCTCTTCACACAGATCC | GGAAGCACTGAAAGAGGAGGCT |
| Met | GTTCTGCTTGGCAACGAGAGCT | GGAGAATGCACTGTATTGCGTCG |
| c-Myc | TGCCAGGAGCTACTGCGCTGT | CATGAAGGGTCATAGCTGCGGT |
| Set | TGACCCGTCTTCAAAGTCCACC | AGCACCTGCGTCAGAATGGTCA |
| Ceacam1 | CCTCTATTCCAGGAAGTCTGGC | GTTCAGGACAGTGTATGCGACG |
| Dnah2 | CCTCAACACCAACGAATGGACC | TCATGCTCTCGATCCACAGCGT |
| Scrib | ACTGCCTTGGAAGGACCATACC | CCAGGATCTTGGACACCAAACG |
| Marveld3 | GATTGCCACAAATGCAGATACTTG | AGTTATTTCCGAAGCCGCTGCC |
| Marveld2 | CCACATTCCGAAGCCTATCGTG | TGCACTTCCGCAGACAGCTCTT |
| Gnai3 | GACTACAGGCATTGTGGAGACC | GGTCGTAATCACTGAGAGCCAC |
| Crb3 | CGGACCCTTTCACAAATAGCACA | TCGCATGAGCAGAAACAGTCCC |
| Crb2 | GGCTACAGAAAGTGGTGGCT | TCACAGGTCACGCCTGCATA |
| Crb1 | CCTGTCAAAACAGAGGACGCTG | CCGCATATCCTGTGGAGTCATC |
| Patj | GGAAGATTTGCCTCTGTACCGAC | GCTGAAGTTCGGTGTCTCCTCT |
| Pals1 | GCGTTGTTAGCCAAGGAAGGCA | CGAAGTAGTGTCCGTTGTTCTGC |
| Prkcz | GGCAGAGAAAACTTCCAGAGGAG | ATGTGTCCGTCGGCATCAAGGA |
| Prkci | GTTTGAGCAGGCGTCCAATCAC | CAGGAAGTTTTCTCTGTCGCTGC |
| Pard6g | ACCATCGGCTATGCTGATGTGC | TCTTCCGTGACAGAGTGCCTGC |
| Pard6b | TTTCCACCGCCAATCCACTGCT | GCTGATGACGATGTGAGGCTTC |
| Pard6a | TACGGATGCTCACGGTGACTTG | GAGAGTTGGAGGCAAAAGCCAG |
| Pard3b | CCAGTTGGTGATTCTGAGGAGAC | ATTTCGCCTGGAGGTGCTCAGA |
| Pard3 | GAGACTCTACGGAGGTCCATGT | TCGGTCATCCAGTTCTGTCTCG |
| Rab11a | CAAGAAGCATCCAGGTTGATGGG | AAGGCACCTACTGCTCCACGAT |
| Atn1 | TTCCTCACCCTCTGCACGAGAA | CCGCTGACATTGGAGCAGAAAG |
| Dvl1 | ATCACACGCACCAGCTCTTCCT | AGTCCTGAGTCTGGCAACTGCA |
| Dvl2 | CCTCATCCTTCAGCAGTGTCAC | CCACAATGGAGATGCCCAGGAA |
| Dvl3 | TGATGGACGCATTGAGCCAGGA | ACAATCTCCCGAAGGACTCGGA |
| Fat1 | GGCTCAGTTGTGCTGCAAGTGA | CTTCGGTCCAATTCTCTGGCAG |
| Fat2 | AGAATCACGGCGTCAGATGGCA | TCACGGACCTTGCCAGTGTAGA |
| Fat3 | GCTGTTCTCAGCACCTTGGACA | GCCTCTTCACATAGACCTTCCAC |
| Fat4 | TGTCCTGTTGGTGAATGCCTCG | GGTTTCTCTGTCCAGCAAAGCAC |
| Fjx1 | GGCGTTGGTCTTTCTGGACAATG | CGACTGTAGCAGCGGTTCGTTA |
| Lrp5 | CCTCACCATTGATTATGCCGACC | GATCGTCAGCTATCACCATGCG |
| Lrp6 | CTGAATGCTGACAACAGGACCTG | GACGTTCCGAAGGCTGTGGATA |
| Prickle1 | AACAGCTCCTGTACCAGTTGCC | CTTCCTCTGAGCACTGAACACC |
| Prickle2 | ATGACACCCACGGAACAGACTC | GGAAAACCGTGATAGGTGCTCC |
| Prickle3 | CACCGCTCACAACTTCTACAGC | CAGTTCAGGCATAGAATGGCGG |
| Prickle4 | TCGATGTCCAGCTTGTGACC | CGGGTCCTTCTTCCTCAAAAGA |
| Vangl1 | GAACACGAACGCAGGGTGAAGA | CCATCACCTCTCCAGGAGACTT |
| Vangl2 | GCATCCTTCAGCACCTGGAGTT | TCGCTCACCAAGGTCCACTGTT |
| Fzd1 | GTGCTCACGTACCTAGTGGACA | TCCTCCAACAGAAAGCCAGCGA |
| Fzd2 | CGCTTCCACTTTCTTCACGGTC | GGAGAACGAAGCCCGCAATGTA |
| Fzd3 | GCAAAGTGAGCAGCTACCATGG | AGCCGATGAGAACTACTGTGCC |
| Fzd4 | ACTTTCACGCCGCTCATCCAGT | TCTCAGGACTGGTTCACAGCGT |
| Fzd5 | CCAGTGTCAAGTCCATTACGGC | CCAAGACAAAGCCTCGTAGTGAG |
| Fzd6 | GTATCTCTGCGGTCTTCTGGGT | CTCACAGGACTCTTGCAGCACT |
| Fzd7 | GATGGTGACCTACTCAGTGGAG | ATGGTGCGGATGCGAAAGAGAG |
| Fzd8 | ACCAGAGCCTTGACAACCTACG | GCTTGATGACTGAACGGATTCGG |
| Fzd9 | TCCAGTACGTGGAGAAGAGTCG | CGTGGAGAAGAAACACAACGCG |
| Fzd10 | CTGGCTTGCTACCTAGTCATCG | TGCGTACCATGAGCTTCTCCAG |
| Gpc4 | GAGGATGACTGCTGGAATGGCA | GACGAAGGATCAGTATGTCTGGC |
| Rack1 | TCCTCTGATGGTCAGTTTGCCC | CACGCTCAACACATCCTTGGTG |
| Odf2 | CCGAGAAGAGTGAGGAGTATGC | CACAACCTGGTTGTAACGGCTC |
| Wnt5a | GGAACGAATCCACGCTAAGGGT | AGCACGTCTTGAGGCTACAGGA |
| Wnt5b | GCTACCGCTTTGCCAAGGAGTT | CATTTGCAGGCGACATCAGCCA |
| Wnt11 | GCCTGTGAAGGACTCAGAACTTG | AGCTGTCACTGCCGTTGGAAGT |
| Atoh1 | CTGGTAAGGAGAAGCGGCTGTG | CCATTCACCTGTTTGCTGGAAGG |
| BMP4 | GCCGAGCCAACACTGTGAGGA | GATGCTGCTGAGGTTGAAGAGG |
| Ccn1 | GTGAAGTGCGTCCTTGTGGACA | CTTGACACTGGAGCATCCTGCA |
| Cdkn1a | TCGCTGTCTTGCACTCTGGTGT | CCAATCTGCGCTTGGAGTGATAG |
| Edn1 | CTACTTCTGCCACCTGGACATC | CGCACTGACATCTAACTGCCTG |
| Efnb2 | CCAACAAGACGTCCAGAGCTAG | CCACTTCGGAACCCAGGAGATT |
| Fosl1 | CCGAAGAAAGGAGCTGACAGAC | CTCAAGGCGTTCCTTCTGCTTC |
| Id2 | TCACCAGAGACCTGGACAGAAC | TGCTATCATTCGACATAAGCTCAG |
| Mmp7 | AGGTGTGGAGTGCCAGATGTTG | CCACTACGATCCGAGGTAAGTC |
| Plaur | AGGACTACCGTGCTTCGGGAAT | ACACGGTCTCTGTCAGGCTGAT |
| Ppard | GGACCAGAACACACGCTTCCTT | CCGACATTCCATGTTGAGGCTG |
| Smurf2 | CCAATGCCATCAACCGCCTCAA | GTGCCTATTCGGTCTCTGGACT |
| β-catenin | GTTCGCCTTCATTATGGACTGCC | ATAGCACCCTGTTCCCGCAAAG |
| Skp1a | CTGTGACTATCAAGACCATGCTG | AGGAGGGTCATCTTTGTGGTGG |
| Rbx1 | GTGGTTGATAACTGTGCCATCTG | CATGCAACCGTACACTCTTCGG |
| Fbxw7 | CGAGACTTCATCTCCTTGCTTCC | CCAGAGAAGGTTATCCTCAGCC |
| Fbxw11 | TGCCTCCAGTATGATGAGCGAG | GTCCATTGCTGAAGCGTAAGTGC |
| Ccne1 | AAGCCCTCTGACCATTGTGTCC | CTAAGCAGCCAACATCCAGGAC |
| Cdt1 | CTCCACAATCGCTCTGAGACTG | GGAAGCGATACGACGTGGGATA |
| E2f1 | GGATCTGGAGACTGACCATCAG | GGTTTCATAGCGTGACTTCTCCC |
| Cdc25a | CCTACTGATGGCAAGCGTGTCA | CTCATTGCCGAGCCTATCTCTC |
| Cdkn1c | AGCTGAAGGACCAGCCTCTCTC | ACGTCGTTCGACGCCTTGTTCT |
| Rbl2 | TCTCGGTGTCTAAGTGCTGCCT | GTTCTCCTGAACATACCTCACGC |
| Notch1 | GCTGCCTCTTTGATGGCTTCGA | CACATTCGGCACTGTTACAGCC |
| Cdkn1b | AGCAGTGTCCAGGGATGAGGAA | TTCTTGGGCGTCTGCTCCACAG |
| Ccne1 | AAGTCCTGAGCCATGCCAAG | GGGTCTTGCAAAAACACGGC |
| Anapc2 | CAGTGAGGATGATTCTGGAGAGC | GAGGTCTTTGCTGCCATAGATGC |
| Anapc11 | CACTTCCCTCTCCTGTGCTGTG | CCCAGCCAAACAGAAAGCATCC |
| Anapc5 | TGTTCTGCTGGAGCACTCTGTG | GAGGTCAGAGTCCTTTAGGGCA |
| Anapc13 | TGAGGATGTCGCCATTCCACTG | TGTAAGGCCAGGTCTGTCCACT |
| Anapc15 | TCCAAAGCATCGCAGAGAAAGAC | CTTCGGAATCCTCTTCACTGTCC |
| Fzr1 | CTGGAACACTCTGACAGGTCAG | GGTACTTCCACACGAGGATCTG |
| Ccnb1 | AGAGGTGGAACTTGCTGAGCCT | GCACATCCAGATGTTTCCATCGG |
| Ccna2 | TTGTAGGCACGGCTGCTATGCT | GGTGCTCCATTCTCAGAACCTG |
| Hmmr | GCAAAGCCAGTCACTTCTGCAG | AACTCAGCCAGGGCGAGCTTTA |
| Nek2 | GAGCACAGGCTCCTATGTCTGG | TGGACTTGGCAAGGTAGCTCTC |
| Cdc20 | GATGGACGACATCTGGCAAGTG | GTTGCCAGGATATTGGACTGCC |
| Bub1b | GTCCACAGGTTCTCAATGCCCA | TGATGGCGTCTTCACTCAGAGG |
| Plk1 | CCATCTTCTGGGTCAGCAAGTG | CCGTCATTGTAGAGAATCAGGCG |
| Cdc6 | CGCAAAGTGTCTGCTGTTTCAGG | GGAGAGTGGTTTGAGGACTGTC |
| Gmnn | GCAGTACATGGCGGAGGTAATC | GCTCCTGAGTCTTCCAGTTCTG |
| Ube2c | GGTGACAAAGGAATCTCCGCCT | GGGAGAGTTTGTACCTCAGGTC |
| Aurka | TCATCCTGGCTCTGAAGGTGCT | CCATACAGCCTGAGGATGTTGG |
| Pclaf | TGCTTGGCTCCTCCACCTTTGT | CCGATGCCTTTTTGCCACTTGG |
| Skil | GAATTGCCATCATGGTATCCTGTT | ATGTGAGCGACACATTCGGTGC |
| Aurkb | CTTCTACGACCAGCAGAGGATC | GGCATCTGACAGTTCCTCCATG |
| Sass6 | GACACATTGGGAACCTCTGCCA | GGAGACAGGATAGCCAATTCCAC |
| Mcph1 | CCACTGGATTTCTGAGGAGCCT | GAGGGTTCCTTGGTATTGCTGG |
| Tfam | GAGGCAAAGGATGATTCGGCTC | CGAATCCTATCATCTTTAGCAAGC |
| Nusap1 | TTCCTCCAAGAGGAAGGCTCTC | GGTGTCTTGGTCAGTGAGCACT |
| Pttg1 | TGCTCCTGATGATGCCTACCCA | ATGAGAGGCACGCCATTCAAGG |
| Skp2 | CCTCGGTCCTTTATGGAGCAGC | CTTGGAGCACTCGGACAGAATC |
| Pfkfb3 | TCATCGAGTCGGTCTGTGACGA | CATGGCTTCTGCTGAGTTGCAG |
| Gls | CAGAAGGCACAGACATGGTTGG | CAAGGTGGCAGCCATCACACTT |
| Opa1 | TCTCAGCCTTGCTGTGTCAGAC | TTCCGTCTCTAGGTTAAAGCGCG |
| Mfn1 | CCAGGTACAGATGTCACCACAG | TTGGAGAGCCGCTCATTCACCT |
| Ube3a | CAGCCTAGTTCAAGGACAGCAG | TCCACATACAACTGCTTCTTCAAG |
| Herc2 | GTCCAGGTTACAGAGTTCTCGG | GCACGCTTCTTGTAAGTCTGGC |
| Hectd3 | ACAGTGTCCTGCACCACCTAGT | GAATCACGCAGGCACTGTGCTA |
| Muc2 | GCCCACCTCACAAGCAGTAT | GTCATAGCCAGGGGCAAACT |
| Clca1 | CTGCCGCTAAAGAGCTTGAGCA | ATCGCCGCATTTCCTGAGGAGA |
| Tff3 | TCCAAGCCAATGTATGGTGCCG | CAGGGCACATTTGGGATACTGG |
| Agr2 | GCAGTTTGTTCTCCTCAACCTGG | GTATCGTCCAGTGATGTCTGCC |

**Table S3. Antibodies for western blot experiments**

| **Antibodies** | **Cat. Numbers** | **Companies** |
| --- | --- | --- |
| EpCAM | ab71916 | Abcam |
| GAPDH | #2118 | CST |
| P-EGFR | #11862 | CST |
| EGFR | ab32562 | Abcam |
| P-ERK1/2 | #4695 | CST |
| ERK1/2 | ET1601-29 | HUABIO |
| P-AKT | #4060 | CST |
| AKT | #9270 | CST |
| Cyclin D1 | ab134175 | Abcam |
| Cyclin E1 | ab133266 | Abcam |
| Histone 3 | ab176880 | Abcam |
| p-Histone 3 | #3377 | CST |
| c-MYC | ab32072 | Abcam |
| β-Catenin | 610154 | BD biosciences |
| p-β-Catenin | #9561 | CST |
| GSK3β | #9323 | CST |
| p-GSK3β | #9315 | CST |
| Axin2 | ab109307 | Abcam |
| HNF4A | #38493 | CST |
| HNF4G | 25801-AP | Proteintech |
| LYZ | ab108508 | Abcam |

**Methods**

**Mice**

All animal experiments were approved by the Experimental Animal Ethics Committee of Guangdong Pharmaceutical University (Guangzhou, China) and adhered to the ARRIVE guidelines. The EpCAM^+/-^ mice were generated in our previous report^1^，and were housed in the SPF mouse facility at 25^o^C, 60-65% humidity and 12hr light-dark cycle, with free access to water and food. The males and females of EpCAM^+/-^ were mated to get embryos and pups for experiments. E18.5 embryos were collected from pregnant heterozygous females. Intestinal tissues were harvested from E18.5 embryos and P3 pups respectively.

Gefitinib (MB1112, Meilunbio, Dalian, China) was orally administrated to the pregnant EpCAM^+/-^ females once every 8 hours from 17.5 days after mating, a total of three times, 7.5mg/kg/time. The considerable volume of sterile water was administrated to control pregnant EpCAM^+/-^ females. Then, the small intestines from the E18.5 embryos were collected 8 hours after the last administration for experiments.

**Single cell preparation and sequencing**

**Single-cell suspension preparation**

The intestines from E18.5 embryos of WT, EpCAM^+/-^ and EpCAM^-/-^ of littermate were collected to perform single-cell transcriptomics by the Gene Denovo Biotechnology Company (Guangzhou, China). Intestinal tissues were cut into mud immediately after collect, then digested in 30-50 times larger volume of trypsin solution than the amount of tissue with 37°C water bath. The cell suspension was filtered after digestion, then centrifuged at 1,500rpm for 5min and the supernatants were discarded. The cells were incubated with erythrocyte lysis buffer at room temperature for 5min, then neutralized with an equal volume of PBS and centrifuged at 1,500rpm for 5min. The cells were suspended in PBS after removing the supernatant. A small amount of cell suspension was taken and mixed with an equal volume of 0.4% trypan blue staining solution, and then survival rates were measured using the Countess® II Automated Cell Counter. Finally, the viable cells were adjusted to an ideal concentration (1000~2000 cells/μl) for further experiments.

**GEM generation & Barcoding**

Cellular suspensions were loaded on a 10X Genomics GemCode Single-cell instrument that generates single-cell Gel Bead-In-EMlusion (GEMs). Libraries were generated and sequenced from the cDNAs with Chromium Next GEM Single Cell 3’ Reagent Kits v3.1. Upon dissolution of the Gel Bead in a GEM, primers containing (i) an Illumina® R1 sequence (read 1 sequencing primer), (ii) a 16nt 10x Barcode, (iii) a 10nt Unique Molecular Identifier (UMI), and (iv) a poly-dT primer sequence were released and mixed with cell lysate and Master Mix. Barcoded, full-length cDNAs were then reverse-transcribed from poly-adenylated mRNA.

**GEM-RT clean up & cDNA amplification**

Silane magnetic beads were used to remove leftover biochemical reagents and primers from the post GEM reaction mixture. Full-length, barcoded cDNAs were then amplified by PCR to generate sufficient mass for library construction.

**Library construction**

R1 (read 1 primer sequence) were added to the molecules during GEM incubation. P5, P7, a sample index, and R2 (read 2 primer sequence) were added during library construction via End Repair, Atailing, Adaptor Ligation, and PCR. The final libraries contained the P5 and P7 primers used in Illumina bridge amplification.

**Sequencing**

The Single Cell 3’ Protocol produced Illumina-ready sequencing libraries. A Single Cell 3’ Library comprised standard Illumina paired-end constructs which begin and end with P5 and P7. The Single Cell 3’ 16bp 10x Barcode and 10bp UMI were encoded in Read 1, while Read 2 was used to sequence the cDNA fragment. Sample index sequences were incorporated as the i7 index read. Read 1 and Read 2 were standard Illumina® sequencing primer sites used in paired-end sequencing.

**Bioinformatic analysis**

**Data quality control and gene expression quantification**

10X Genomics Cell Ranger software (version 3.1.0) was used to convert raw BCL files to FASTQ files, alignment and counts quantification.

**Data quality control and genome alignment**

Briefly, reads with low-quality barcodes and UMIs were filtered out and then mapped to the reference genome. Reads uniquely mapped to the transcriptome and intersecting an exon at least 50% were considered for UMI counting.

**Gene expression quantification**

Before quantification, the UMI sequences would be corrected for sequencing errors, and valid barcodes were identified based on the EmptyDrops method ^2^ . The cell by gene matrices were produced via UMI counting and cell barcodes calling.

The cell by gene matrices for each sample were individually imported to Seurat (version 3.1.1) ^3^ for downstream analysis.

**Normalizing the data**

After removing unwanted cells from the dataset, we employed a global-scaling normalization method “LogNormalize” that normalizes the gene expression measurements for each cell by the total expression, multiplies this by a scale factor (10,000 by default), and log-transforms the results.

**Batch effect correction**

To minimize the effects of batch effect and behavioral conditions on clustering, we used Seurat, which utilized canonical correlation analysis and mutual nearest neighbor analysis, to aggregate all samples ^4^ . 2,000 highly variable genes were selected in each sample based on a variance stabilizing transformation. Anchors between individual data were identified and correction vectors were calculated to generate an integrated expression matrix, which was used for subsequent clustering.

**PCA（Principal component analysis）**

Integrated expression matrix is then scaled and performed on principal component analysis for dimensional reduction. Then, we implemented a resampling test inspired by the jackStraw procedure. We randomly permuted a subset of the data (1% by default) and rerun PCA, constructing a ‘null distribution’ of gene scores, and repeated this procedure. We identified ‘significant’ PCs as those who have a strong enrichment of low p-value genes for downstream clustering and dimensional reduction ^5^ .

**Cells clustering**

Seurat implements a graph-based clustering approach. Distances between the cells were calculated based on previously identified PCs. Briefly, Seurat embed cells in a shared-nearest neighbor (SNN) graph, with edges drawn between cells via similar gene expression patterns. To partition this graph into highly interconnected quasicliques or communities, we first constructed the SNN graph based on the Euclidean distance in PCA space and refined the edge weights between any two cells based on the shared overlap in their local neighborhoods (Jaccard distance). We then applied modularity optimization techniques – SLM ^6^ , to iteratively group cells together, with the goal of optimizing the standard modularity function.

**Clusters visualization**

For visualization of clusters, t-distributed Stochastic Neighbor Embedding (t-SNE) were generated using the same PCs ^7^ .

**Cell type annotation**

The log-normalized matrices were then loaded on SingleR R packages for cell type annotation, which based on correlating gene expression of reference cell types with single-cell expression. First, a Spearman coefficient is calculated for single-cell expression with each of the samples in the reference data set. Next, multiple correlation coefficients per cell type are aggregated to provide a single value per cell type per single cell. Finally, SinlgeR reruns the correlation analysis, but only for the top cell types from the previous step. The analysis was performed only on variable genes. The cell type corresponding to the top value after the last run is assigned to the single cell ^8^ .

**Differentially expressed genes analysis**

Expression value of each gene in given cluster were compared against the rest of cells using Wilcoxon rank sum test ^9^ . Significant upregulated genes were identified using a number of criteria. First, genes had to be at least 1.28-fold overexpressed in the target cluster. Second, genes had to be expressed in more than 25% of the cells belonging to the target cluster. Third, p value is less than 0.05.

**GO enrichment analysis and Pathway enrichment analysis**

For gene ontology (GO) enrichment analysis, all peak related genes were mapped to GO terms in the Gene Ontology database (http://www.geneontology.org/), gene numbers were calculated for every term, significantly enriched GO terms in differentially expressed genes comparing to the genome background were defined by hypergeometric test. The calculated p-value were gone through FDR Correction, taking FDR ≤ 0.05 as a threshold. GO terms meeting this condition were defined as significantly enriched GO terms in differentially expressed genes.

Pathway enrichment analysis was performed through KEGG database ^10^ to identify significantly enriched metabolic pathways or signal transduction pathways in differentially expressed genes comparing with the whole genome background ^11^ . The calculated p-value was gone through FDR Correction, taking FDR ≤ 0.05 as a threshold. Pathways meeting this condition were defined as significantly enriched pathways in differentially expressed genes.

**Cell cycle analysis**

The Seurat R package was used to assign a cell cycle score to each cell based on the 100 marker genes for G1/S phase, 113 marker genes for S phase, 133 marker genes for G2/M phase, 151 marker genes for M phase and 106 marker genes for M/G1 phase ^12^ . Cells with the highest score less than 0.3 was identified as non-cycling cells ^13^ .

**Pesudo time trajectory analysis**

The single-cell trajectory was reconstructed using Monocle2 (Version 2.6.4) (<https://github.com/cole-trapnell-lab/monocle-resease>) via the RGE (Reversed Graph Embedding) machine learning strategy. Genes used for cell ordering were selected with dpFeature. DDRTree incorporated in the reduceDimension function was applied for data dimension reduction.

**Hematoxylin and eosin (H&E) staining and Periodic Acid-Schiff (PAS) staining**

The duodenum, jejunum, ileum and colon from E18.5 embryos and pups at P3 stage of WT and EpCAM^-/-^ mice were fixed in 4% paraformaldehyde in PBS at 4˚C for overnight and then were embedded in paraffin. For H&E staining, the 4-µm-thick paraffin sections were stained with hematoxylin (H9627, Sigma-Aldrich) for 3 min and then followed with eosin (E4009, Sigma-Aldrich) for 20 sec at room temperature. The PAS staining was performed according to the protocols of the Kit (Beijing Leagene Biotechnology Co, Ltd.) also using 4-µm-thick paraffin sections. The images were taken using the Olympus D74 microscope.

**qRT-PCR**

Total RNA was extracted from each small intestinal tissue of E18.5 embryos via Trizol reagent (Invitrogen; Thermo Fisher Scientific, Inc.), and then was subjected to reverse transcription through the PrimeScript™ RT Reagent kit (Takara Bio, Inc.) at 37˚C for 15 min and then 85˚C for 5 sec. qPCR was performed using the SYBR Premix Ex Taq kit (Takara Bio, Inc.) in the LightCycler 480II System (Roche, Inc.). The processes of cycling were as followed: 95˚C for 30 sec; and then 40 cycles of 95˚C for 5 sec, 60˚C for 20 sec and 65˚C for 15 sec. GAPDH was used as the reference. All primers used for qPCR in the present study were listed in the Supplementary Table 2.

**Western blot**

The small intestinal tissues of E18.5 embryos were lysed using the Radio-Immunoprecipitation Assay lysis buffer (MA0151, Dalian Meilun Biotechnology co., Ltd., Dalian, China), centrifuged at 13,680 x g, 4˚C, for 30 min, then the supernatant was harvested. Protein concentration was measured via the BCA kit (P0011, Beyotime, Shanghai, China). Equal amounts of protein were separated through the SDS-PAGE, subsequently transferred to a PVDF membrane. The PVDF membrane was blocked using the 5% skimmed milk (0040895, Biosharp, Hefei, China) in TBST buffer at room temperature for 1 h, subsequently incubated with primary antibodies at 4^o^C for overnight, and then incubated with horseradish peroxidase-labeled secondary antibodies. The signals were detected using the enhanced chemiluminescence reagent. The quantification of western blot bands was analyzed using the Lane 1d software (version 5.1.0.0; SageCreation). The primary and secondary antibodies were listed in the Supplementary Table 3.

**Immunofluorescence (IF) staining**

The jejunum and colon segments of mice were positioned within a grid-embedding container and immersed in 4% paraformaldehyde solution at 4°C for overnight fixation. Subsequently, dehydration was carried out following a standardized sucrose gradient protocol. For tissue embedding, optimal cutting temperature compound (OCT) (Sakura Finetek) was used and sections of 7μm thickness were obtained using a cryostat. To facilitate antigen retrieval, the sections were boiled in 10 mM sodium citrate buffer (pH 6.0). After that, a one-hour blocking step was performed at room temperature using goat serum. The samples were then treated with primary antibody and incubated overnight at 4°C, followed by exposure to the secondary antibody for one hour at room temperature. The primary antibody used for immunofluorescence staining was rabbit anti-EGFR (1:200; ab52894; Abcam). The secondary antibody used for the immunofluorescence staining was the Alexa Fluor 488-labeled donkey anti-rabbit IgG (H+L) secondary antibody (Thermo Fisher Scientiffc, Inc; A21206). Images were obtained through the Olympus FV3000 Confocal microscope.

**Statistical Analysis**

Statistical analysis was determined via the SPSS software (version 25.0; IBM Corp.). Mean ± SD was used to express data. Student's t test was conducted to compare difference between two groups. The P-value <0.05 was considered to be significant.

**References to Supplemental Material**

1. Yang, Y. *et al.* Circular RNA profile in liver tissue of EpCAM knockout mice. *Int J Mol Med* **44**, 1063-1077 (2019).

2. Lun, A. T. L. *et al.* EmptyDrops: distinguishing cells from empty droplets in droplet-based single-cell RNA sequencing data. *Genome Biol* **20**, 63 (2019).

3. Butler, A., Hoffman, P., Smibert, P., Papalexi, E. & Satija, R. Integrating single-cell transcriptomic data across different conditions, technologies, and species. *Nat Biotechnol* **36**, 411-420 (2018).

4. Stuart, T. *et al.* Comprehensive Integration of Single-Cell Data. *Cell* **177**, 1888-1902.e1821 (2019).

5. Chung, N. C. & Storey, J. D. Statistical significance of variables driving systematic variation in high-dimensional data. *Bioinformatics* **31**, 545-554 (2015).

6. Waltman, L. & van Eck, N. J. A smart local moving algorithm for large-scale modularity-based community detection. *The European Physical Journal B* **86** (2013).

7. Van der Maaten, L. & Hinton, G. Visualizing data using t-SNE. *Journal of machine learning research* **9** (2008).

8. Aran, D. *et al.* Reference-based analysis of lung single-cell sequencing reveals a transitional profibrotic macrophage. *Nat Immunol* **20**, 163-172 (2019).

9. Camp, J. G. *et al.* Multilineage communication regulates human liver bud development from pluripotency. *Nature* **546**, 533-538 (2017).

10. Kanehisa, M. & Goto, S. KEGG: kyoto encyclopedia of genes and genomes. *Nucleic acids research* **28**, 27-30 (2000).

11. Ashburner, M. *et al.* Gene Ontology: tool for the unification of biology. *Nature Genetics* **25**, 25-29 (2000).

12. Macosko, E. Z. *et al.* Highly Parallel Genome-wide Expression Profiling of Individual Cells Using Nanoliter Droplets. *Cell* **161**, 1202-1214 (2015).

13. Neftel, C. *et al.* An Integrative Model of Cellular States, Plasticity, and Genetics for Glioblastoma. *Cell* **178**, 835-849.e821 (2019).
