# Supplementary material for: Single-cell transcriptomics reveals EpCAM regulates the development and morphology of intestinal epithelium via controlling the EGFR pathway
Source: Genes Dis. 2026 Feb 9;13(5):102072. doi: 10.1016/j.gendis.2026.102072 (PMC13157056; doi:10.1016/j.gendis.2026.102072)
Supplement: Multimedia component 3 [file mmc3.docx]

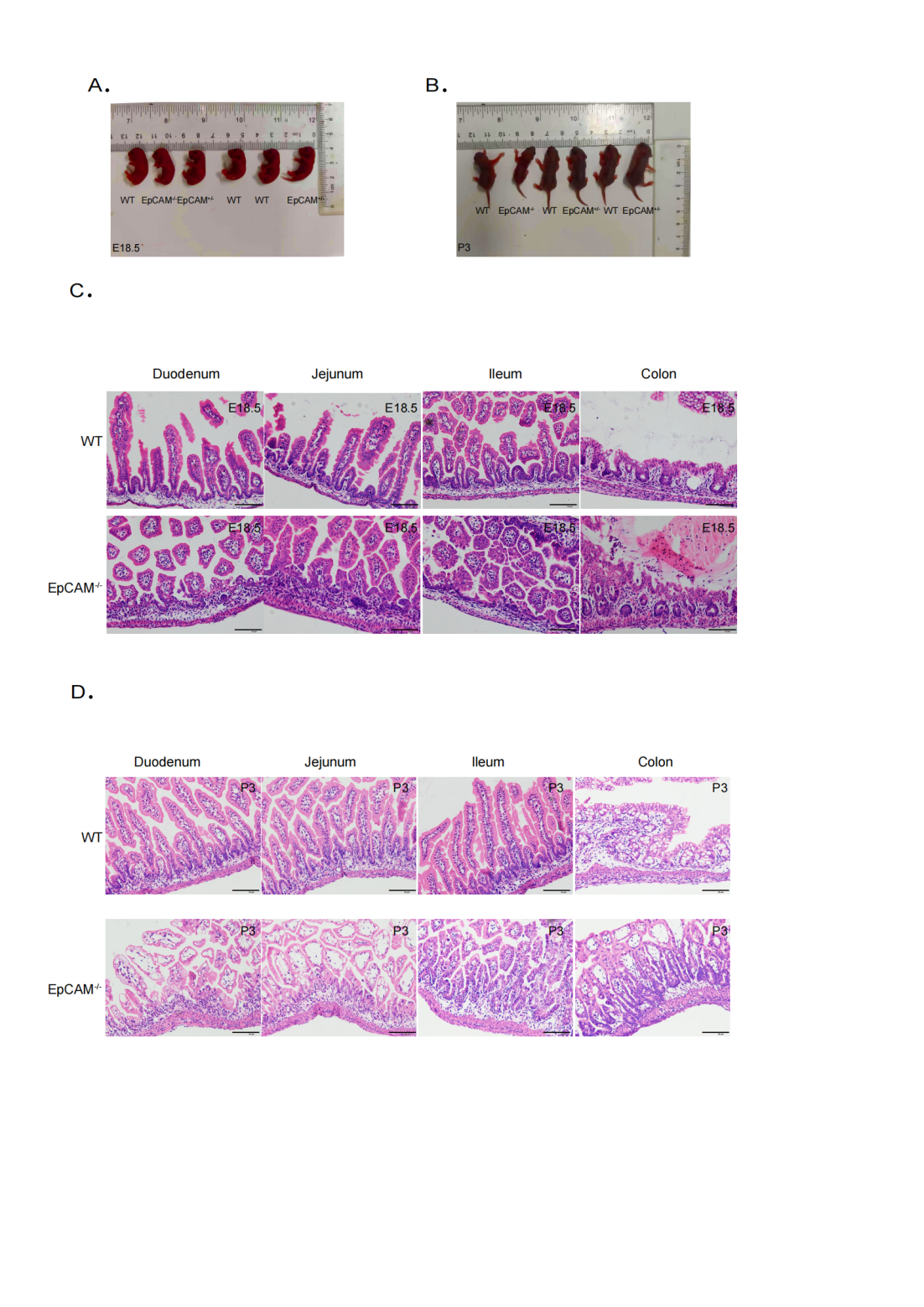


**Figure S1. The Deficiency of EpCAM Caused the Breakdown of Homeostasis of the Intestinal Epithelium from Mice**

**A**. A littermate of E18.5 embryos from one pair of EpCAM^+/-^ parental mice. **B**. A littermate of P3 pups from one pair of EpCAM^+/-^ parental mice. **C**. Images of H&E staining of the duodenum, jejunum, ileum and colon from WT and EpCAM^-/-^ embryos at E18.5 stage. **D**. Images of H&E staining of the duodenum, jejunum, ileum and colon from WT and EpCAM^-/-^ pups at P3 stage. Scale bar, 50μm.
