# Supplementary material for: Single-cell transcriptomics reveals EpCAM regulates the development and morphology of intestinal epithelium via controlling the EGFR pathway
Source: Genes Dis. 2026 Feb 9;13(5):102072. doi: 10.1016/j.gendis.2026.102072 (PMC13157056; doi:10.1016/j.gendis.2026.102072)
Supplement: Multimedia component 4 [file mmc4.docx]

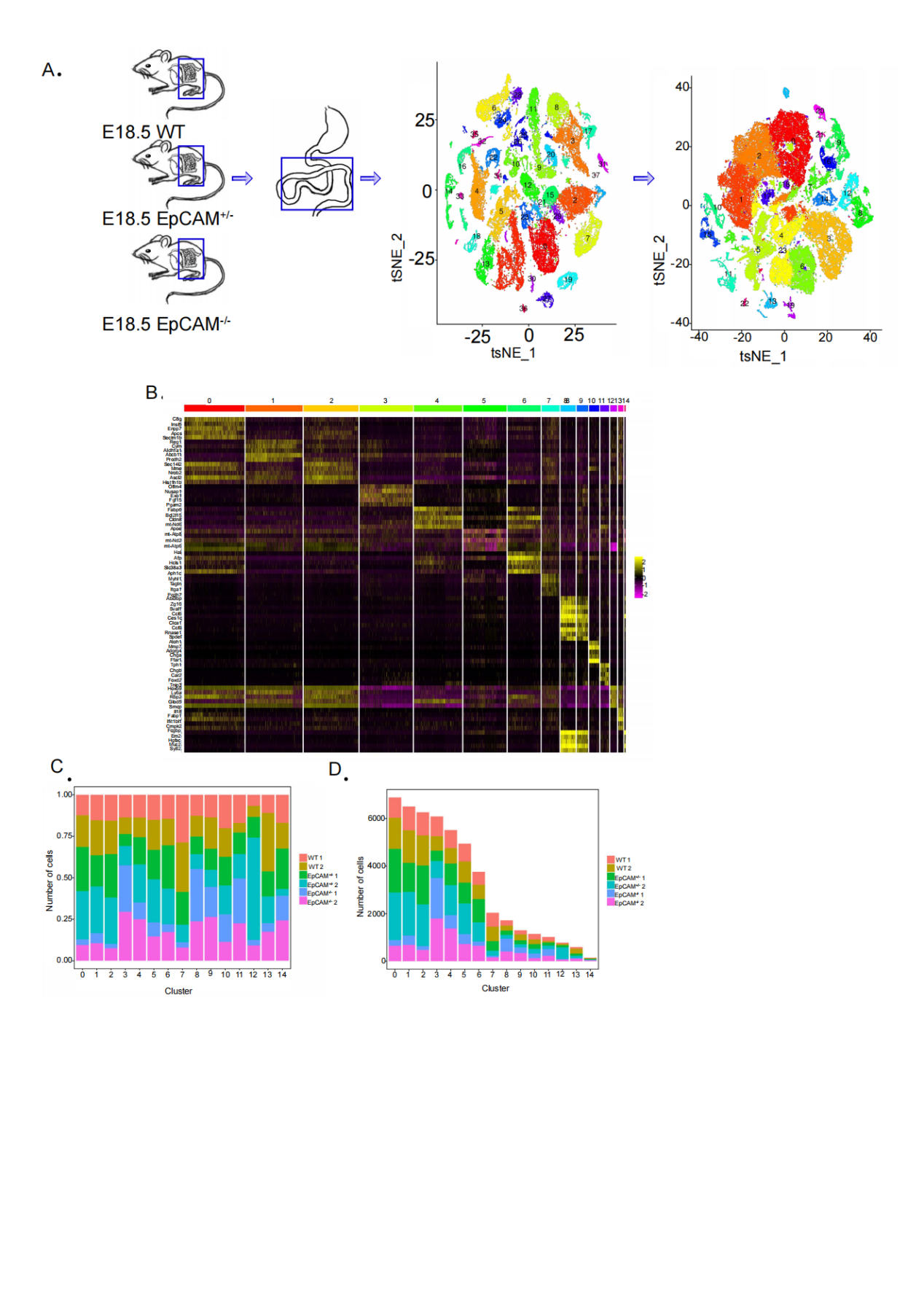


**Figure S2. The Single-cell Expression Atlas of Intestines of E18.5 Embryos from Mating of EpCAM^+/-^ Parental Mice**

**A**. Schematic of the experimental approach. **B**. Heatmap showed top 5 genes of each cluster. **C**. Ratios of IECs in each cluster of different intestinal samples. **D**. Numbers of IECs in each cluster of different intestinal samples.
