# Supplementary material for: Single-cell transcriptomics reveals EpCAM regulates the development and morphology of intestinal epithelium via controlling the EGFR pathway
Source: Genes Dis. 2026 Feb 9;13(5):102072. doi: 10.1016/j.gendis.2026.102072 (PMC13157056; doi:10.1016/j.gendis.2026.102072)
Supplement: Multimedia component 5 [file mmc5.docx]

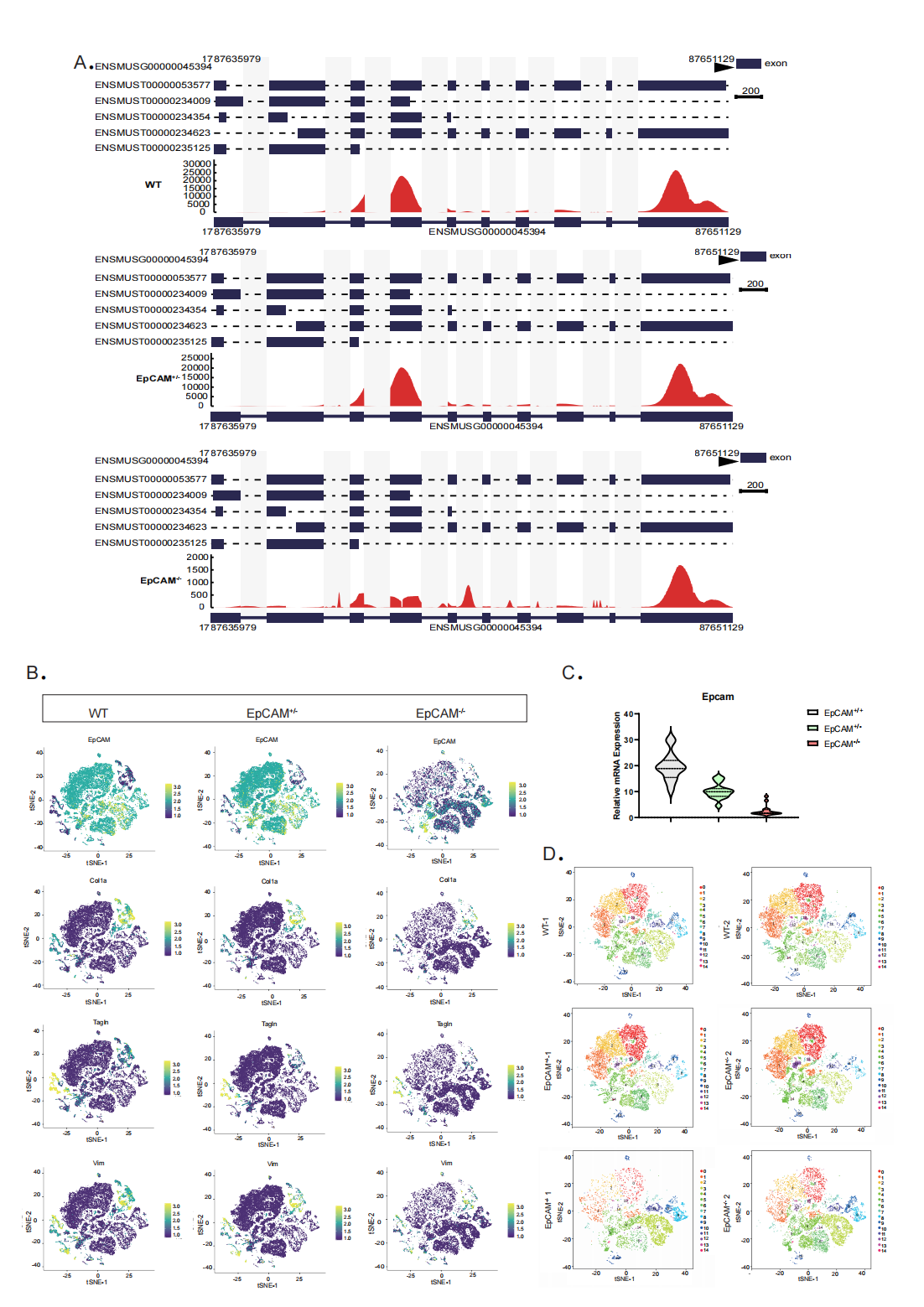


**Figure S3. Overview of 15 Clusters of Intestinal Epithelial Cells from E18.5 Embryos of Different Genotypes**

**A**. The mRNAs of WT and mutant EpCAM could be detected by single-cell RNA-seq technique. **B**. The tSNE plots showed the expression of EpCAM, Col1a, Tagln and Vim in each cluster of intestinal cells from 2 WT, 2 EpCAM^+/-^, and 2 EpCAM^-/-^ E18.5 embryos respectively. **C**. Relative mRNA expression levels of EpCAM and mutant EpCAM in the intestinal epithelial cell from WT, EpCAM^+/-^ and EpCAM^-/-^ embryos respectively. **D**. The tSNE plots showed the distribution of intestinal epithelial cells from each embryo of WT, EpCAM^+/-^ and EpCAM^-/-^ in the 15 clusters.
