# Supplementary material for: Single-cell transcriptomics reveals EpCAM regulates the development and morphology of intestinal epithelium via controlling the EGFR pathway
Source: Genes Dis. 2026 Feb 9;13(5):102072. doi: 10.1016/j.gendis.2026.102072 (PMC13157056; doi:10.1016/j.gendis.2026.102072)
Supplement: Multimedia component 6 [file mmc6.docx]

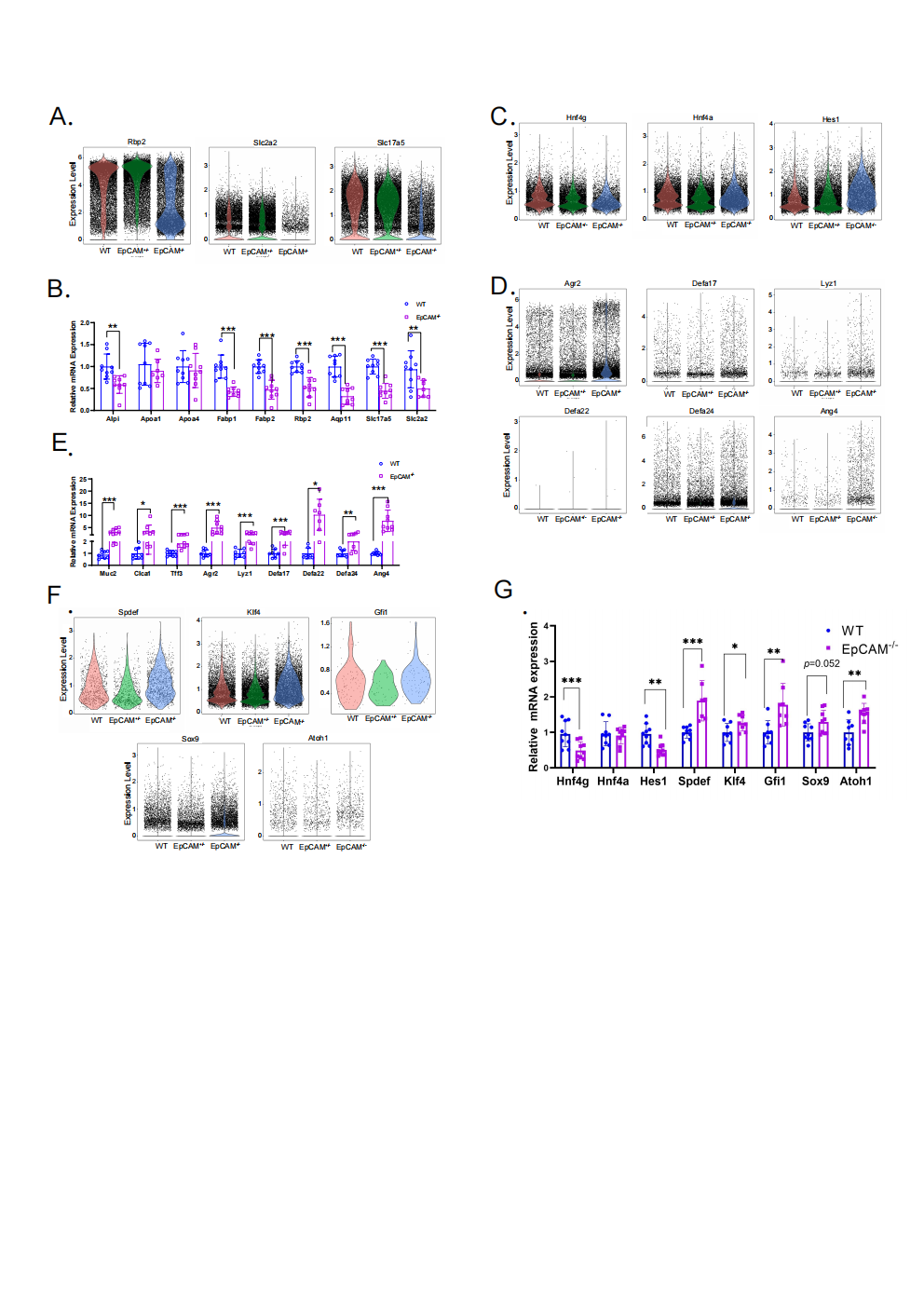


**Figure S4.** **The Deficiency of EpCAM Affected the Differential Potential of E18.5 Intestinal Stem Cells into Absorptive and Secretory Intestinal Epithelial Cells**

**A**. Violin plots compared the expression levels of Rbp2, Slc2a2 and Slc17a5 in the IECs from WT (Red), EpCAM^+/-^(Green) and EpCAM^-/-^ (Blue) E18.5 embryos. **B**. The qPCR results of Alpi, Apoa1, Apoa4, Fabp1, Fabp2, Rbp2, Aqp11, Slc17a5 and Slc2a2 from the small intestines of WT and EpCAM^-/-^ embryos at E18.5 stage. **C**. Violin plots compared the expression levels of Hnf4g, Hnf4a and Hes1 in the IECs from each genotype. **D**. Violin plots compared the expression levels of Agr2, Defa17, Lyz1, Defa22, Defa24 and Ang4 in the IECs from each genotype. **E**. The qPCR results of Muc2, Clca1, Tff3, Agr2, Lyz1, Defa17, Defa22, Defa24 and Ang4 from the small intestines of each group. **F**. Violin plots compared the expression levels of Spdef, Klf4, Gfi1, Sox9 and Atoh1 in the IECs from each genotype. **G**. The qPCR results of *Hnf4g*, *Hnf4a*, *Hes1*, *Spdef*, *Klf4*, *Gfi1*, *Sox9* and *Atoh1* from the small intestines of WT and EpCAM^-/-^ groups. *p<0.05, **p<0.01, ***p<0.001.
