# Supplementary material for: Single-cell transcriptomics reveals EpCAM regulates the development and morphology of intestinal epithelium via controlling the EGFR pathway
Source: Genes Dis. 2026 Feb 9;13(5):102072. doi: 10.1016/j.gendis.2026.102072 (PMC13157056; doi:10.1016/j.gendis.2026.102072)
Supplement: Multimedia component 8 [file mmc8.docx]

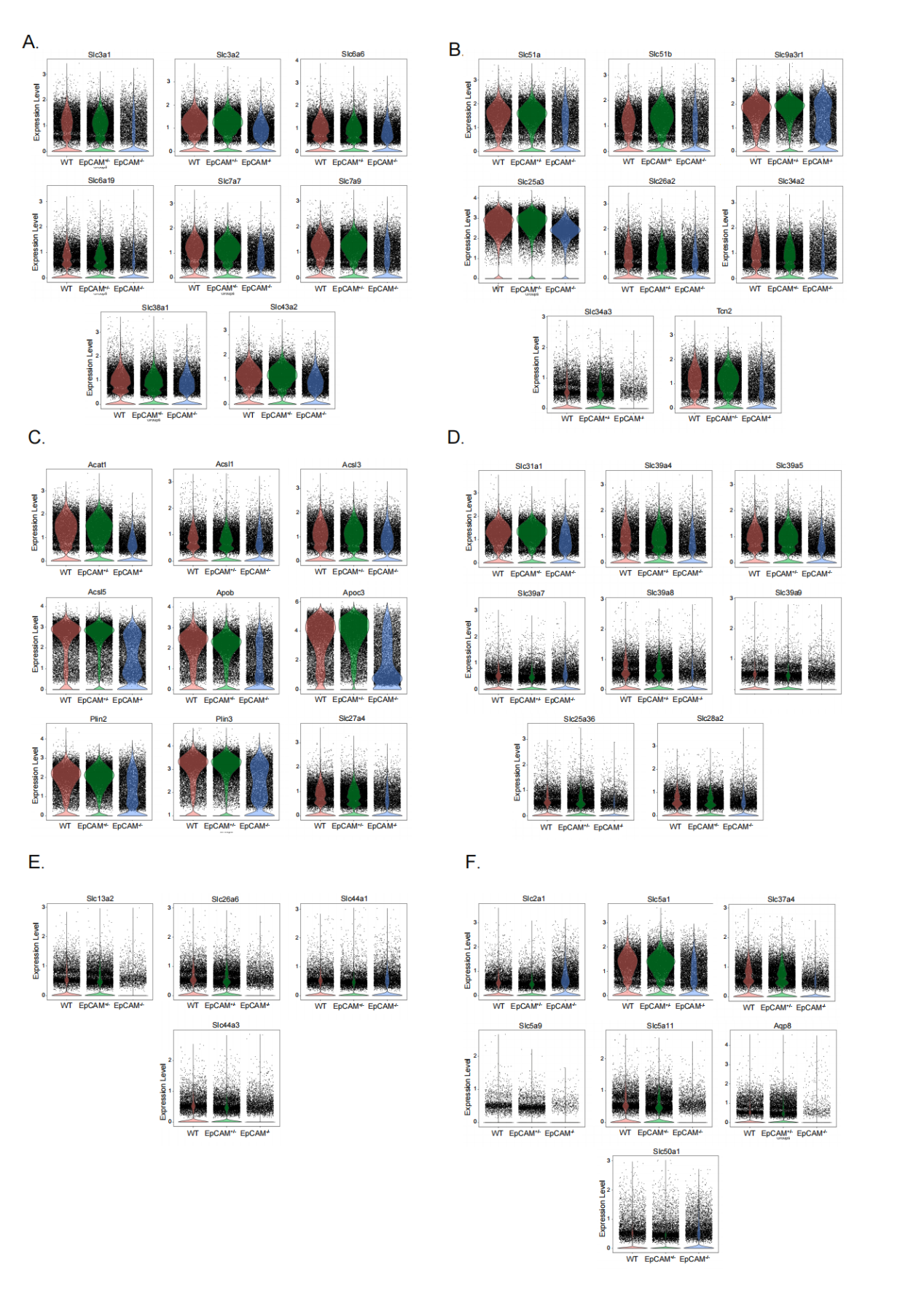


**Figure S6. The Deficiency of EpCAM Caused the Down-regulation of Genes Related to Nutrient Absorption in the Intestinal Epithelial Cells of E18.5 Embryonic Mice**

**A**. Violin plots compared the mRNA levels of genes related to the absorption of amino acids (Slc3a1, Slc3a2, Slc6a6, Slc6a19, Slc7a7, Slc7a9, Slc38a1 and Slc43a2) in the intestinal epithelial cells from WT (Red), EpCAM^+/-^(Green) and EpCAM^-/-^ (Blue) E18.5 embryos. **B**. Violin plots compared the mRNA levels of genes related to the absorption of bile salts (Slc51a and Slc51b), inorganic solutes (Slc9a3r1, Slc25a3, Slc26a2, Slc34a2 and Slc34a3) and vitamin (Tcn2) in the intestinal epithelial cells from WT (Red), EpCAM^+/-^(Green) and EpCAM^-/-^ (Blue) E18.5 embryos. **C**. Violin plots compared the mRNA levels of genes related to the absorption of lipids (Acat1, Acsl1, Acsl3, Acsl5, Apob, Apoc3, Plin2, Plin3 and Slc27a4) in the intestinal epithelial cells from WT (Red), EpCAM^+/-^(Green) and EpCAM^-/-^ (Blue) E18.5 embryos. **D**. Violin plots compared the mRNA levels of genes related to the absorption of metal ions (Slc31a1, Slc39a4, Slc39a5, Slc39a7, Slc39a8 and Slc39a9) and nucleotides (Slc25a36 and Slc28a2) in the intestinal epithelial cells from WT (Red), EpCAM^+/-^(Green) and EpCAM^-/-^ (Blue) E18.5 embryos. **E**. Violin plots compared the mRNA levels of genes related to the absorption of organic solutes (Slc13a2, Slc26a6, Slc44a1 and Slc44a3) in the intestinal epithelial cells from WT (Red), EpCAM^+/-^(Green) and EpCAM^-/-^ (Blue) E18.5 embryos. **F**. Violin plots compared the mRNA levels of genes related to the absorption of sugars (Slc2a1, Slc5a1, Slc5a9, Slc5a11, Slc37a4 and Slc50a1) and waters (Aqp8) in the intestinal epithelial cells from WT (Red), EpCAM^+/-^(Green) and EpCAM^-/-^ (Blue) E18.5 embryos.
