# Supplementary material for: Single-cell transcriptomics reveals EpCAM regulates the development and morphology of intestinal epithelium via controlling the EGFR pathway
Source: Genes Dis. 2026 Feb 9;13(5):102072. doi: 10.1016/j.gendis.2026.102072 (PMC13157056; doi:10.1016/j.gendis.2026.102072)
Supplement: Multimedia component 9 [file mmc9.docx]

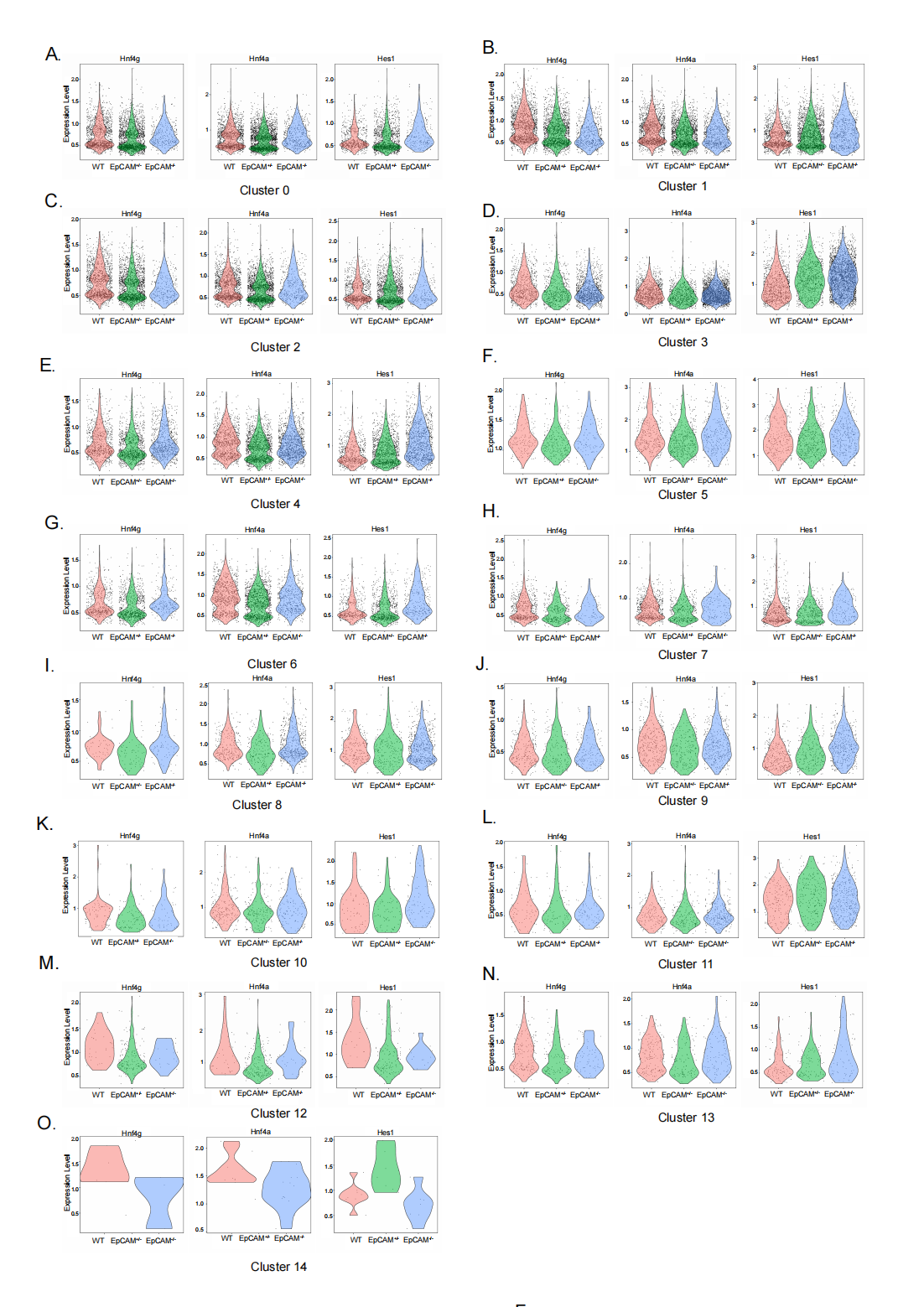


**Figure S7. Comparison of the expression of genes encoding transcriptional factors which regulate the differentiation of enterocytes in the intestinal epithelial cells from WT, EpCAM^+/-^ and EpCAM^-/-^ mice**

**A**. Violin plots compared the mRNA levels of Hnf4g, Hnf4a and Hes1 in the intestinal epithelial cells from Cluster 0 of WT, EpCAM^+/-^ and EpCAM^-/-^ mice; **B**. Violin plots compared the mRNA levels of Hnf4g, Hnf4a and Hes1 in the intestinal epithelial cells from Cluster 1 of WT, EpCAM^+/-^ and EpCAM^-/-^ mice. **C**. Violin plots compared the mRNA levels of Hnf4g, Hnf4a and Hes1 in the intestinal epithelial cells from Cluster 2 of WT, EpCAM^+/-^ and EpCAM^-/-^ mice. **D**. Violin plots compared the mRNA levels of Hnf4g, Hnf4a and Hes1 in the intestinal epithelial cells from Cluster 3 of WT, EpCAM^+/-^ and EpCAM^-/-^ mice. **E**. Violin plots compared the mRNA levels of Hnf4g, Hnf4a and Hes1 in the intestinal epithelial cells from Cluster 4 of WT, EpCAM^+/-^ and EpCAM^-/-^ mice. **F**. Violin plots compared the mRNA levels of Hnf4g, Hnf4a and Hes1 in the intestinal epithelial cells from Cluster 5 of WT, EpCAM^+/-^ and EpCAM^-/-^ mice. **G**. Violin plots compared the mRNA levels of Hnf4g, Hnf4a and Hes1 in the intestinal epithelial cells from Cluster 6 of WT, EpCAM^+/-^ and EpCAM^-/-^ mice; **H**. Violin plots compared the mRNA levels of Hnf4g, Hnf4a and Hes1 in the intestinal epithelial cells from Cluster 7 of WT, EpCAM^+/-^ and EpCAM^-/-^ mice. **I**. Violin plots compared the mRNA levels of Hnf4g, Hnf4a and Hes1 in the intestinal epithelial cells from Cluster 8 of WT, EpCAM^+/-^ and EpCAM^-/-^ mice. **J**. Violin plots compared the mRNA levels of Hnf4g, Hnf4a and Hes1 in the intestinal epithelial cells from Cluster 9 of WT, EpCAM^+/-^ and EpCAM^-/-^ mice. **K**. Violin plots compared the mRNA levels of Hnf4g, Hnf4a and Hes1 in the intestinal epithelial cells from Cluster 10 of WT, EpCAM^+/-^ and EpCAM^-/-^ mice. **L**. Violin plots compared the mRNA levels of Hnf4g, Hnf4a and Hes1 in the intestinal epithelial cells from Cluster 11 of WT, EpCAM^+/-^ and EpCAM^-/-^ mice. **M**. Violin plots compared the mRNA levels of Hnf4g, Hnf4a and Hes1 in the intestinal epithelial cells from Cluster 12 of WT, EpCAM^+/-^ and EpCAM^-/-^ mice. **N**. Violin plots compared the mRNA levels of Hnf4g, Hnf4a and Hes1 in the intestinal epithelial cells from Cluster 13 of WT, EpCAM^+/-^ and EpCAM^-/-^ mice. **O**. Violin plots compared the mRNA levels of Hnf4g, Hnf4a and Hes1 in the intestinal epithelial cells from Cluster 14 of WT, EpCAM^+/-^ and EpCAM^-/-^ mice.
