# Supplementary material for: Single-cell transcriptomics reveals EpCAM regulates the development and morphology of intestinal epithelium via controlling the EGFR pathway
Source: Genes Dis. 2026 Feb 9;13(5):102072. doi: 10.1016/j.gendis.2026.102072 (PMC13157056; doi:10.1016/j.gendis.2026.102072)
Supplement: Multimedia component 10 [file mmc10.docx]

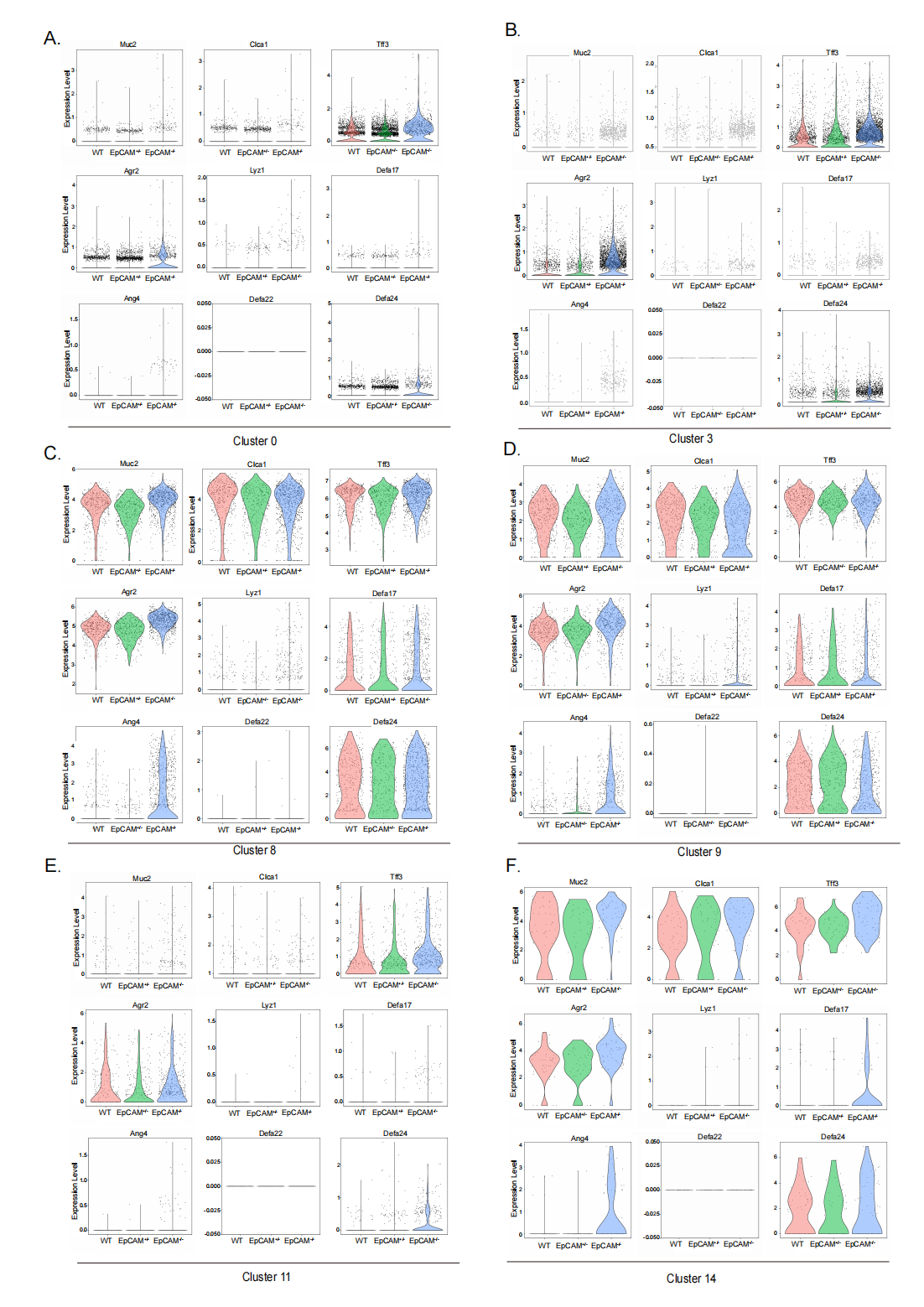


**Figure S8. Comparison of the expression of genes related to markers of goblets and Paneth cells in the intestinal epithelial cells from WT, EpCAM^+/-^ and EpCAM^-/-^ mice**

**A**. Violin plots compared the mRNA levels of Muc2, Clca1, Tff3, Agr2, Lyz1, Defa17, Ang4, Defa22 and Defa24 in the intestinal epithelial cells from Cluster 0 of WT, EpCAM^+/-^ and EpCAM^-/-^ mice; **B**. Violin plots compared the mRNA levels of Muc2, Clca1, Tff3, Agr2, Lyz1, Defa17, Ang4, Defa22 and Defa24 in the intestinal epithelial cells from Cluster 3 of WT, EpCAM^+/-^ and EpCAM^-/-^ mice; **C**. Violin plots compared the mRNA levels of Muc2, Clca1, Tff3, Agr2, Lyz1, Defa17, Ang4, Defa22 and Defa24 in the intestinal epithelial cells from Cluster 8 of WT, EpCAM^+/-^ and EpCAM^-/-^ mice. **D**. Violin plots compared the mRNA levels of Muc2, Clca1, Tff3, Agr2, Lyz1, Defa17, Ang4, Defa22 and Defa24 in the intestinal epithelial cells from Cluster 9 of WT, EpCAM^+/-^ and EpCAM^-/-^ mice. **E**. Violin plots compared the mRNA levels of Muc2, Clca1, Tff3, Agr2, Lyz1, Defa17, Ang4, Defa22 and Defa24 and Ang4 in the intestinal epithelial cells from Cluster 11 of WT, EpCAM^+/-^ and EpCAM^-/-^ mice. **F**. Violin plots compared the mRNA levels of Muc2, Clca1, Tff3, Agr2, Lyz1, Defa17, Ang4, Defa22 and Defa24 in the intestinal epithelial cells from Cluster 14 of WT, EpCAM^+/-^ and EpCAM^-/-^ mice.
