# Supplementary material for: Single-cell transcriptomics reveals EpCAM regulates the development and morphology of intestinal epithelium via controlling the EGFR pathway
Source: Genes Dis. 2026 Feb 9;13(5):102072. doi: 10.1016/j.gendis.2026.102072 (PMC13157056; doi:10.1016/j.gendis.2026.102072)
Supplement: Multimedia component 11 [file mmc11.docx]

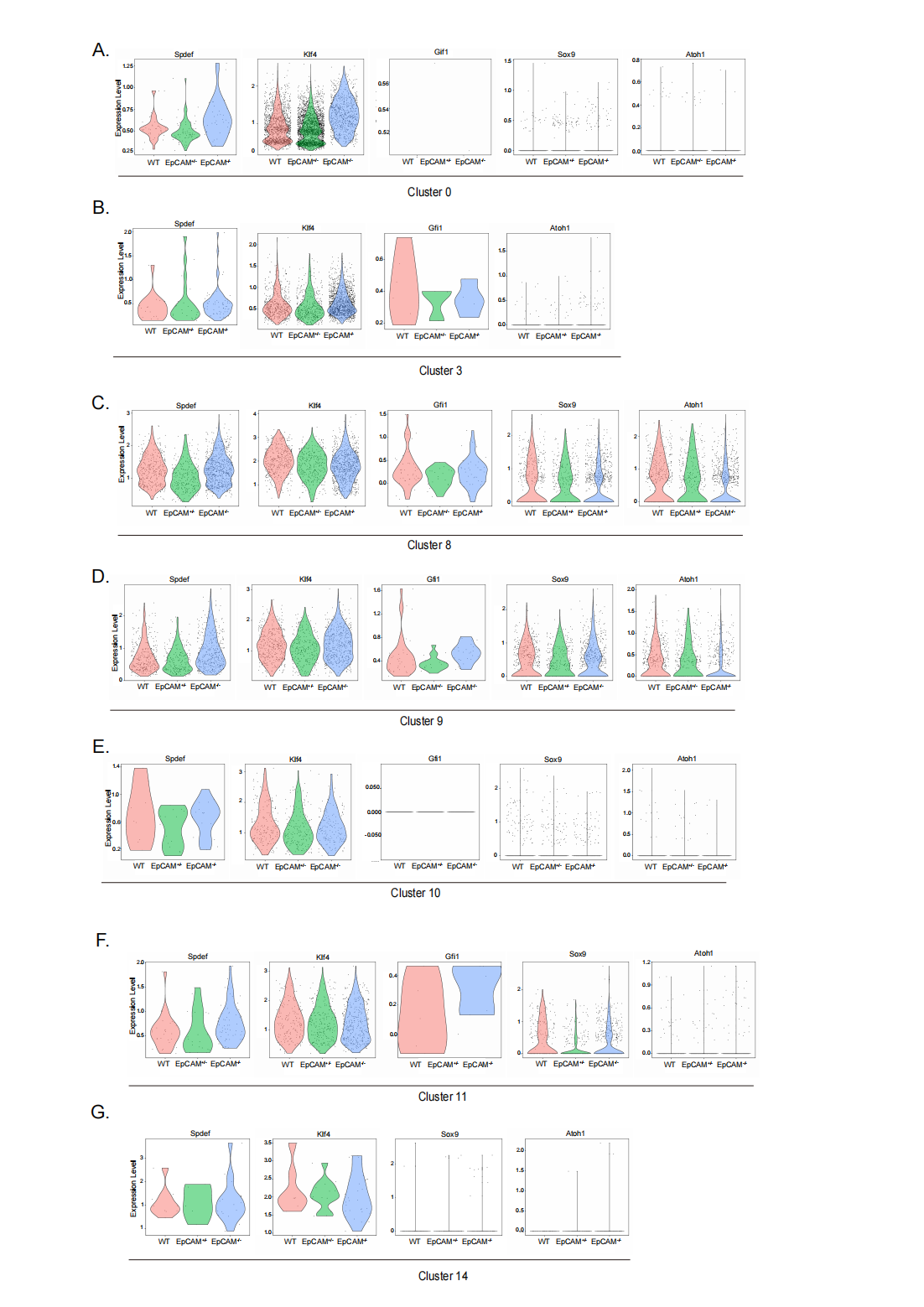


**Figure S9. Comparison of the expression of genes encoding transcriptional factors which regulate the differentiation of secretory cells in the intestinal epithelial cells from WT, EpCAM^+/-^ and EpCAM^-/-^ mice**

**A**. Violin plots compared the mRNA levels of Spdef, Klf4, Gfi1, Sox9 and Atoh1 in the intestinal epithelial cells from Cluster 0 of WT, EpCAM^+/-^ and EpCAM^-/-^ mice; **B**. Violin plots compared the mRNA levels of Spdef, Klf4, Gfi1 and Atoh1 in the intestinal epithelial cells from Cluster 3 of WT, EpCAM^+/-^ and EpCAM^-/-^ mice; **C.** Violin plots compared the mRNA levels of Spdef, Klf4, Gfi1, Sox9 and Atoh1 in the intestinal epithelial cells from Cluster 8 of WT, EpCAM^+/-^ and EpCAM^-/-^ mice. **D**. Violin plots compared the mRNA levels of Spdef, Klf4, Gfi1, Sox9 and Atoh1 in the intestinal epithelial cells from Cluster 9 of WT, EpCAM^+/-^ and EpCAM^-/-^ mice. **E**. Violin plots compared the mRNA levels of Spdef, Klf4, Gfi1, Sox9 and Atoh1 in the intestinal epithelial cells from Cluster 10 of WT, EpCAM^+/-^ and EpCAM^-/-^ mice. **F**. Violin plots compared the mRNA levels of Spdef, Klf4, Gfi1, Sox9 and Atoh1 in the intestinal epithelial cells from Cluster 11 of WT, EpCAM^+/-^ and EpCAM^-/-^ mice. **G**. Violin plots compared the mRNA levels of Spdef, Klf4, Sox9 and Atoh1 in the intestinal epithelial cells from Cluster 14 of WT, EpCAM^+/-^ and EpCAM^-/-^ mice.
