# Supplementary material for: Single-cell transcriptomics reveals EpCAM regulates the development and morphology of intestinal epithelium via controlling the EGFR pathway
Source: Genes Dis. 2026 Feb 9;13(5):102072. doi: 10.1016/j.gendis.2026.102072 (PMC13157056; doi:10.1016/j.gendis.2026.102072)
Supplement: Multimedia component 12 [file mmc12.docx]

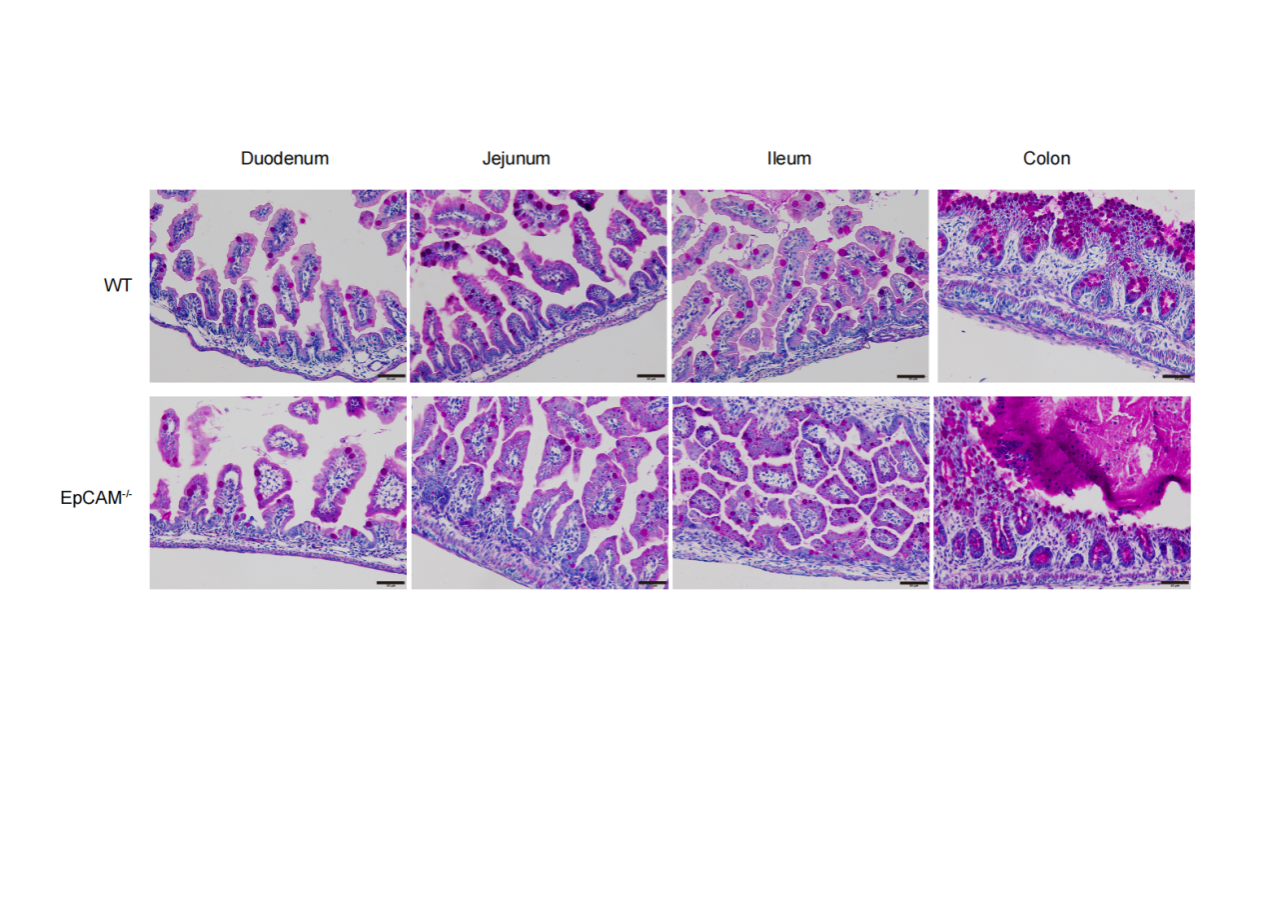


**Figure S10. The deficiency of EpCAM affected the number and size of goblet cells in the intestines of mice**

Images of PAS staining of the duodenum, jejunum, ileum and colon from WT and EpCAM^-/-^ embryos at E18.5 stage. Scale bar, 50μm. PAS, Periodic Acid-Schiff
