# Supplementary material for: Single-cell transcriptomics reveals EpCAM regulates the development and morphology of intestinal epithelium via controlling the EGFR pathway
Source: Genes Dis. 2026 Feb 9;13(5):102072. doi: 10.1016/j.gendis.2026.102072 (PMC13157056; doi:10.1016/j.gendis.2026.102072)
Supplement: Multimedia component 13 [file mmc13.docx]

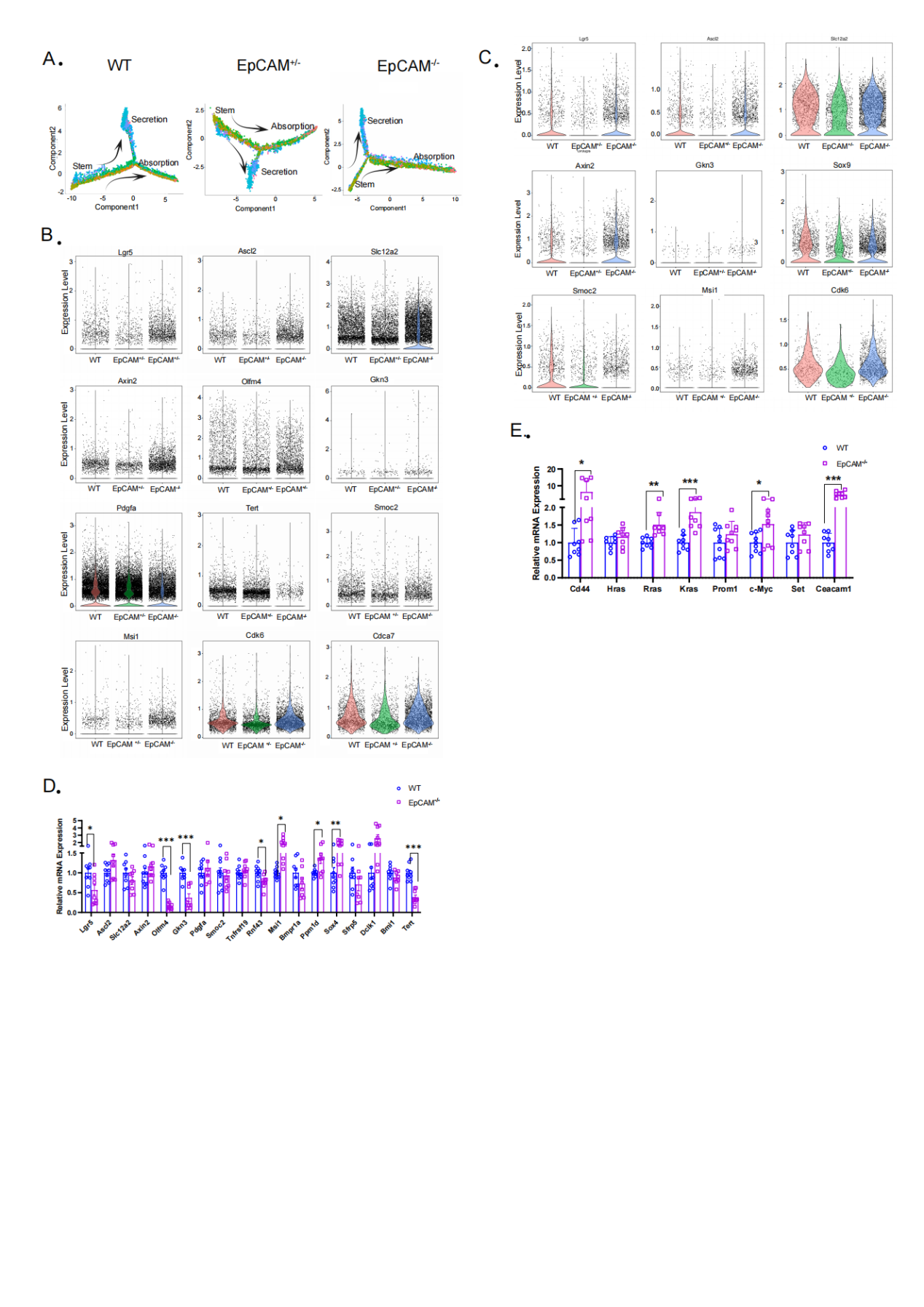


**Figure S11. The Differential Potential and Quantity of Intestinal Stem Cells from E18.5 Embryos of EpCAM Deficient Mice**

**A**. Developmental trajectories of IECs from each genotype. **B**. Violin plots compared the expression levels of Lgr5, Ascl2, Slc12a2, Axin2, Olfm4, Gkn3, Pdgfa, Tert Smoc2, Msi1, Cdk6 and Cdca7 in IECs from each genotype. **C**. Violin plots compared the mRNA levels of Lgr5, Ascl2, Slc12a2, Axin2, Gkn3, Sox9, Smoc2, Msi1 and Cdk6 in the IECs from Cluster 3 of each genotype. **D**. The qPCR results of Lgr5, Ascl2, Slc12a2, Axin2, Olfm4, Gkn3, Pdgfa, Smoc2, Tnfrsf19, Rnf43, Msi1, Bmpr1a, Ppm1d, Sox4, Sfrp5, Dclk1, Bmi1 and Tert from the small intestines of each group. **E**. The qPCR results of Cd44, Hras, Rras, Kras, Prom1, c-Myc Set and Ceacam1 from the small intestines of each group. ^*^p<0.05, ^**^p<0.01, ^***^p<0.001.
