# Supplementary material for: Single-cell transcriptomics reveals EpCAM regulates the development and morphology of intestinal epithelium via controlling the EGFR pathway
Source: Genes Dis. 2026 Feb 9;13(5):102072. doi: 10.1016/j.gendis.2026.102072 (PMC13157056; doi:10.1016/j.gendis.2026.102072)
Supplement: Multimedia component 14 [file mmc14.docx]

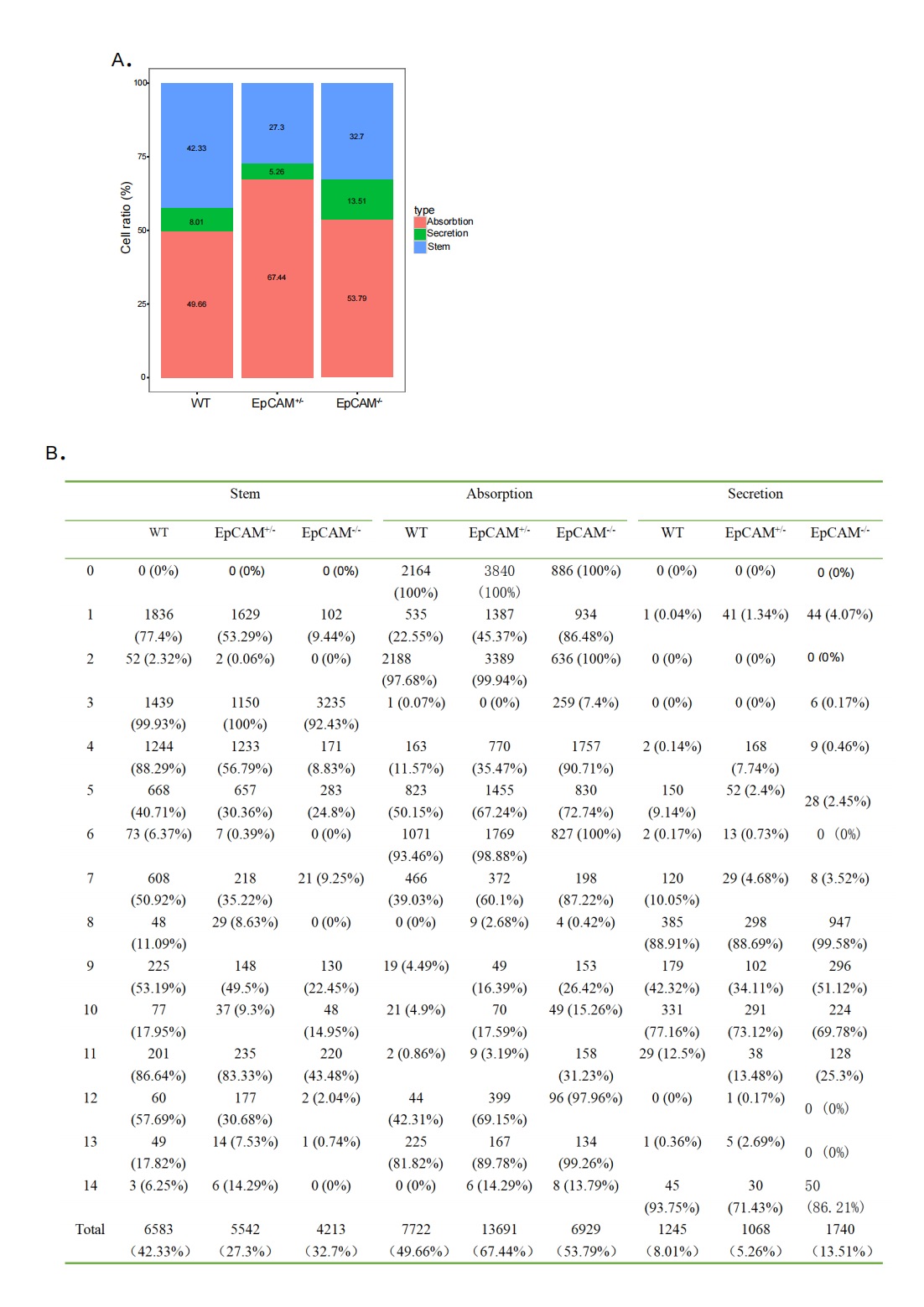


**Figure S12. The numbers and percent of cells in the stem, absorption and secretion phages of the pseudo-time trajectories for the intestinal epithelial cells from WT, EpCAM^+/-^ and EpCAM^-/-^ mice**

Graphs showed the percent of cells in the stem, absorption and secretion phages of the pseudo-time trajectories for the intestinal epithelial cells from WT, EpCAM^+/-^ and EpCAM^-/-^ mice. **B**. Table summary of numbers and percent of cells in the stem, absorption and secretion phages of the pseudo-time trajectories for each cluster of the intestinal epithelial cells from WT, EpCAM^+/-^ and EpCAM^-/-^ mice.
