# Supplementary material for: Single-cell transcriptomics reveals EpCAM regulates the development and morphology of intestinal epithelium via controlling the EGFR pathway
Source: Genes Dis. 2026 Feb 9;13(5):102072. doi: 10.1016/j.gendis.2026.102072 (PMC13157056; doi:10.1016/j.gendis.2026.102072)
Supplement: Multimedia component 15 [file mmc15.docx]

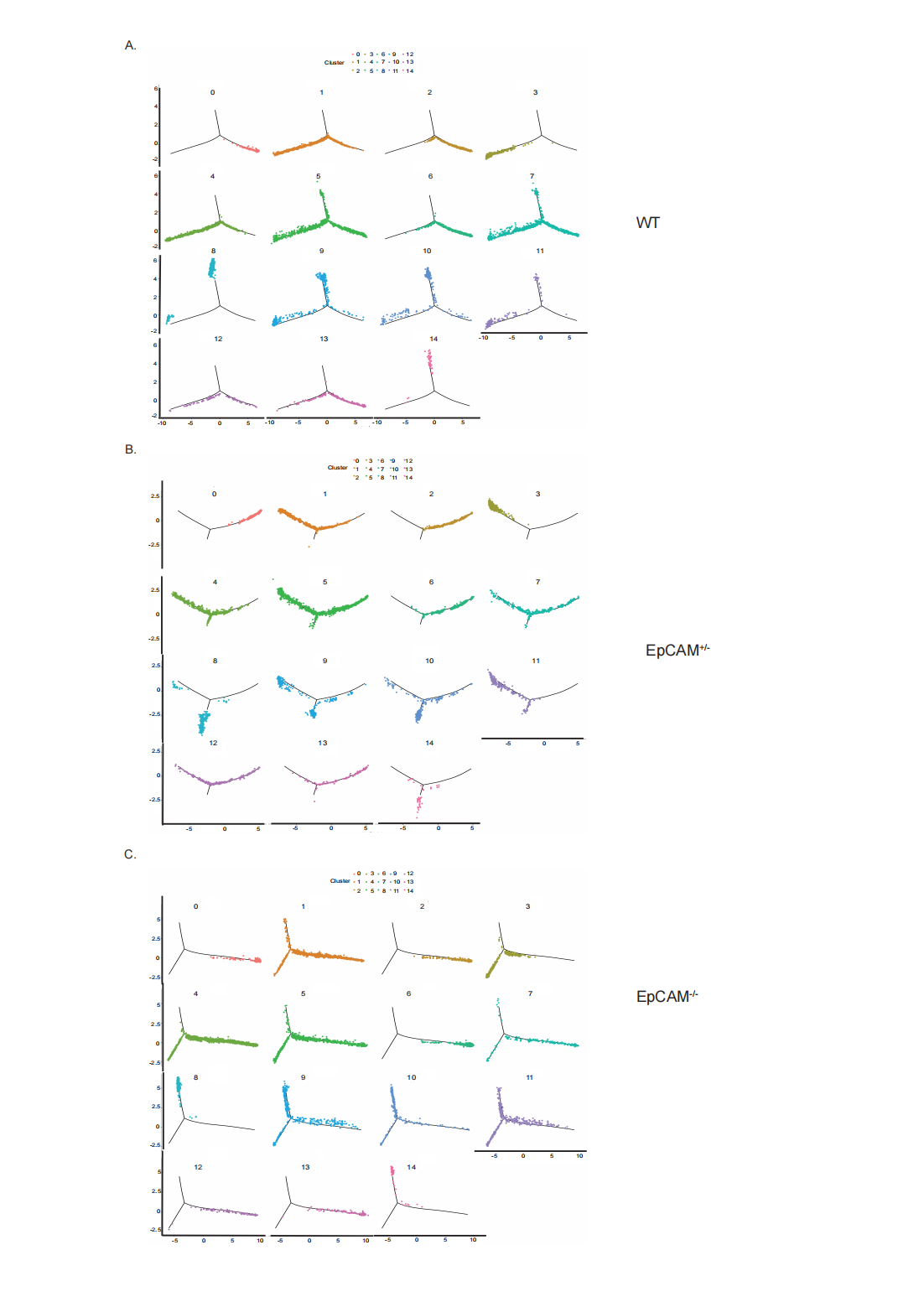


**Figure S13. The differentiation of each cluster of the intestinal epithelial cells from WT, EpCAM^+/-^ and EpCAM^-/-^ mice**

**A**. Visualization of intestinal epithelial cells in each cluster from WT E18.5 embryos along the pseudo-time trajectory from intestinal stem cells to absorptive and secretory cells in two-dimensional space. **B**. Visualization of intestinal epithelial cells in each cluster from EpCAM^+/-^ E18.5 embryos along the pseudo-time trajectory from intestinal stem cells to absorptive and secretory cells in two-dimensional space. **C**. Visualization of intestinal epithelial cells in each cluster from EpCAM^-/-^ E18.5 embryos along the pseudo-time trajectory from intestinal stem cells to absorptive and secretory cells in two-dimensional space.
