# Supplementary material for: Single-cell transcriptomics reveals EpCAM regulates the development and morphology of intestinal epithelium via controlling the EGFR pathway
Source: Genes Dis. 2026 Feb 9;13(5):102072. doi: 10.1016/j.gendis.2026.102072 (PMC13157056; doi:10.1016/j.gendis.2026.102072)
Supplement: Multimedia component 16 [file mmc16.docx]

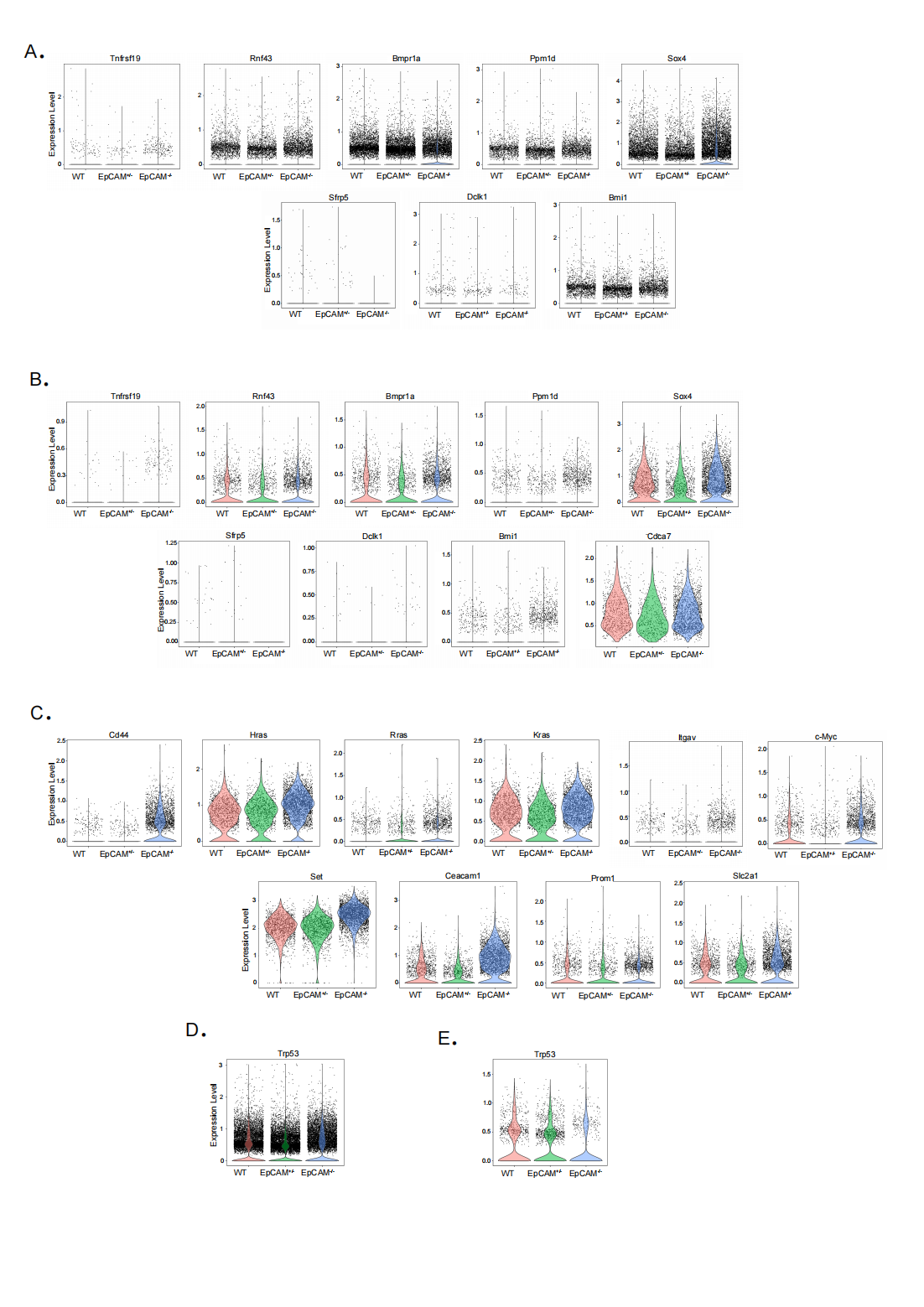


**Figure S14. Comparison of the expression of genes related to intestinal stem cells and tumorigenesis in the intestinal epithelial cells from WT, EpCAM^+/-^ and EpCAM^-/-^ mice**

**A**. Violin plots compared the expression levels of Tnfrsf19, Rnf43, Bmpr1a, Ppm1d, Sox4, Sfrp5, Dclk1 and Bmi1 in the intestinal epithelial cells from WT (Red), EpCAM^+/-^(Green) and EpCAM^-/-^ (Blue) E18.5 embryos. **B**. Violin plots compared the mRNA levels of Tnfrsf19, Rnf43, Bmpr1a, Ppm1d, Sox4, Sfrp5, Dclk1, Bmi1 and Cdca7 in the intestinal epithelial cells from Cluster 3 of WT, EpCAM^+/-^ and EpCAM^-/-^ mice. **C**. Violin plots compared the mRNA levels of Cd44, Hras, Rras, Kras, Itgav, c-Myc, Set, Ceacam1, Prom1 and Slc2a1 in the intestinal epithelial cells from Cluster 3 of WT, EpCAM^+/-^ and EpCAM^-/-^ mice. **D**. Violin plots compared the expression levels of Trp53 in the intestinal epithelial cells from WT (Red), EpCAM^+/-^(Green) and EpCAM^-/-^ (Blue) E18.5 embryos. **E**. Violin plots compared the mRNA levels of Trp53 in the intestinal epithelial cells from Cluster 6 of WT, EpCAM^+/-^ and EpCAM^-/-^

mice.
