# Supplementary material for: Single-cell transcriptomics reveals EpCAM regulates the development and morphology of intestinal epithelium via controlling the EGFR pathway
Source: Genes Dis. 2026 Feb 9;13(5):102072. doi: 10.1016/j.gendis.2026.102072 (PMC13157056; doi:10.1016/j.gendis.2026.102072)
Supplement: Multimedia component 17 [file mmc17.docx]

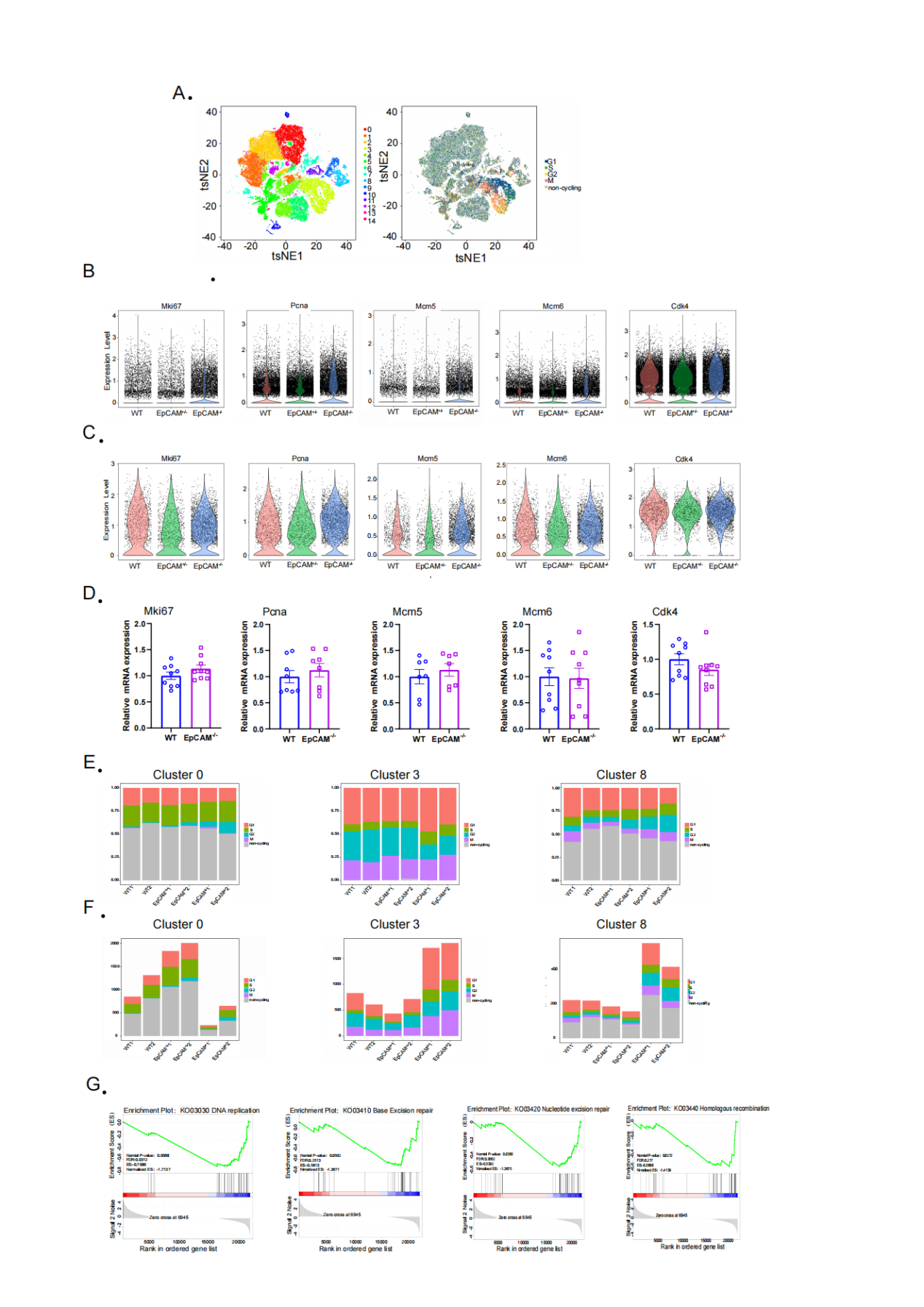


**Figure S15. Alteration of Cell Cycle of Intestinal Epithelial Cells from the EpCAM^-/-^ E18.5 Embryos**

**A**. The tSNE plots showed phases of the cell cycle of IECs from each genotype. **B**. Violin plots compared the expression levels of Mki67, Pcna, Mcm5, Mcm6 and Cdk4 in IECs from each genetype. **C**. Violin plots compared the mRNA levels of Mki67, Pcna, Mcm5, Mcm6 and Cdk4 in IECs from Cluster 3 of each genotype. **D**. The qPCR results of Mki67, Pcna, Mcm5, Mcm6 and Cdk4 from the small intestines of each group. **E**. The ratios of IECs of Cluster 0, 3 and 8 at each phase of the cell cycle from every samples. **F**. The numbers of IECs of Cluster 0, 3 and 8 at each phase of the cell cycle from every samples. **G**. GSEA showed the changes of pathways in the Cluster3.
