# Supplementary material for: Single-cell transcriptomics reveals EpCAM regulates the development and morphology of intestinal epithelium via controlling the EGFR pathway
Source: Genes Dis. 2026 Feb 9;13(5):102072. doi: 10.1016/j.gendis.2026.102072 (PMC13157056; doi:10.1016/j.gendis.2026.102072)
Supplement: Multimedia component 18 [file mmc18.docx]

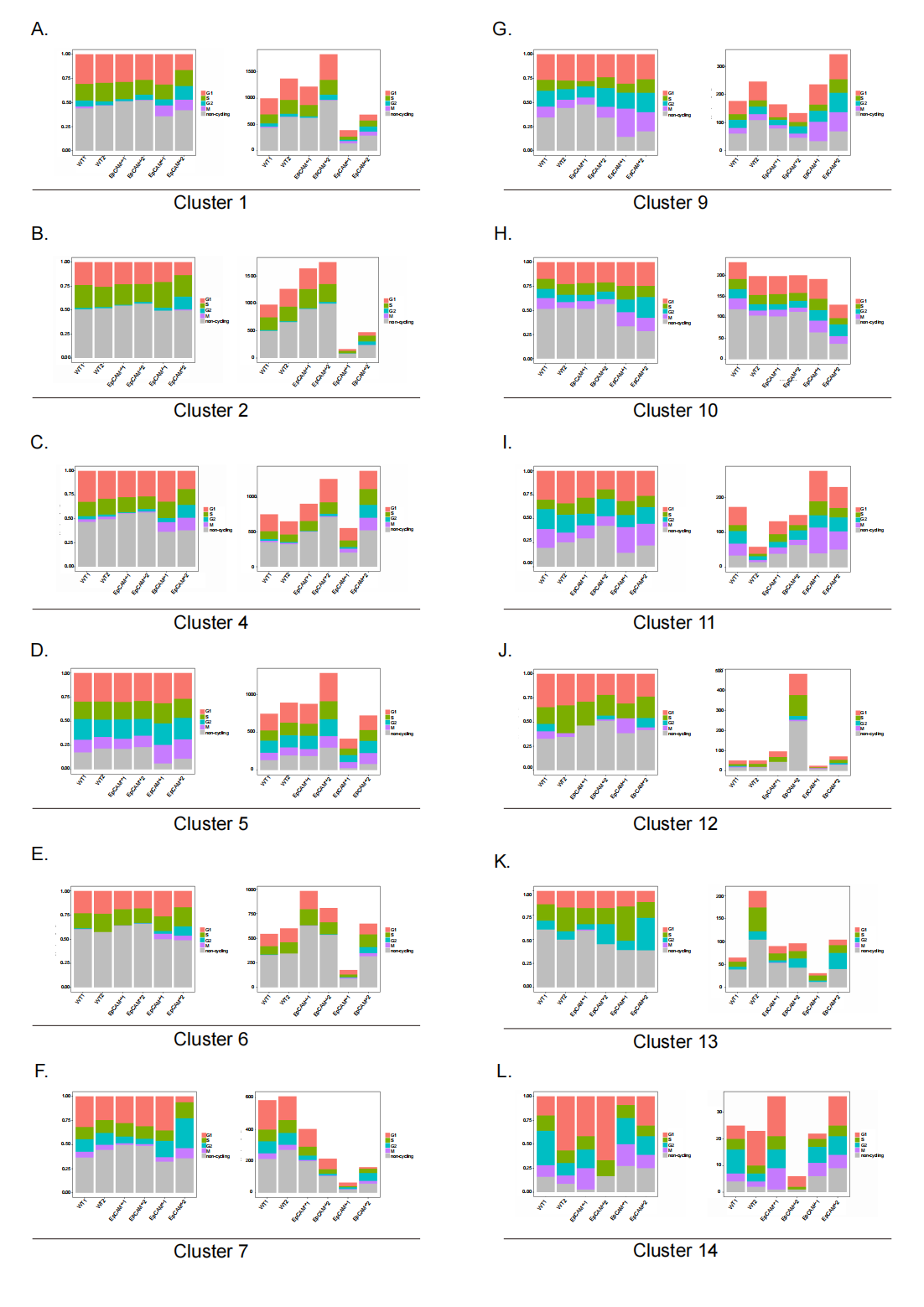


**Figure S16. Comparison of the cell cycles in the intestinal epithelial cells from WT, EpCAM^+/-^ and EpCAM^-/-^ mice**

**A**. The ratios and numbers of intestinal epithelial cells of Cluster 1 at each phase of the cell cycle from every samples of 2 WT, 2 EpCAM^+/-^, and 2 EpCAM^-/-^ E18.5 embryos. **B**. The ratios and numbers of intestinal epithelial cells of Cluster 2 at each phase of the cell cycle from every samples of 2 WT, 2 EpCAM^+/-^, and 2 EpCAM^-/-^ E18.5 embryos. **C**. The ratios and numbers of intestinal epithelial cells of Cluster 4 at each phase of the cell cycle from every samples of 2 WT, 2 EpCAM^+/-^, and 2 EpCAM^-/-^ E18.5 embryos. **D**. The ratios and numbers of intestinal epithelial cells of Cluster 5 at each phase of the cell cycle from every samples of 2 WT, 2 EpCAM^+/-^, and 2 EpCAM^-/-^ E18.5 embryos. **E**. The ratios and numbers of intestinal epithelial cells of Cluster 6 at each phase of the cell cycle from every samples of 2 WT, 2 EpCAM^+/-^, and 2 EpCAM^-/-^ E18.5 embryos. **F**. The ratios and numbers of intestinal epithelial cells of Cluster 7 at each phase of the cell cycle from every samples of 2 WT, 2 EpCAM^+/-^, and 2 EpCAM^-/-^ E18.5 embryos. **G**. The ratios and numbers of intestinal epithelial cells of Cluster 9 at each phase of the cell cycle from every samples of 2 WT, 2 EpCAM^+/-^, and 2 EpCAM^-/-^ E18.5 embryos. **H**. The ratios and numbers of intestinal epithelial cells of Cluster 10 at each phase of the cell cycle from every samples of 2 WT, 2 EpCAM^+/-^, and 2 EpCAM^-/-^ E18.5 embryos. **I**. The ratios and numbers of intestinal epithelial cells of Cluster 11 at each phase of the cell cycle from every samples of 2 WT, 2 EpCAM^+/-^, and 2 EpCAM^-/-^ E18.5 embryos. **J**. The ratios and numbers of intestinal epithelial cells of Cluster 12 at each phase of the cell cycle from every samples of 2 WT, 2 EpCAM^+/-^, and 2 EpCAM^-/-^ E18.5 embryos. **K**. The ratios and numbers of intestinal epithelial cells of Cluster 13 at each phase of the cell cycle from every samples of 2 WT, 2 EpCAM^+/-^, and 2 EpCAM^-/-^ E18.5 embryos. **L**. The ratios and numbers of intestinal epithelial cells of Cluster 14 at each phase of the cell cycle from every samples of 2 WT, 2 EpCAM^+/-^, and 2 EpCAM^-/-^ E18.5 embryos.
