# Supplementary material for: Single-cell transcriptomics reveals EpCAM regulates the development and morphology of intestinal epithelium via controlling the EGFR pathway
Source: Genes Dis. 2026 Feb 9;13(5):102072. doi: 10.1016/j.gendis.2026.102072 (PMC13157056; doi:10.1016/j.gendis.2026.102072)
Supplement: Multimedia component 19 [file mmc19.docx]

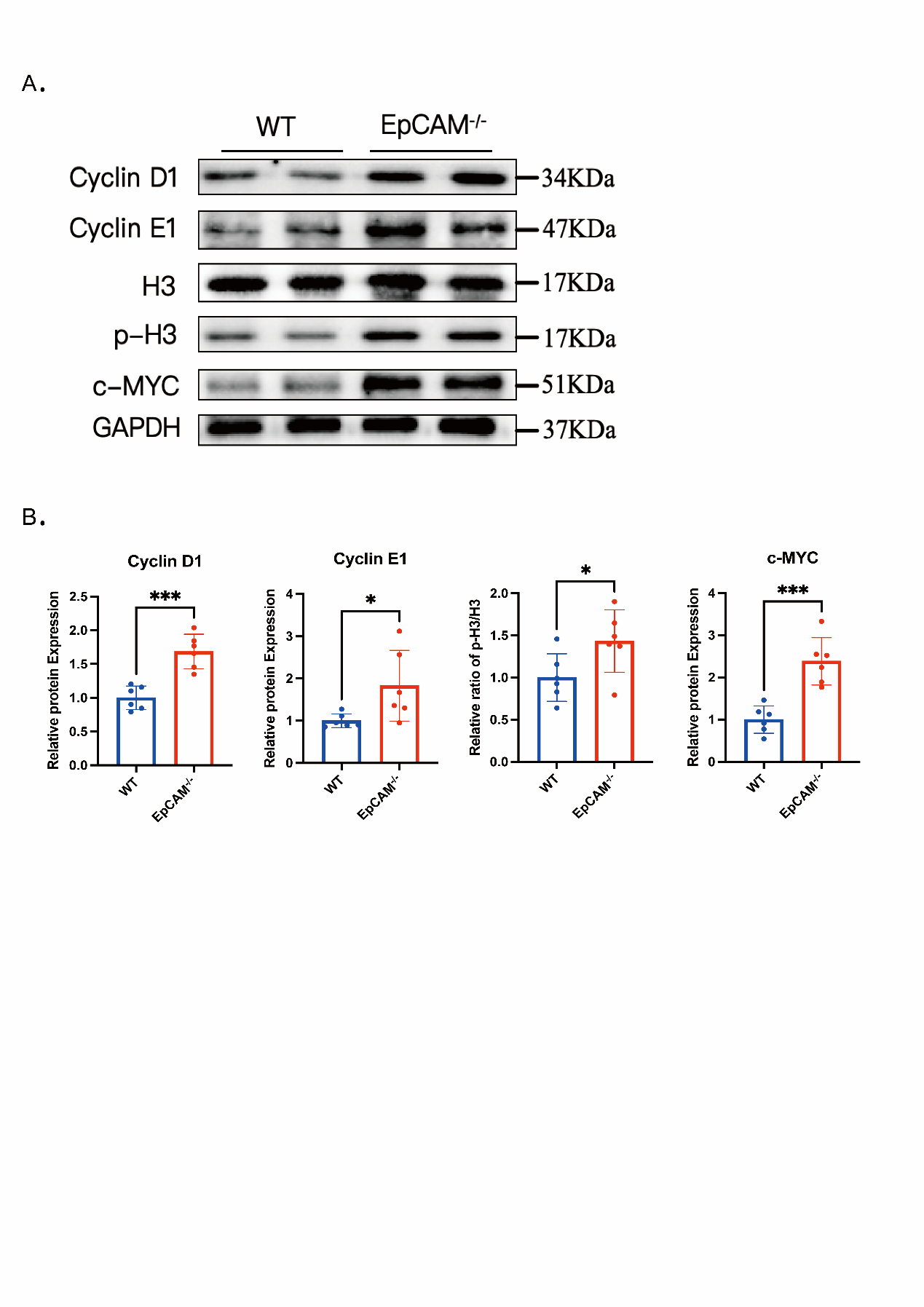


**Figure S17.** **The Deficiency of EpCAM Increased the Proliferation of Intestinal Epithelial Cells of E18.5 Embryos**

**A**. Western blot analysis of the protein levels of Cyclin D1, Cyclin E1, H3, p-H3 and c-MYC in the small intestines from WT and EpCAM^-/-^ groups of embryos at E18.5 stage. **B**. The quantification data of A. 6 mice in each group for 3 times independent experiments. H3, Histone 3; p-H3, p-Histone 3. *p<0.05, ***p<0.001.
