# Supplementary material for: Single-cell transcriptomics reveals EpCAM regulates the development and morphology of intestinal epithelium via controlling the EGFR pathway
Source: Genes Dis. 2026 Feb 9;13(5):102072. doi: 10.1016/j.gendis.2026.102072 (PMC13157056; doi:10.1016/j.gendis.2026.102072)
Supplement: Multimedia component 20 [file mmc20.docx]

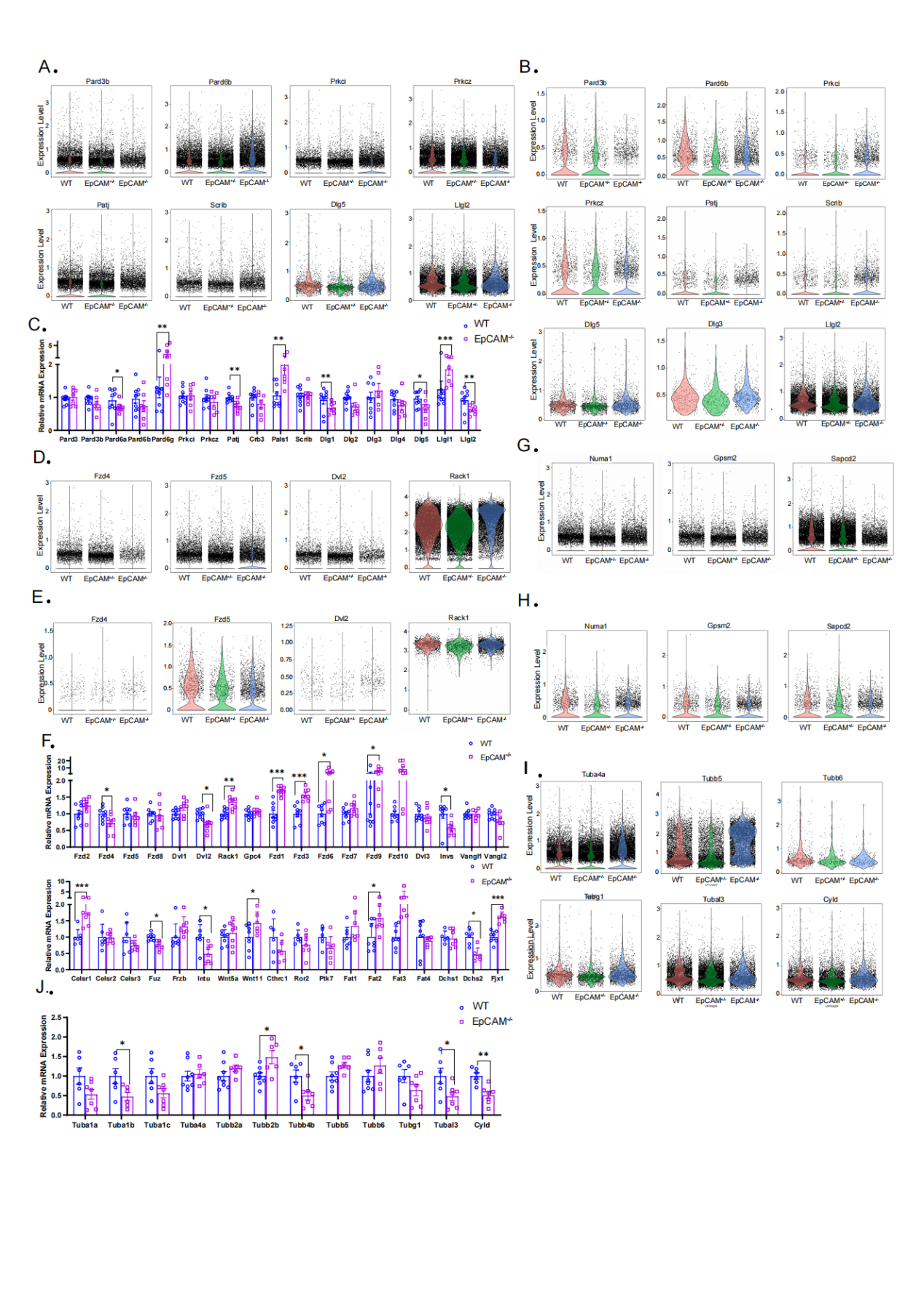


**Figure S18. Alteration of Cell Polarity and Cytoskeleton of Intestinal Epithelial Cells from the EpCAM^-/-^ E18.5 Embryos**

Violin plots compared the expression levels of Pard3b, Pard6b, Prkci, Prkcz, Patj, Scrib, Dlg5 and Llgl2 in IECs from each genotype. **B**. Violin plots compared the mRNA levels of Pard3b, Pard6b, Prkci, Prkcz, Patj, Scrib, Dlg5, Dlg3 and Llgl2 in IECs from Cluster 3 of each genotype. **C**. The qPCR results of Pard3, Pard3b, Pard6a, Pard6b, Pard6g, Prkci, Prkcz, Patj, Crb3, Pals1, Scrib, Dlg1, Dlg2, Dlg3, Dlg4, Dlg5, Llgl1 and Llgl2 from the small intestines of each group. **D**. Violin plots compared the expression levels of Fzd4, Fzd5, Dvl2 and Rack1 in IECs from each genotype. **E**. Violin plots compared the mRNA levels of Fzd4, Fzd5, Dvl2 and Rack1 in IECs from Cluster 3 of each genotype. **F**. The qPCR results of Fzd2, Fzd4, Fzd5, Fzd8, Dvl1, Dvl2, Rack1, Gpc4, Fzd1, Fzd3, Fzd6, Fzd7, Fzd9, Fzd10, Dvl3, Invs, Vangl1, Vangl2, Celsr1, Celsr2, Celsr3, Fuz, Frzb, Intu, Wnt5a, Wnt11, Cthrc1, Ror2, Ptk7, Fat1, Fat2, Fat3, Fat4, Dchs1, Dchs2 and Fjx1from the small intestines of each group. **G**. Violin plots compared the expression levels of Numa1, Gpsm2 and Sapcd2 in IECs from each genotype. **H**. Violin plots compared the mRNA levels of Numa1, Gpsm2 and Sapcd2 in IECs from Cluster 3. **I**. Violin plots compared the expression levels of Tuba4a, Tubb5, Tubb6, Tubg1, Tubal3 and Cyld in IECs from each genotype. **J**. The qPCR results of Tuba1a, Tuba1b, Tuba1c, Tuba4a, Tubb2a, Tubb2b, Tubb4b, Tubb5, Tubb6, Tubg1, Tubal3 and Cyld from the small intestines of each group. ^*^p<0.05, ^**^p<0.01, ^***^p<0.001.
