# Supplementary material for: Single-cell transcriptomics reveals EpCAM regulates the development and morphology of intestinal epithelium via controlling the EGFR pathway
Source: Genes Dis. 2026 Feb 9;13(5):102072. doi: 10.1016/j.gendis.2026.102072 (PMC13157056; doi:10.1016/j.gendis.2026.102072)
Supplement: Multimedia component 21 [file mmc21.docx]

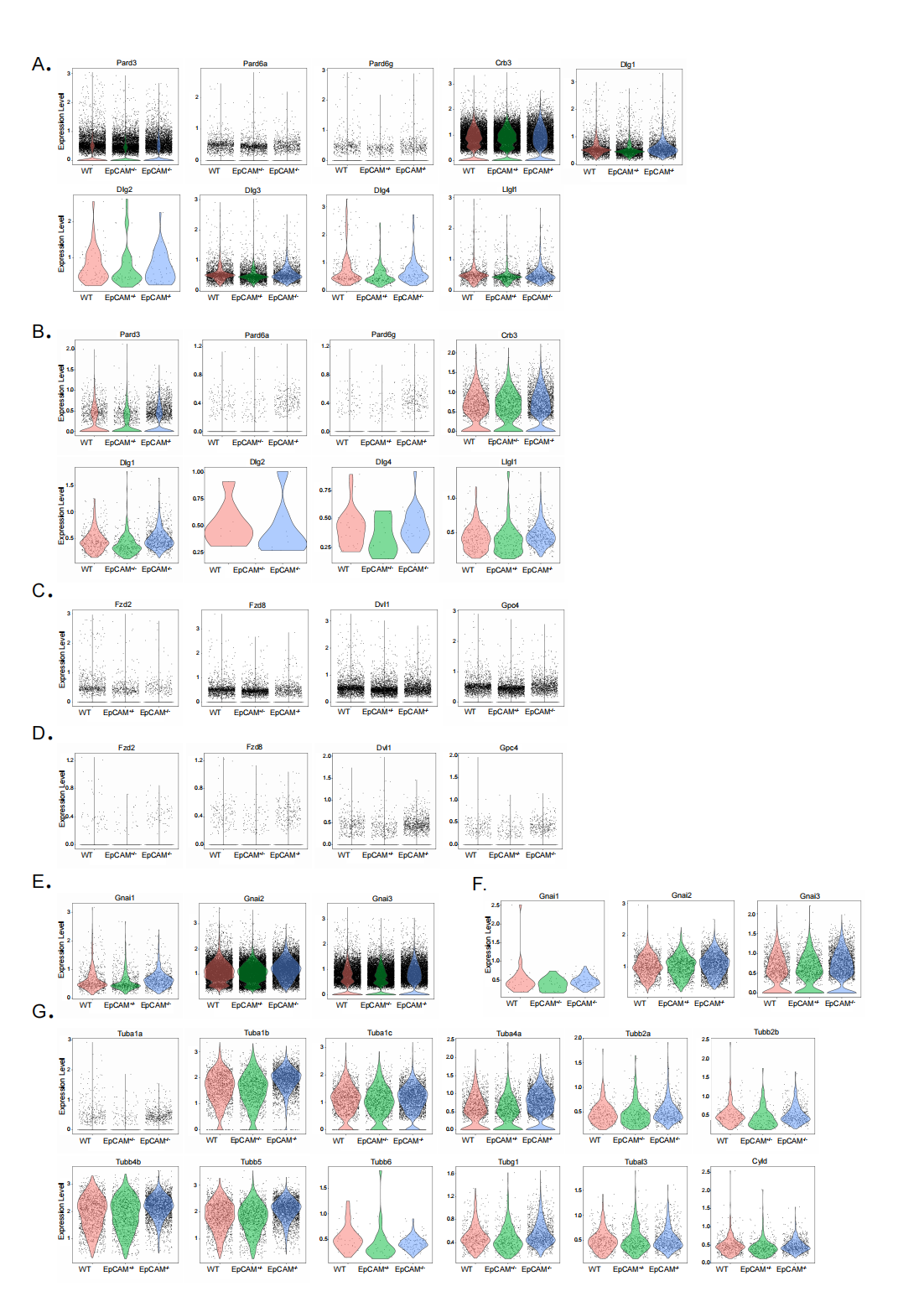


**Figure S19. The deficiency of EpCAM affected the expression of genes related to polarity and microtubules in the intestinal epithelial cells**

**A**. Violin plots compared the expression levels of Pard3, Pard6a, Pard6g, Crb3, Dlg1, Dlg2, Dlg3, Dlg4 and Llgl1 in the intestinal epithelial cells from WT (Red), EpCAM^+/-^(Green) and EpCAM^-/-^ (Blue) E18.5 embryos. **B**. Violin plots compared the mRNA levels of Pard3, Pard6a, Pard6g, Crb3, Dlg1, Dlg2, Dlg4 and Llgl1 in the intestinal epithelial cells from Cluster 3 of WT, EpCAM^+/-^ and EpCAM^-/-^ mice. **C**. Violin plots compared the expression levels of Fzd2, Fzd8, Dvl1 and Gpc4 in the intestinal epithelial cells from WT (Red), EpCAM^+/-^(Green) and EpCAM^-/-^ (Blue) E18.5 embryos. **D**. Violin plots compared the mRNA levels of Fzd2, Fzd8, Dvl1 and Gpc4 in the intestinal epithelial cells from Cluster 3 of WT, EpCAM^+/-^ and EpCAM^-/-^ mice. **E**. Violin plots compared the expression levels of Gnai1, Gnai2 and Gnai3 in the intestinal epithelial cells from WT (Red), EpCAM^+/-^(Green) and EpCAM^-/-^ (Blue) E18.5 embryos. **F**. Violin plots compared the mRNA levels of Gnai1, Gnai2 and Gnai3 in the intestinal epithelial cells from Cluster 3 of WT, EpCAM^+/-^ and EpCAM^-/-^ mice. **G**. Violin plots compared the mRNA levels of Tuba1a, Tuba1b, Tuba1c, Tuba4a, Tubb2a, Tubb2b, Tubb4b, Tubb5, Tubb6, Tubg1, Tubal3 and Cyld in the intestinal epithelial cells from Cluster 3 of WT, EpCAM^+/-^ and EpCAM^-/-^ mice.
