# Supplementary material for: Single-cell transcriptomics reveals EpCAM regulates the development and morphology of intestinal epithelium via controlling the EGFR pathway
Source: Genes Dis. 2026 Feb 9;13(5):102072. doi: 10.1016/j.gendis.2026.102072 (PMC13157056; doi:10.1016/j.gendis.2026.102072)
Supplement: Multimedia component 22 [file mmc22.docx]

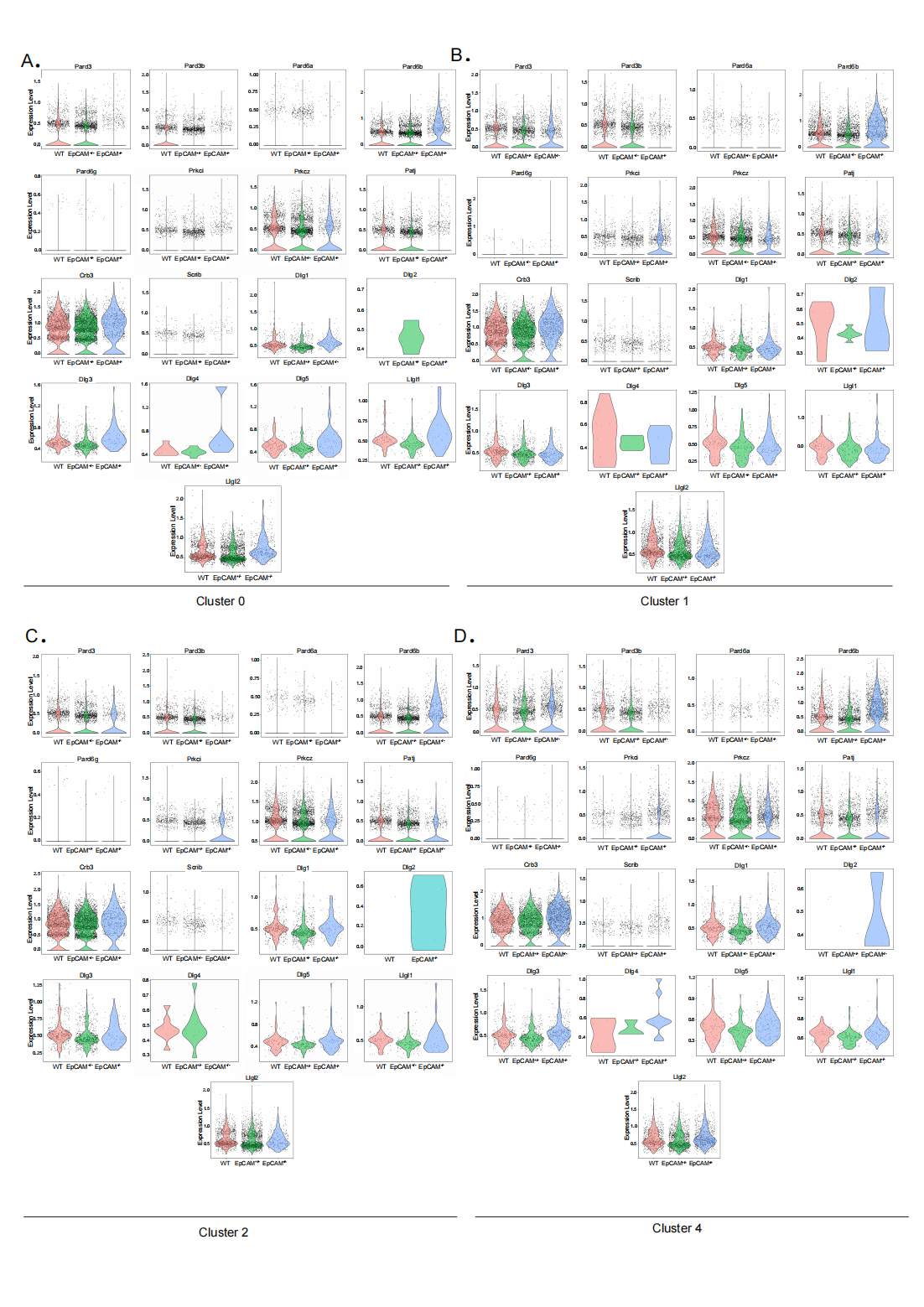


**Figure S20. Comparison of the expression of genes related to apical-basal polarity in the intestinal epithelial cells from WT, EpCAM^+/-^ and EpCAM^-/-^ mice**

**A**. Violin plots compared the mRNA levels of Pard3, Pard3b, Pard6a, Pard6b, Pard6g, Prkci, Prkcz, Patj, Crb3, Scrib, Dlg1, Dlg2, Dlg3, Dlg4, Dlg5, Llgl1 and Llgl2 in the intestinal epithelial cells from Cluster 0 of WT, EpCAM^+/-^ and EpCAM^-/-^ mice. **B**. Violin plots compared the mRNA levels of Pard3, Pard3b, Pard6a, Pard6b, Pard6g, Prkci, Prkcz, Patj, Crb3, Scrib, Dlg1, Dlg2, Dlg3, Dlg4, Dlg5, Llgl1 and Llgl2 in the intestinal epithelial cells from Cluster 1 of WT, EpCAM^+/-^ and EpCAM^-/-^ mice. **C**. Violin plots compared the mRNA levels of Pard3, Pard3b, Pard6a, Pard6b, Pard6g, Prkci, Prkcz, Patj, Crb3, Scrib, Dlg1, Dlg2, Dlg3, Dlg4, Dlg5, Llgl1 and Llgl2 in the intestinal epithelial cells from Cluster 2 of WT, EpCAM^+/-^ and EpCAM^-/-^ mice. **D**. Violin plots compared the mRNA levels of Pard3, Pard3b, Pard6a, Pard6b, Pard6g, Prkci, Prkcz, Patj, Crb3, Scrib, Dlg1, Dlg2, Dlg3, Dlg4, Dlg5, Llgl1 and Llgl2 in the intestinal epithelial cells from Cluster 4 of WT, EpCAM^+/-^ and EpCAM^-/-^ mice.
