# Supplementary material for: Single-cell transcriptomics reveals EpCAM regulates the development and morphology of intestinal epithelium via controlling the EGFR pathway
Source: Genes Dis. 2026 Feb 9;13(5):102072. doi: 10.1016/j.gendis.2026.102072 (PMC13157056; doi:10.1016/j.gendis.2026.102072)
Supplement: Multimedia component 23 [file mmc23.docx]

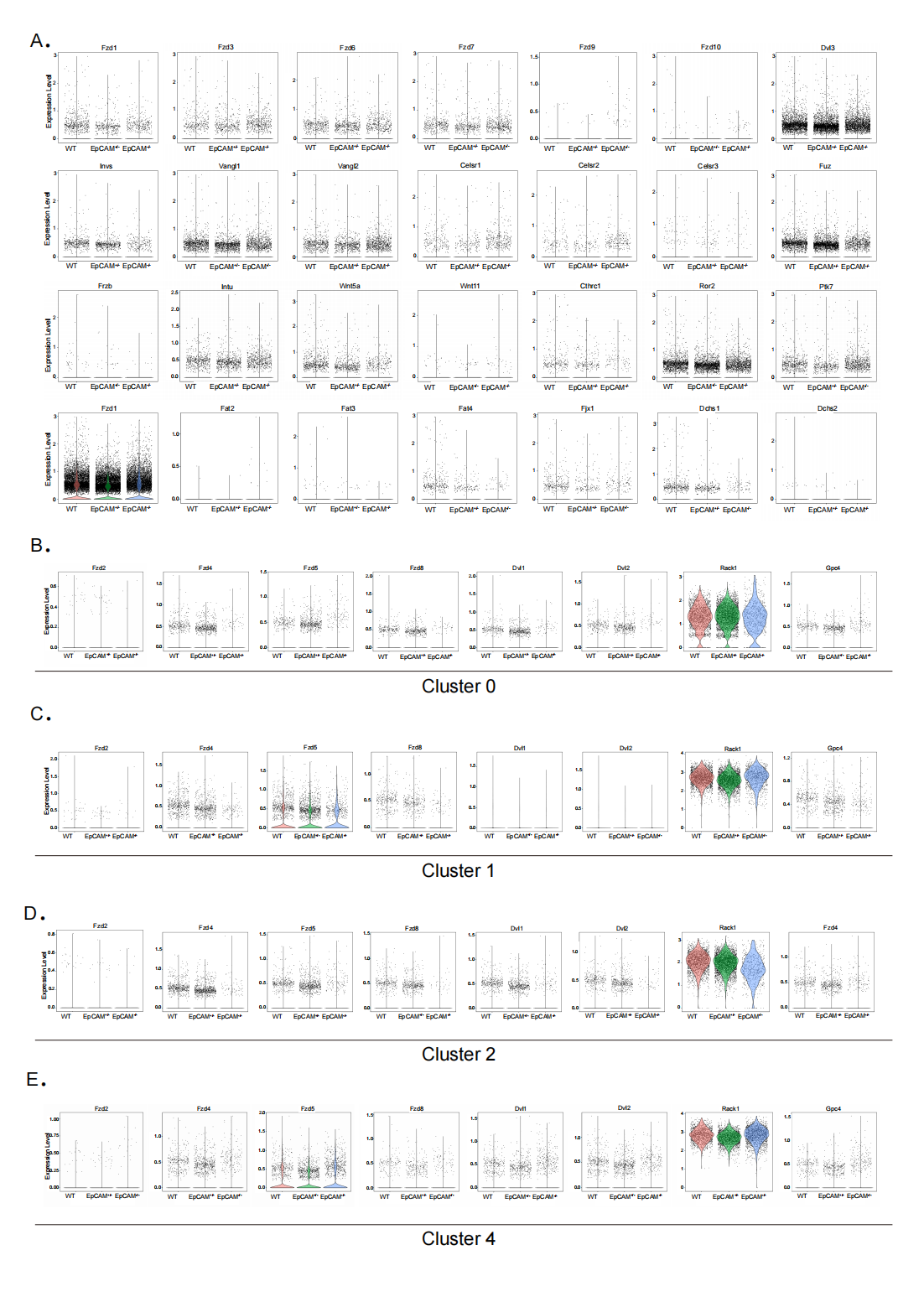


**Figure S21. Comparison of the expression of genes related to planar cell polarity in the intestinal epithelial cells from WT, EpCAM^+/-^ and EpCAM^-/-^ mice**

**A**. Violin plots compared the expression levels of Fzd1, Fzd3, Fzd6, Fzd7, Fzd9, Fzd10, Dvl3, Invs, Vangl1, Vangl2, Celsr1, Celsr2, Celsr3, Fuz, Frzb, Intu, Wnt5a, Wnt11, Cthrc1, Ror2, Ptk7, Fat1, Fat2, Fat3, Fat4, Dchs1, Dchs2 and Fjx1 in the intestinal epithelial cells from WT (Red), EpCAM^+/-^(Green) and EpCAM^-/-^ (Blue) E18.5 embryos. **B**. Violin plots compared the mRNA levels of Fzd2, Fzd4, Fzd5, Fzd8, Dvl1, Dvl2, Rack1 and Gpc4 in the intestinal epithelial cells from Cluster 0 of WT, EpCAM^+/-^ and EpCAM^-/-^ mice. **C**. Violin plots compared the mRNA levels of Fzd2, Fzd4, Fzd5, Fzd8, Dvl1, Dvl2, Rack1 and Gpc4 in the intestinal epithelial cells from Cluster 1 of WT, EpCAM^+/-^ and EpCAM^-/-^ mice. **D**. Violin plots compared the mRNA levels of Fzd2, Fzd4, Fzd5, Fzd8, Dvl1, Dvl2, Rack1 and Gpc4 in the intestinal epithelial cells from Cluster 2 of WT, EpCAM^+/-^ and EpCAM^-/-^ mice. **E**. Violin plots compared the mRNA levels of Fzd2, Fzd4, Fzd5, Fzd8, Dvl1, Dvl2, Rack1 and Gpc4 in the intestinal epithelial cells from Cluster 4 of WT, EpCAM^+/-^ and EpCAM^-/-^ mice.
