# Supplementary material for: Single-cell transcriptomics reveals EpCAM regulates the development and morphology of intestinal epithelium via controlling the EGFR pathway
Source: Genes Dis. 2026 Feb 9;13(5):102072. doi: 10.1016/j.gendis.2026.102072 (PMC13157056; doi:10.1016/j.gendis.2026.102072)
Supplement: Multimedia component 24 [file mmc24.docx]

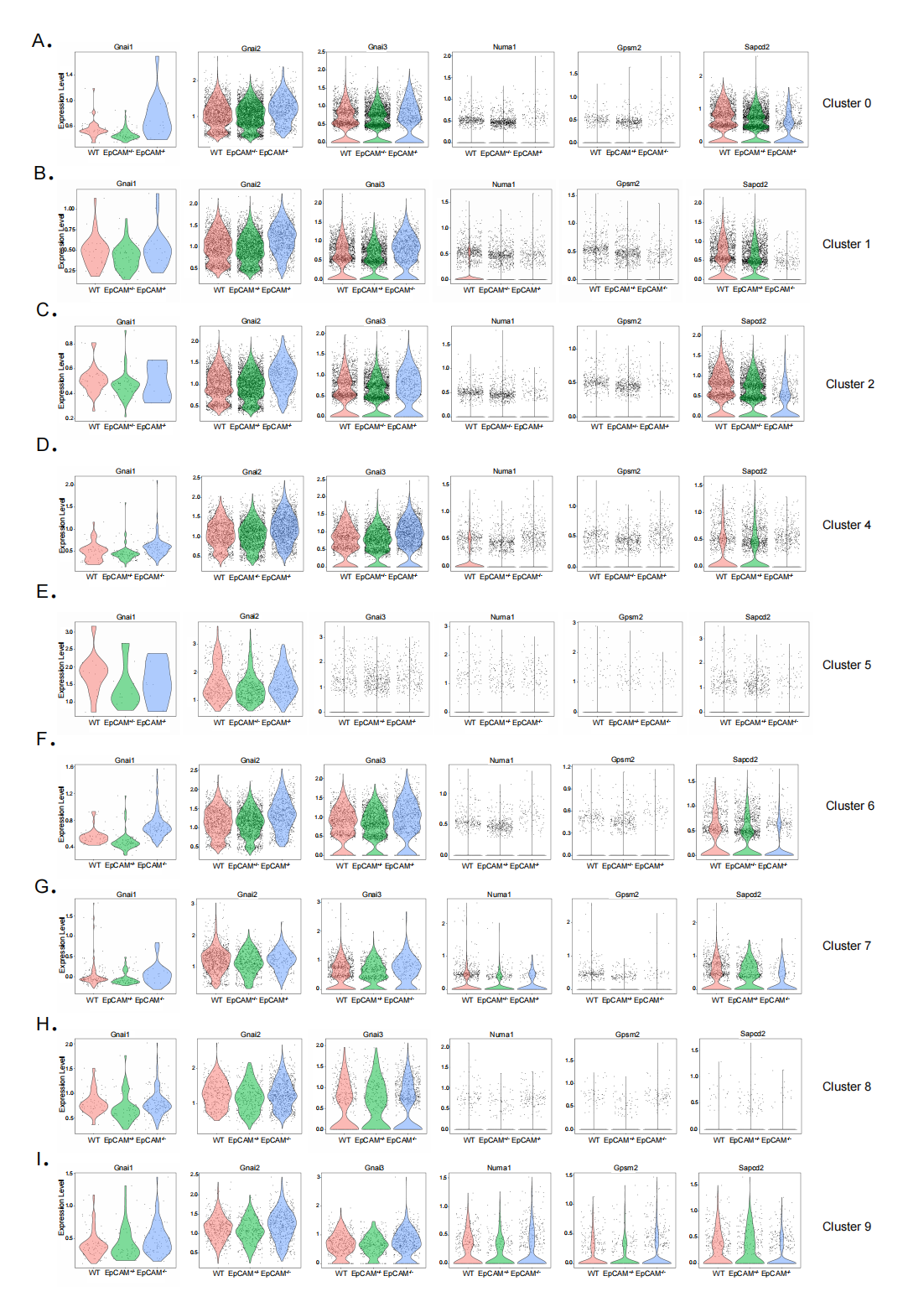


**Figure S22. Comparison of the expression of genes related to Gαi/LGN/NuMA complex in the intestinal epithelial cells from WT, EpCAM^+/-^ and EpCAM^-/-^ mice**

**A**. Violin plots compared the mRNA levels of Gnai1, Gnai2, Gnai3, Numa1, Gpsm2 and Sapcd2 in the intestinal epithelial cells from Cluster 0 of WT, EpCAM^+/-^ and EpCAM^-/-^ mice. **B**. Violin plots compared the mRNA levels of Gnai1, Gnai2, Gnai3, Numa1, Gpsm2 and Sapcd2 in the intestinal epithelial cells from Cluster 1 of WT, EpCAM^+/-^ and EpCAM^-/-^ mice. **C**. Violin plots compared the mRNA levels of Gnai1, Gnai2, Gnai3, Numa1, Gpsm2 and Sapcd2 in the intestinal epithelial cells from Cluster 2 of WT, EpCAM^+/-^ and EpCAM^-/-^ mice. **D**. Violin plots compared the mRNA levels of Gnai1, Gnai2, Gnai3, Numa1, Gpsm2 and Sapcd2 in the intestinal epithelial cells from Cluster 4 of WT, EpCAM^+/-^ and EpCAM^-/-^ mice. **E**. Violin plots compared the mRNA levels of Gnai1, Gnai2, Gnai3, Numa1, Gpsm2 and Sapcd2 in the intestinal epithelial cells from Cluster 5 of WT, EpCAM^+/-^ and EpCAM^-/-^ mice. **F**. Violin plots compared the mRNA levels of Gnai1, Gnai2, Gnai3, Numa1, Gpsm2 and Sapcd2 in the intestinal epithelial cells from Cluster 6 of WT, EpCAM^+/-^ and EpCAM^-/-^ mice. **G**. Violin plots compared the mRNA levels of Gnai1, Gnai2, Gnai3, Numa1, Gpsm2 and Sapcd2 in the intestinal epithelial cells from Cluster 7 of WT, EpCAM^+/-^ and EpCAM^-/-^ mice. **H**. Violin plots compared the mRNA levels of Gnai1, Gnai2, Gnai3, Numa1, Gpsm2 and Sapcd2 in the intestinal epithelial cells from Cluster 8 of WT, EpCAM^+/-^ and EpCAM^-/-^ mice. **I**. Violin plots compared the mRNA levels of Gnai1, Gnai2, Gnai3, Numa1, Gpsm2 and Sapcd2 in the intestinal epithelial cells from Cluster 9 of WT, EpCAM^+/-^ and EpCAM^-/-^ mice.
