# Supplementary material for: Single-cell transcriptomics reveals EpCAM regulates the development and morphology of intestinal epithelium via controlling the EGFR pathway
Source: Genes Dis. 2026 Feb 9;13(5):102072. doi: 10.1016/j.gendis.2026.102072 (PMC13157056; doi:10.1016/j.gendis.2026.102072)
Supplement: Multimedia component 25 [file mmc25.docx]

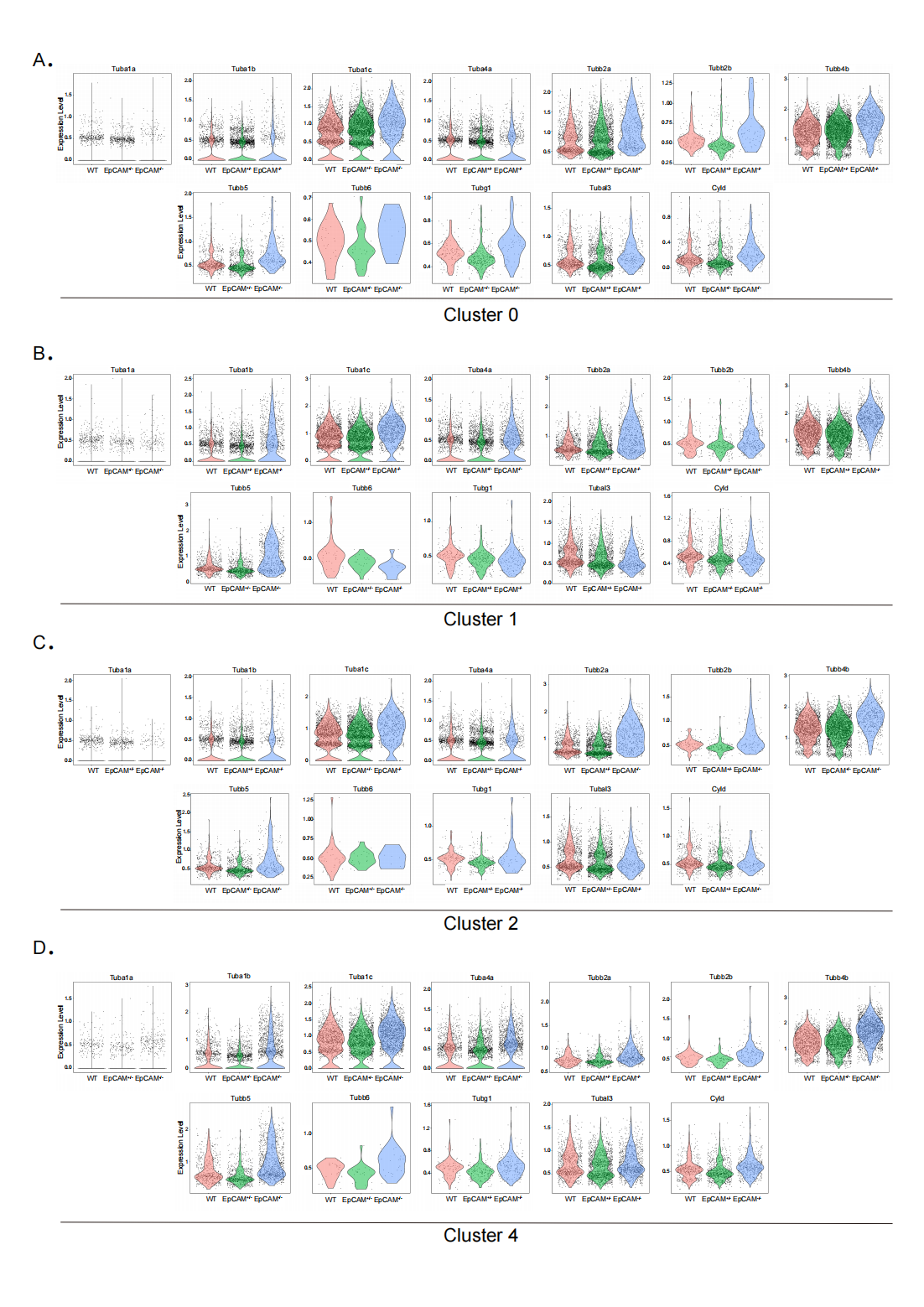


**Figure S23. Comparison of the expression of genes encoding tubulins in the intestinal epithelial cells from WT, EpCAM^+/-^ and EpCAM^-/-^ mice**

**A**. Violin plots compared the mRNA levels of Tuba1a, Tuba1b, Tuba1c, Tuba4a, Tubb2a, Tubb2b, Tubb4b, Tubb5, Tubb6, Tubg1, Tubal3 and Cyld in the intestinal epithelial cells from Cluster 0 of WT, EpCAM^+/-^ and EpCAM^-/-^ mice. **B**. Violin plots compared the mRNA levels of Tuba1a, Tuba1b, Tuba1c, Tuba4a, Tubb2a, Tubb2b, Tubb4b, Tubb5, Tubb6, Tubg1, Tubal3 and Cyld in the intestinal epithelial cells from Cluster 1 of WT, EpCAM^+/-^ and EpCAM^-/-^ mice. **C**. Violin plots compared the mRNA levels of Tuba1a, Tuba1b, Tuba1c, Tuba4a, Tubb2a, Tubb2b, Tubb4b, Tubb5, Tubb6, Tubg1, Tubal3 and Cyld in the intestinal epithelial cells from Cluster 2 of WT, EpCAM^+/-^ and EpCAM^-/-^ mice. **D** Violin plots compared the mRNA levels of Tuba1a, Tuba1b, Tuba1c, Tuba4a, Tubb2a, Tubb2b, Tubb4b, Tubb5, Tubb6, Tubg1, Tubal3 and Cyld in the intestinal epithelial cells from Cluster 4 of WT, EpCAM^+/-^ and EpCAM^-/-^ mice.
