# Supplementary material for: Single-cell transcriptomics reveals EpCAM regulates the development and morphology of intestinal epithelium via controlling the EGFR pathway
Source: Genes Dis. 2026 Feb 9;13(5):102072. doi: 10.1016/j.gendis.2026.102072 (PMC13157056; doi:10.1016/j.gendis.2026.102072)
Supplement: Multimedia component 26 [file mmc26.docx]

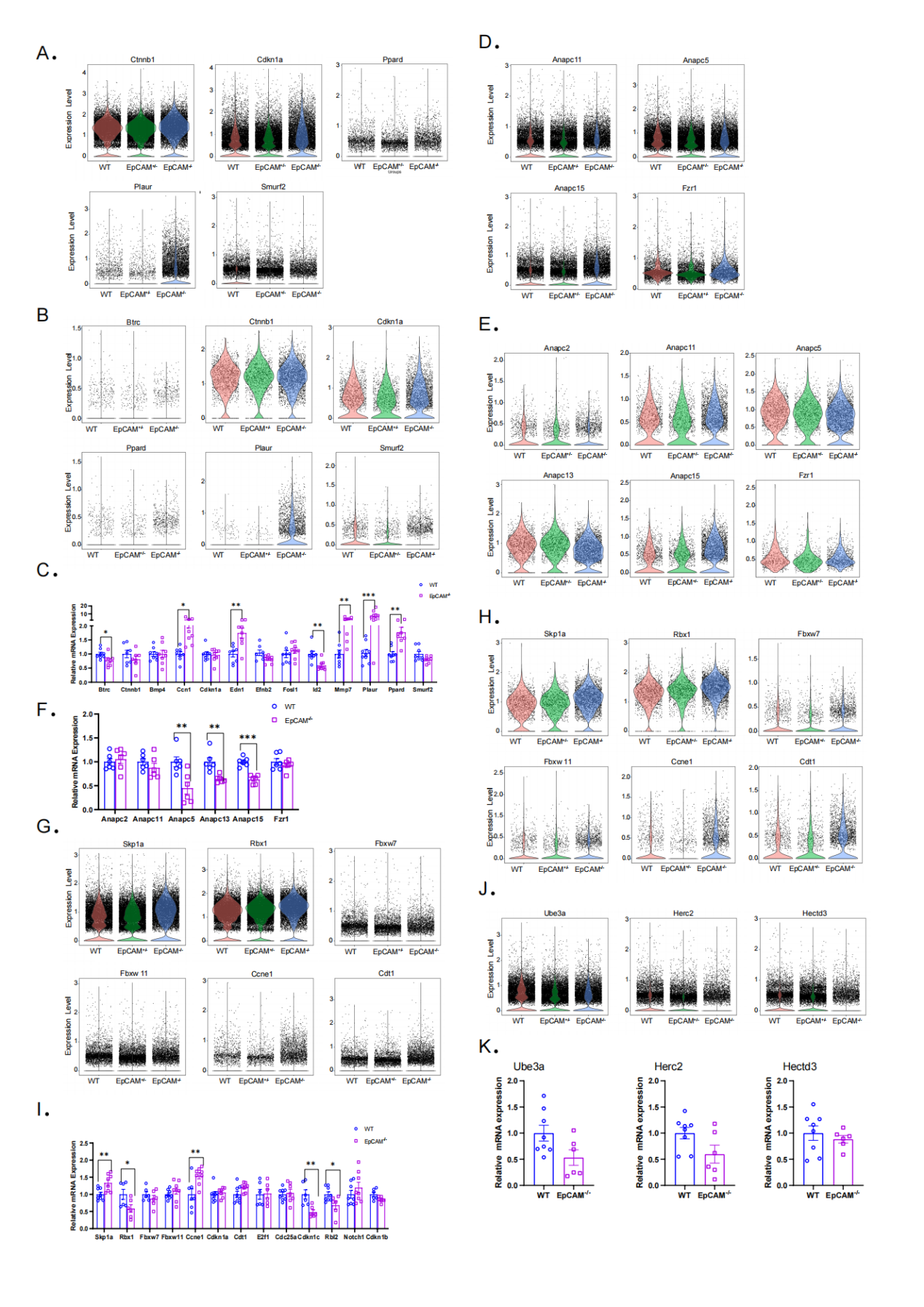


**Figure S24. The Decrease of the Expression of Genes encoding E3-Ubiquitin Ligases of the Intestinal Epithelial Cells from the EpCAM^-/-^ E18.5 Embryos**

**A**. Violin plots compared the expression levels of Ctnnb1, Cdkn1a Ppard, Plaur and Smurf2 in IECs from each genotype. **B**. Violin plots compared the mRNA levels of Btrc, Ctnnb1, Cdkn1a Ppard, Plaur and Smurf2 in IECs from Cluster 3. **C**. The qPCR results of Btrc, Ctnnb1, Bmp4, Ccn1, Cdkn1a, Edn1, Efnb2, Fosl1, Id2, Mmp7, Plaur, Ppard and Smurf2 from the small intestines of each group. **D**. Violin plots compared the expression levels of Anapc11, Anapc5, Anapc15 and Fzr1 in IECs from each genotype. **E**. Violin plots compared the mRNA levels of Anapc2, Anapc11, Anapc5, Anapc13, Anapc15 and Fzr1 in IECs from Cluster 3. **F**. The qPCR results of Anapc2, Anapc11, Anapc5, Anapc13, Anapc15 and Fzr1 from the small intestines of each group. **G**. Violin plots compared the expression levels of Skp1a, Rbx1, Fbxw7, Fbxw11, Ccne1 and Cdt1 in IECs from each genotype. **H**. Violin plots compared the mRNA levels of Skp1a, Rbx1, Fbxw7, Fbxw11, Ccne1 and Cdt1 in IECs from Cluster 3. **I**. The qPCR results of Skp1a, Rbx1, Fbxw7, Fbxw11, Ccne1, Cdkn1a, Cdt1, E2f1, Cdc25a, Cdkn1c, Rbl2, Notch1 and Cdkn1b from the small intestines of each group. **J**. Violin plots compared the expression levels of Ube3a, Herc2 and Hectd3 in IECs from each genotype. **K**. The qPCR results of Ube3a, Herc2 and Hectd3 from the small intestines of each group. ^*^p<0.05, ^**^p<0.01, ^***^p<0.001.
