# Supplementary material for: Single-cell transcriptomics reveals EpCAM regulates the development and morphology of intestinal epithelium via controlling the EGFR pathway
Source: Genes Dis. 2026 Feb 9;13(5):102072. doi: 10.1016/j.gendis.2026.102072 (PMC13157056; doi:10.1016/j.gendis.2026.102072)
Supplement: Multimedia component 28 [file mmc28.docx]

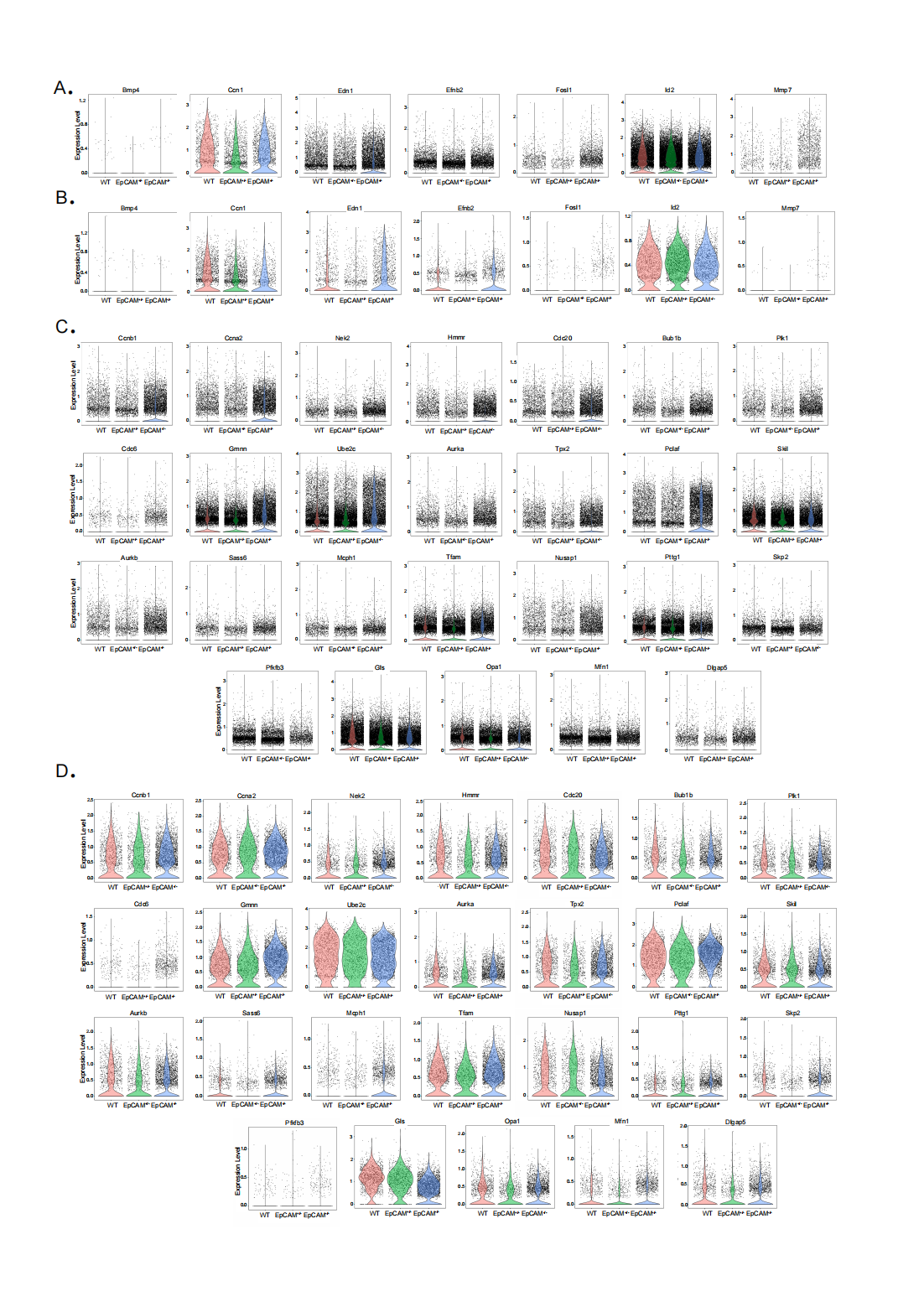


**Figure S26. Comparison the expression of genes related to E3 ubiquitin ligases and target genes of them in the intestines from E18.5 embryos of WT, EpCAM^+/-^ and EpCAM^-/-^ respectively**

**A**. Violin plots compared the expression levels of Bmp4, Ccn1, Edn1, Efnb2, Fosl1, Id2 and Mmp7 in the intestinal epithelial cells from WT (Red), EpCAM^+/-^(Green) and EpCAM^-/-^ (Blue) E18.5 embryos. **B**. Violin plots compared the mRNA levels of Bmp4, Ccn1, Edn1, Efnb2, Fosl1, Id2 and Mmp7 in the intestinal epithelial cells from Cluster 3 of WT, EpCAM^+/-^ and EpCAM^-/-^ mice. **C**. Violin plots compared the expression levels of Ccnb1, Ccna2, Nek2, Hmmr, Cdc20, Bub1b, Plk1, Cdc6, Gmnn, Ube2c, Aurka, Tpx2, Pclaf, Skil, Aurkb, Sass6, Mcph1, Tfam, Nusap1, Pttg1, Skp2, Pfkfb3, Gls, Opa1, Mfn1 and Dlgap5 in the intestinal epithelial cells from WT (Red), EpCAM^+/-^(Green) and EpCAM^-/-^ (Blue) E18.5 embryos. **D**. Violin plots compared the mRNA levels of Ccnb1, Ccna2, Nek2, Hmmr, Cdc20, Bub1b, Plk1, Cdc6, Gmnn, Ube2c, Aurka, Tpx2, Pclaf, Skil, Aurkb, Sass6, Mcph1, Tfam, Nusap1, Pttg1, Skp2, Pfkfb3, Gls, Opa1, Mfn1 and Dlgap5 in the intestinal epithelial cells from Cluster 3 of WT, EpCAM^+/-^ and EpCAM^-/-^ mice.
