# Supplementary material for: Single-cell transcriptomics reveals EpCAM regulates the development and morphology of intestinal epithelium via controlling the EGFR pathway
Source: Genes Dis. 2026 Feb 9;13(5):102072. doi: 10.1016/j.gendis.2026.102072 (PMC13157056; doi:10.1016/j.gendis.2026.102072)
Supplement: Multimedia component 29 [file mmc29.docx]

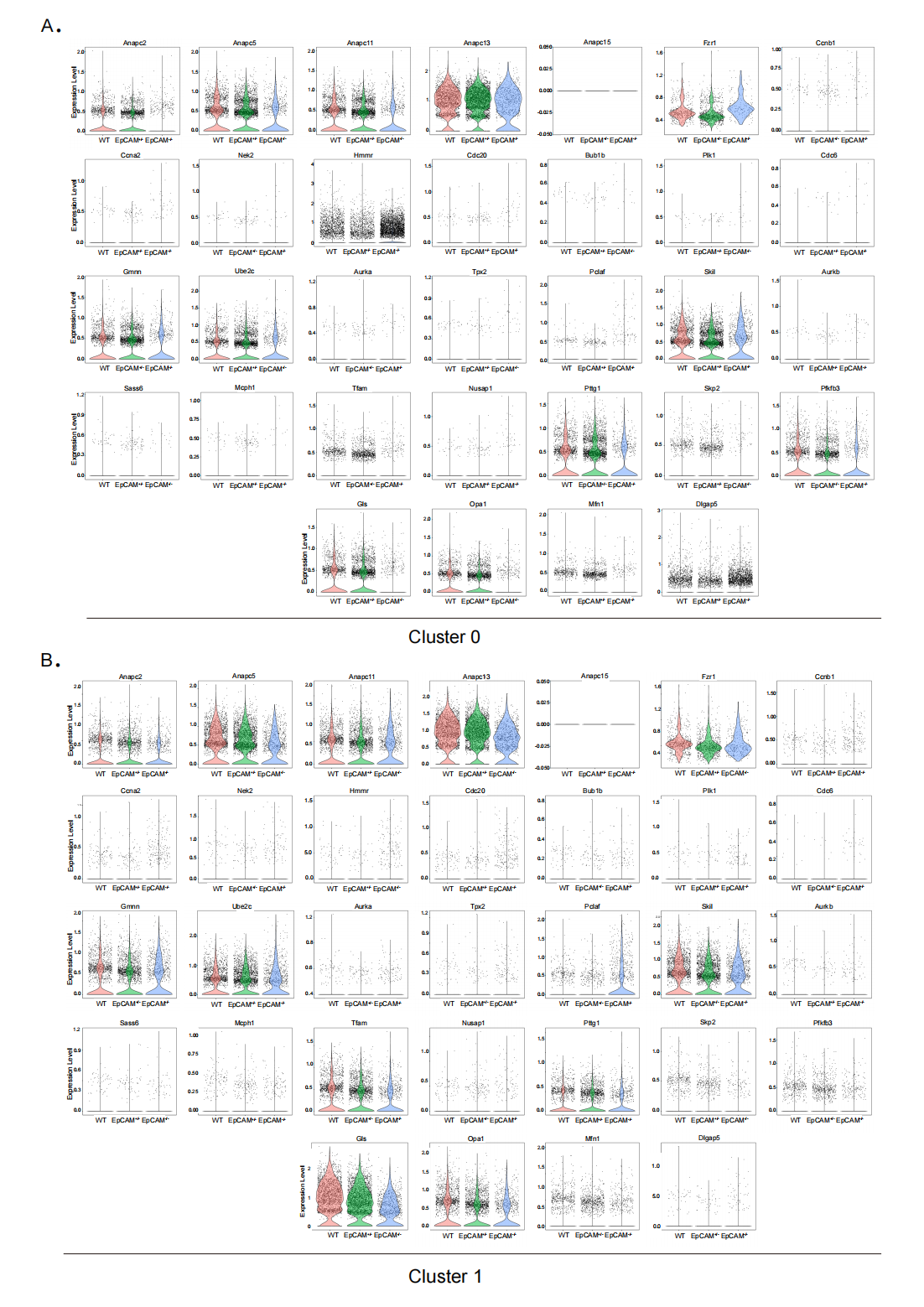


**Figure S27. Comparison of the expression of genes related to APC/C and the targets of it in the intestinal epithelial cells from WT, EpCAM^+/-^ and EpCAM^-/-^ mice**

**A**. Violin plots compared the mRNA levels of Anapc2, Anapc5, Anapc11, Anapc13, Anapc15, Fzr1, Ccnb1, Ccna2, Nek2, Hmmr, Cdc20, Bub1b, Plk1, Cdc6, Gmnn, Ube2c, Aurka, Tpx2, Pclaf, Skil, Aurkb, Sass6, Mcph1, Tfam, Nusap1, Pttg1, Skp2, Pfkfb3, Gls, Opa1, Mfn1 and Dlgap5 in the intestinal epithelial cells from Cluster 0 of WT, EpCAM^+/-^ and EpCAM^-/-^ mice. **B**. Violin plots compared the mRNA levels of Anapc2, Anapc5, Anapc11, Anapc13, Anapc15, Fzr1, Ccnb1, Ccna2, Nek2, Hmmr, Cdc20, Bub1b, Plk1, Cdc6, Gmnn, Ube2c, Aurka, Tpx2, Pclaf, Skil, Aurkb, Sass6, Mcph1, Tfam, Nusap1, Pttg1, Skp2, Pfkfb3, Gls, Opa1, Mfn1 and Dlgap5 in the intestinal epithelial cells from Cluster 1 of WT, EpCAM^+/-^ and EpCAM^-/-^ mice.
