# Supplementary material for: Single-cell transcriptomics reveals EpCAM regulates the development and morphology of intestinal epithelium via controlling the EGFR pathway
Source: Genes Dis. 2026 Feb 9;13(5):102072. doi: 10.1016/j.gendis.2026.102072 (PMC13157056; doi:10.1016/j.gendis.2026.102072)
Supplement: Multimedia component 30 [file mmc30.docx]

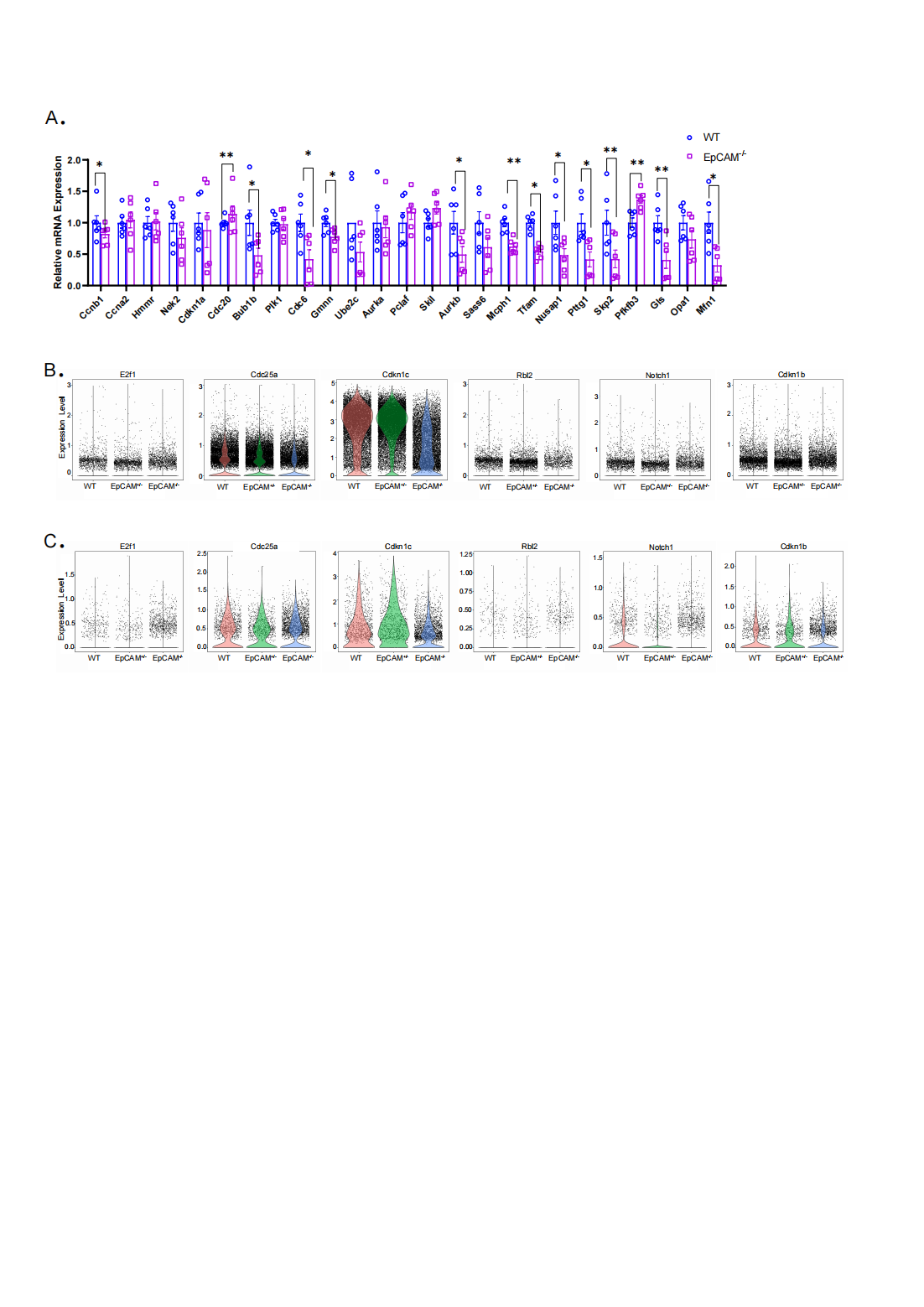


**Figure S28. Comparison of the expression of genes encoding the targeted proteins of APC/C and SCF in the intestinal epithelial cells from WT, EpCAM^+/-^ and EpCAM^-/-^ mice**

**A**. The qPCR results of Ccnb1, Ccna2, Hmmr, Nek2, Cdkn1a, Cdc20, Bub1b, Plk1, Cdc6, Gmnn, Ube2c, Aurka, Pclaf, Skil, Aurkb, Sass6, Mcph1, Tfam, Nusap1, Pttg1, Skp2, Pfkfb3, Gls, Opa1 and Mfn1 from the small intestines of WT and EpCAM^-/-^ embryos at E18.5 stage. **B**. Violin plots compared the expression levels of E2f1, Cdc25a, Cdkn1c, Rbl2, Notch1 and Cdkn1b in the intestinal epithelial cells from WT (Red), EpCAM^+/-^(Green) and EpCAM^-/-^ (Blue) E18.5 embryos. **C**. Violin plots compared the mRNA levels of E2f1, Cdc25a, Cdkn1c, Rbl2, Notch1 and Cdkn1b in the intestinal epithelial cells from Cluster 3 of WT, EpCAM^+/-^ and EpCAM^-/-^ mice. ^*^p<0.05, ^**^p<0.01.
