# Supplementary material for: Single-cell transcriptomics reveals EpCAM regulates the development and morphology of intestinal epithelium via controlling the EGFR pathway
Source: Genes Dis. 2026 Feb 9;13(5):102072. doi: 10.1016/j.gendis.2026.102072 (PMC13157056; doi:10.1016/j.gendis.2026.102072)
Supplement: Multimedia component 31 [file mmc31.docx]

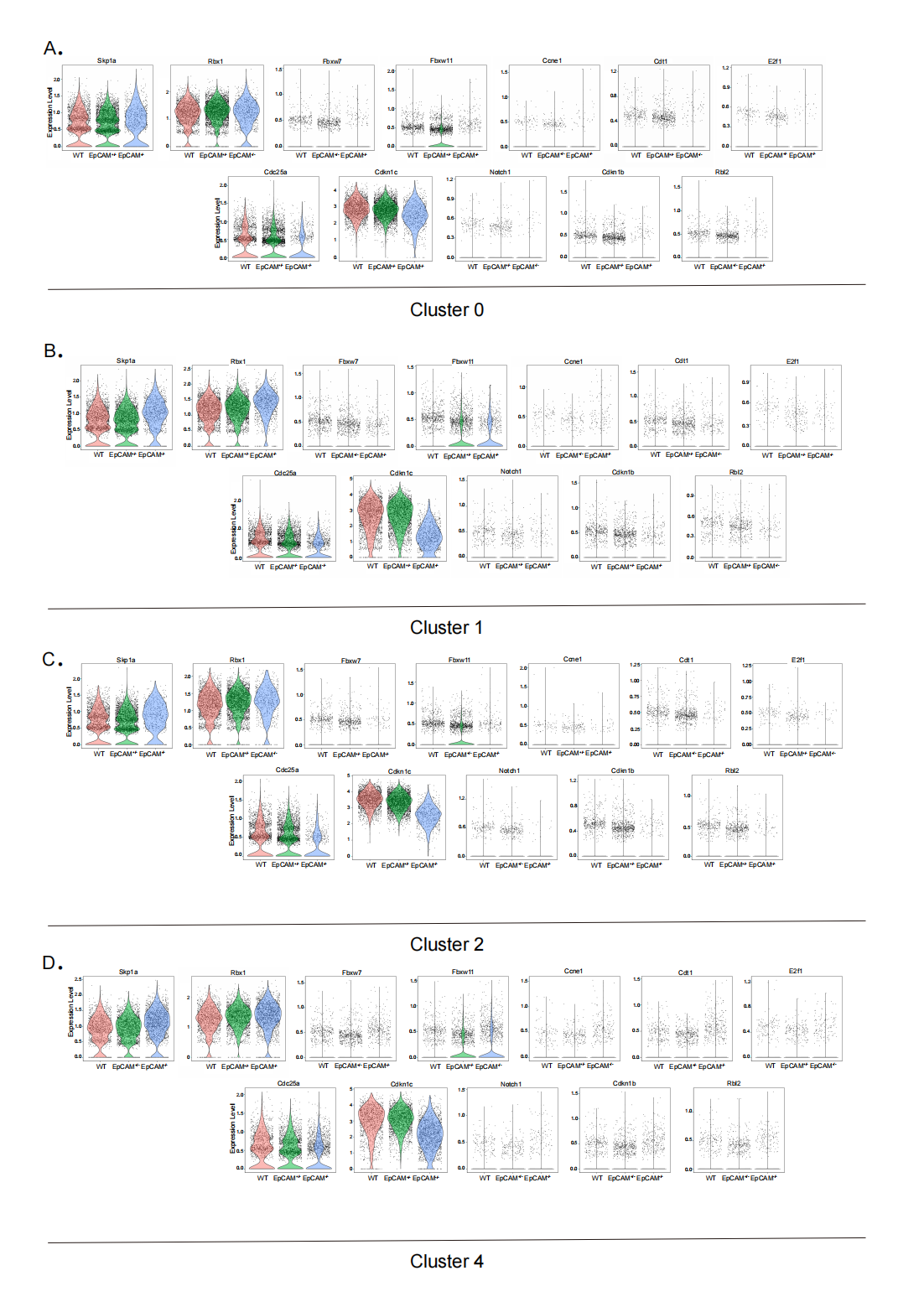


**Figure S29. Comparison of the expression of genes related to SCF and the targets of it in the intestinal epithelial cells from WT, EpCAM^+/-^ and EpCAM^-/-^ mice**

**A**. Violin plots compared the mRNA levels of Skp1a, Rbx1, Fbxw7, Fbxw11, Ccne1, Cdt1, E2f1, Cdc25a, Cdkn1c, Notch1, Cdkn1b and Rbl2 in the intestinal epithelial cells from Cluster 0 of WT, EpCAM^+/-^ and EpCAM^-/-^ mice. **B**. Violin plots compared the mRNA levels of Skp1a, Rbx1, Fbxw7, Fbxw11, Ccne1, Cdt1, E2f1, Cdc25a, Cdkn1c, Notch1, Cdkn1b and Rbl2 in the intestinal epithelial cells from Cluster 1 of WT, EpCAM^+/-^ and EpCAM^-/-^ mice. **C**. Violin plots compared the mRNA levels of Skp1a, Rbx1, Fbxw7, Fbxw11, Ccne1, Cdt1, E2f1, Cdc25a, Cdkn1c, Notch1, Cdkn1b and Rbl2 in the intestinal epithelial cells from Cluster 2 of WT, EpCAM^+/-^ and EpCAM^-/-^ mice. **D** Violin plots compared the mRNA levels of Skp1a, Rbx1, Fbxw7, Fbxw11, Ccne1, Cdt1, E2f1, Cdc25a, Cdkn1c, Notch1, Cdkn1b and Rbl2 in the intestinal epithelial cells from Cluster 4 of WT, EpCAM^+/-^ and EpCAM^-/-^ mice.
