# Supplementary material for: Single-cell transcriptomics reveals EpCAM regulates the development and morphology of intestinal epithelium via controlling the EGFR pathway
Source: Genes Dis. 2026 Feb 9;13(5):102072. doi: 10.1016/j.gendis.2026.102072 (PMC13157056; doi:10.1016/j.gendis.2026.102072)
Supplement: Multimedia component 32 [file mmc32.docx]

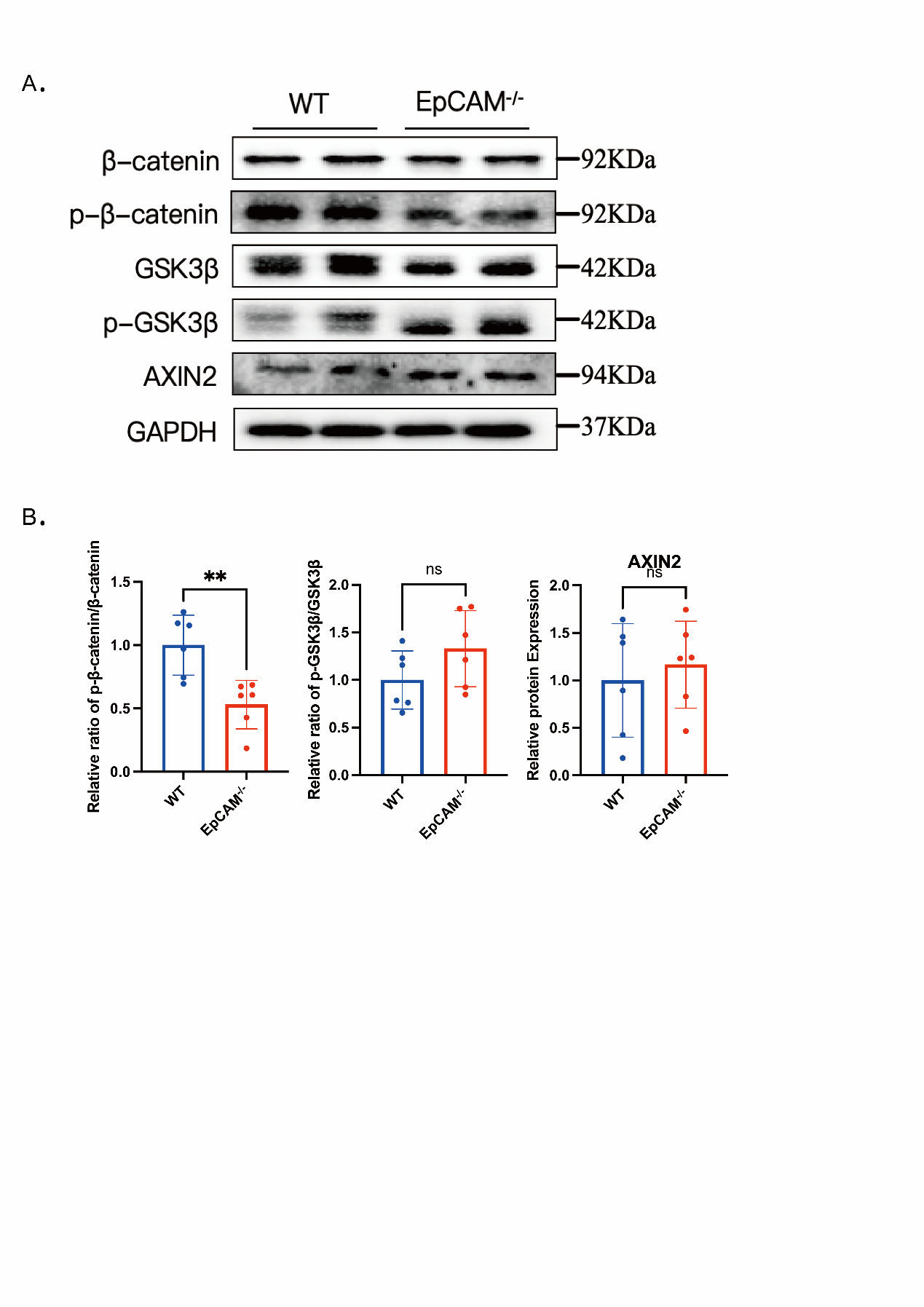


**Figure S30.** **The Deficiency of EpCAM Increased the Activity of Canonical WNT Pathway in the Small Intestines of E18.5 Embryos**

**A**. Western blot analysis of the levels of β-catenin, p-β-catenin, GSK3β, p-GSK3β and AXIN2 in the small intestines from WT and EpCAM^-/-^ groups of embryos at E18.5 stage. **B**. The quantification data of A. 6 mice in each group for 3 times independent experiments. **p<0.01.
