# Supplementary material for: Single-cell transcriptomics reveals EpCAM regulates the development and morphology of intestinal epithelium via controlling the EGFR pathway
Source: Genes Dis. 2026 Feb 9;13(5):102072. doi: 10.1016/j.gendis.2026.102072 (PMC13157056; doi:10.1016/j.gendis.2026.102072)
Supplement: Multimedia component 33 [file mmc33.docx]

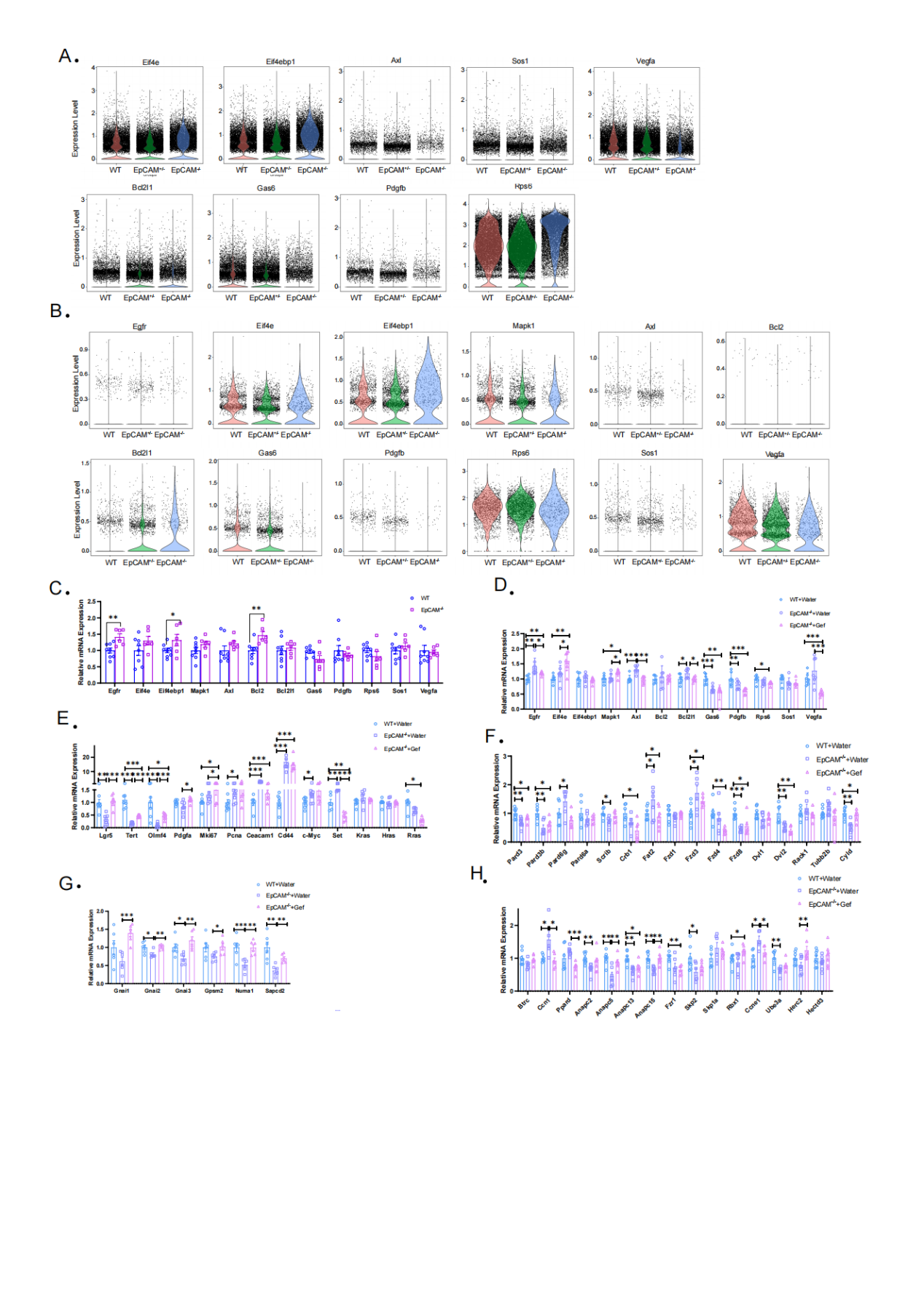


**Figure S31. The EpCAM Deficiency Affected the Homeostasis of the Intestinal Epithelium via the EGFR Signal Pathway**

**A**. Violin plots compared the expression levels of Eif4e, Eif4ebp1, Axl, Sos1, Vegfa, Bcl2l1, Gas6, Pdgfb and Rps6 in IECs from each genotype. **B**. Violin plots compared the mRNA levels of Egfr, Eif4e, Eif4ebp1, Mapk1, Axl, Bcl2, Bcl2l1, Gas6, Pdgfb, Rps6, Sos1 and Vegfa in IECs from Cluster 3. **C**. The qPCR results compared the relative mRNA expressional levels of Egfr, Eif4e, Eif4ebp1, Mapk1, Axl, Bcl2, Bcl2l1, Gas6, Pdgfb, Rps6, Sos1 and Vegfa in the intestines of each group. **D**. The relative mRNA expression of Egfr, Eif4e, Eif4ebp1, Mapk1, Axl, Bcl2, Bcl2l1, Gas6, Pdgfb, Rps6, Sos1 and Vegfa in the small intestines from WT + Water and EpCAM^-/-^ + Water and EpCAM^-/-^+Gef groups of embryos at E18.5 stage. **E**. The relative mRNA expression of Lgr5, Tert, Olmf4, Pdgfa, Mki67, Pcna, Ceacam1, Cd44, c-Myc, Set, Kras, Hras and Rras in the small intestines from WT + Water and EpCAM^-/-^ + Water and EpCAM^-/-^+Gef groups of embryos at E18.5 stage. **F**. The relative mRNA expression of Pard3, Pard3b, Pard6g, Pard6a, Scrib, Crb1, Fat2, Fzd1, Fzd3, Fzd4, Fzd8, Dvl1, Dvl2, Rack1, Tubb2b and Cyld in the small intestines from WT + Water and EpCAM^-/-^ + Water and EpCAM^-/-^+Gef groups of embryos at E18.5 stage. **G**. The relative mRNA expression of Gnai1, Gnai2, Gnai3, Gpsm2, Numa1 and Sapcd2 in the small intestines from WT + Water and EpCAM^-/-^ + Water and EpCAM^-/-^+Gef groups of embryos at E18.5 stage. **H**. The relative mRNA expression of Btrc, Ccn1, Ppard, Anapc2, Anapc5, Anapc13, Anapc15, Fzr1, Skp2, Skp1a, Rbx1, Ccne1, Ube3a, Herc2 and Hectd3 in the small intestines from WT + Water and EpCAM^-/-^ + Water and EpCAM^-/-^+Gef groups of embryos at E18.5 stage. ^*^p<0.05, ^**^p<0.01, ^***^p<0.001. Gef, Gefitinib.
