# Supplementary material for: Single-cell transcriptomics reveals EpCAM regulates the development and morphology of intestinal epithelium via controlling the EGFR pathway
Source: Genes Dis. 2026 Feb 9;13(5):102072. doi: 10.1016/j.gendis.2026.102072 (PMC13157056; doi:10.1016/j.gendis.2026.102072)
Supplement: Multimedia component 34 [file mmc34.docx]

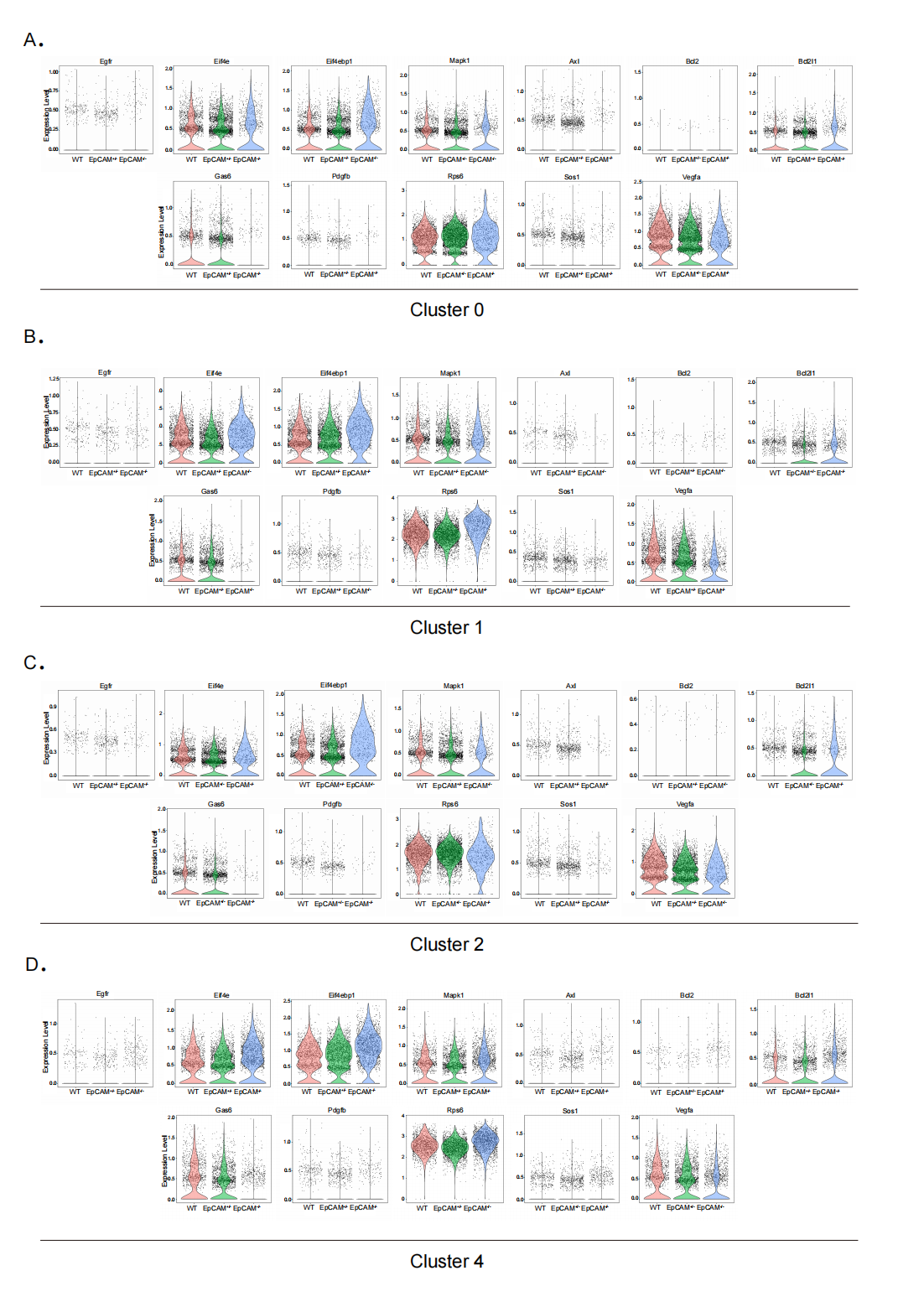


**Figure S32. Comparison of the expression of genes related to EGFR/MAPK signal pathway in the intestinal epithelial cells from WT, EpCAM^+/-^ and EpCAM^-/-^ mice**

**A**. Violin plots compared the mRNA levels of Egfr, Eif4e, Eif4ebp1, Mapk1, Axl, Bcl2, Bcl2l1, Gas6, Pdgfb, Rps6, Sos1 and Vegfa in the intestinal epithelial cells from Cluster 0 of WT, EpCAM^+/-^ and EpCAM^-/-^ mice. **B**. Violin plots compared the mRNA levels of Egfr, Eif4e, Eif4ebp1, Mapk1, Axl, Bcl2, Bcl2l1, Gas6, Pdgfb, Rps6, Sos1 and Vegfa in the intestinal epithelial cells from Cluster 1 of WT, EpCAM^+/-^ and EpCAM^-/-^ mice. **C**. Violin plots compared the mRNA levels of Egfr, Eif4e, Eif4ebp1, Mapk1, Axl, Bcl2, Bcl2l1, Gas6, Pdgfb, Rps6, Sos1 and Vegfa in the intestinal epithelial cells from Cluster 2 of WT, EpCAM^+/-^ and EpCAM^-/-^ mice. **D**. Violin plots compared the mRNA levels of Egfr, Eif4e, Eif4ebp1, Mapk1, Axl, Bcl2, Bcl2l1, Gas6, Pdgfb, Rps6, Sos1 and Vegfa in the intestinal epithelial cells from Cluster 4 of WT, EpCAM^+/-^ and EpCAM^-/-^ mice.
