# Supplementary material for: Single-cell transcriptomics reveals EpCAM regulates the development and morphology of intestinal epithelium via controlling the EGFR pathway
Source: Genes Dis. 2026 Feb 9;13(5):102072. doi: 10.1016/j.gendis.2026.102072 (PMC13157056; doi:10.1016/j.gendis.2026.102072)
Supplement: Multimedia component 35 [file mmc35.docx]

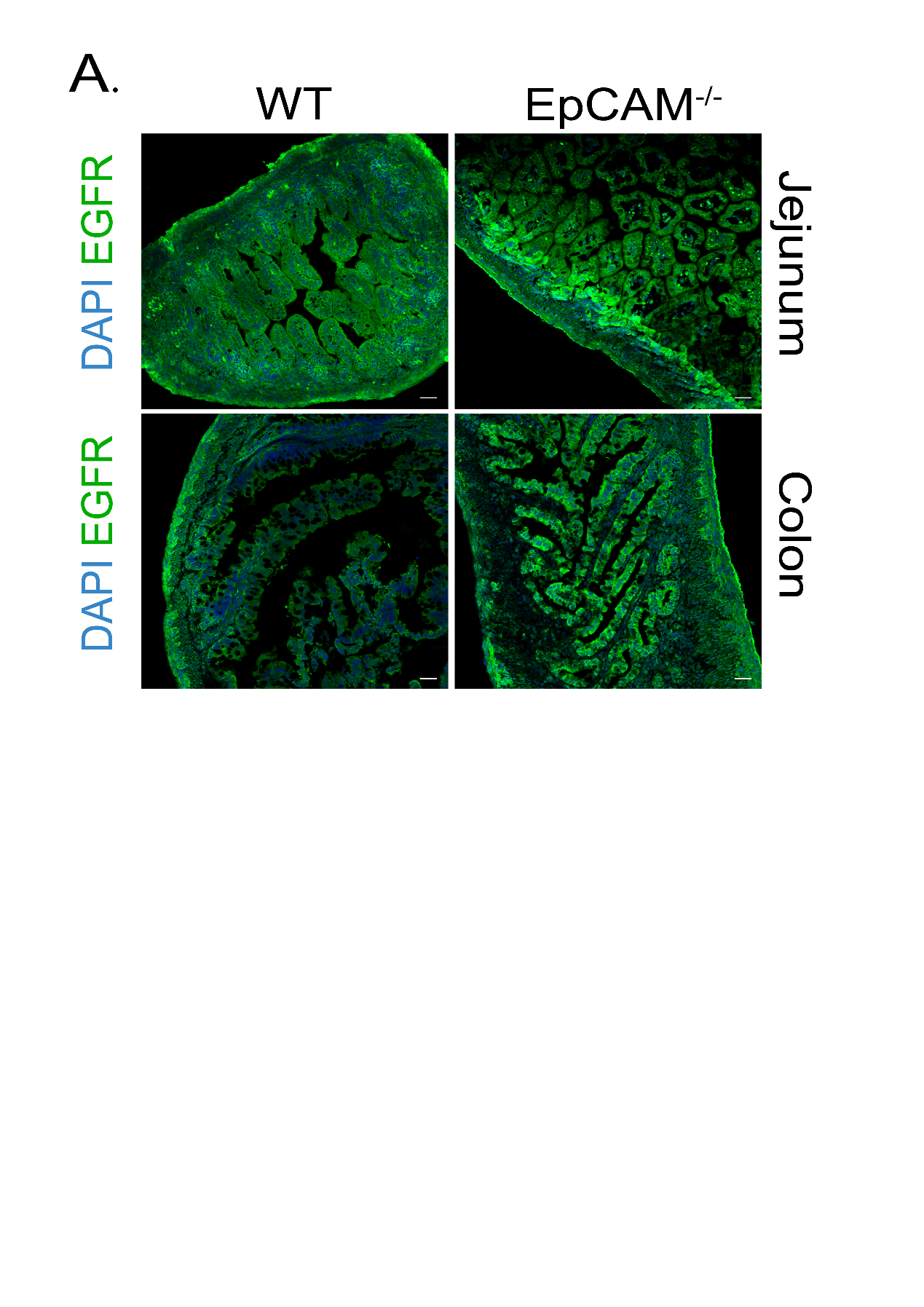


**Figure S33.** **The Deficiency of EpCAM Upregulated EGFR in both Small Intestines and Colons of Mice**

**A**. Frozen sections of jejunum and colon from WT and EpCAM^-/-^ mice at P2 were subjected to immunofluorescence staining with antibodies to EGFR. Nuclei were also stained with DAPI. Scale bar, 50μm.
