# Supplementary material for: Single-cell transcriptomics reveals EpCAM regulates the development and morphology of intestinal epithelium via controlling the EGFR pathway
Source: Genes Dis. 2026 Feb 9;13(5):102072. doi: 10.1016/j.gendis.2026.102072 (PMC13157056; doi:10.1016/j.gendis.2026.102072)
Supplement: Multimedia component 36 [file mmc36.docx]

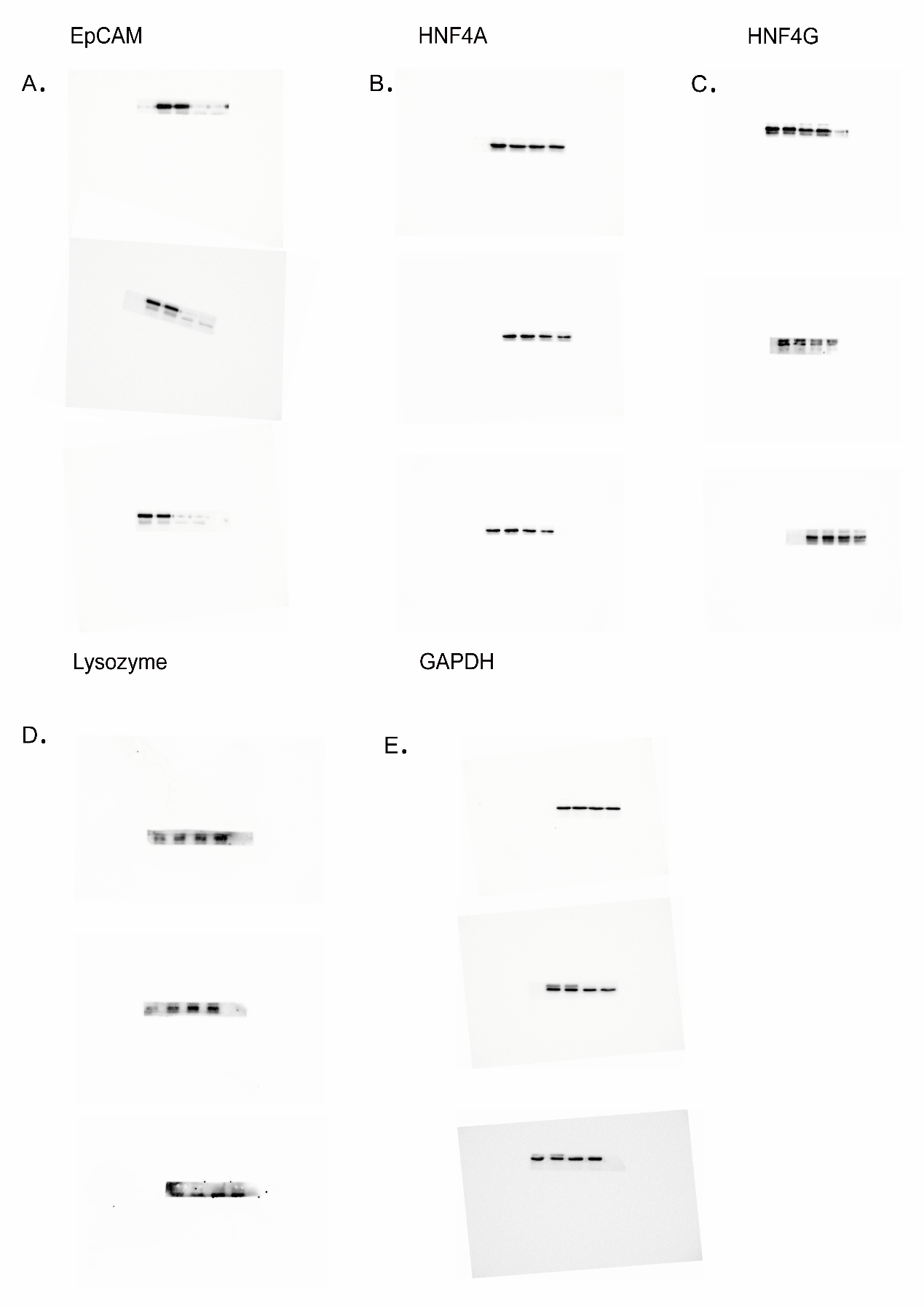


**Figure S34. Unedited blot and gel images of Figure 1D were shown**

**A-E**. Western blots in original figures of (A) EpCAM, (B) HNF4A, (C) HNF4G, (D) Lysozyme and (E) GAPDH respectively.
