# Supplementary material for: Single-cell transcriptomics reveals EpCAM regulates the development and morphology of intestinal epithelium via controlling the EGFR pathway
Source: Genes Dis. 2026 Feb 9;13(5):102072. doi: 10.1016/j.gendis.2026.102072 (PMC13157056; doi:10.1016/j.gendis.2026.102072)
Supplement: Multimedia component 37 [file mmc37.docx]

**
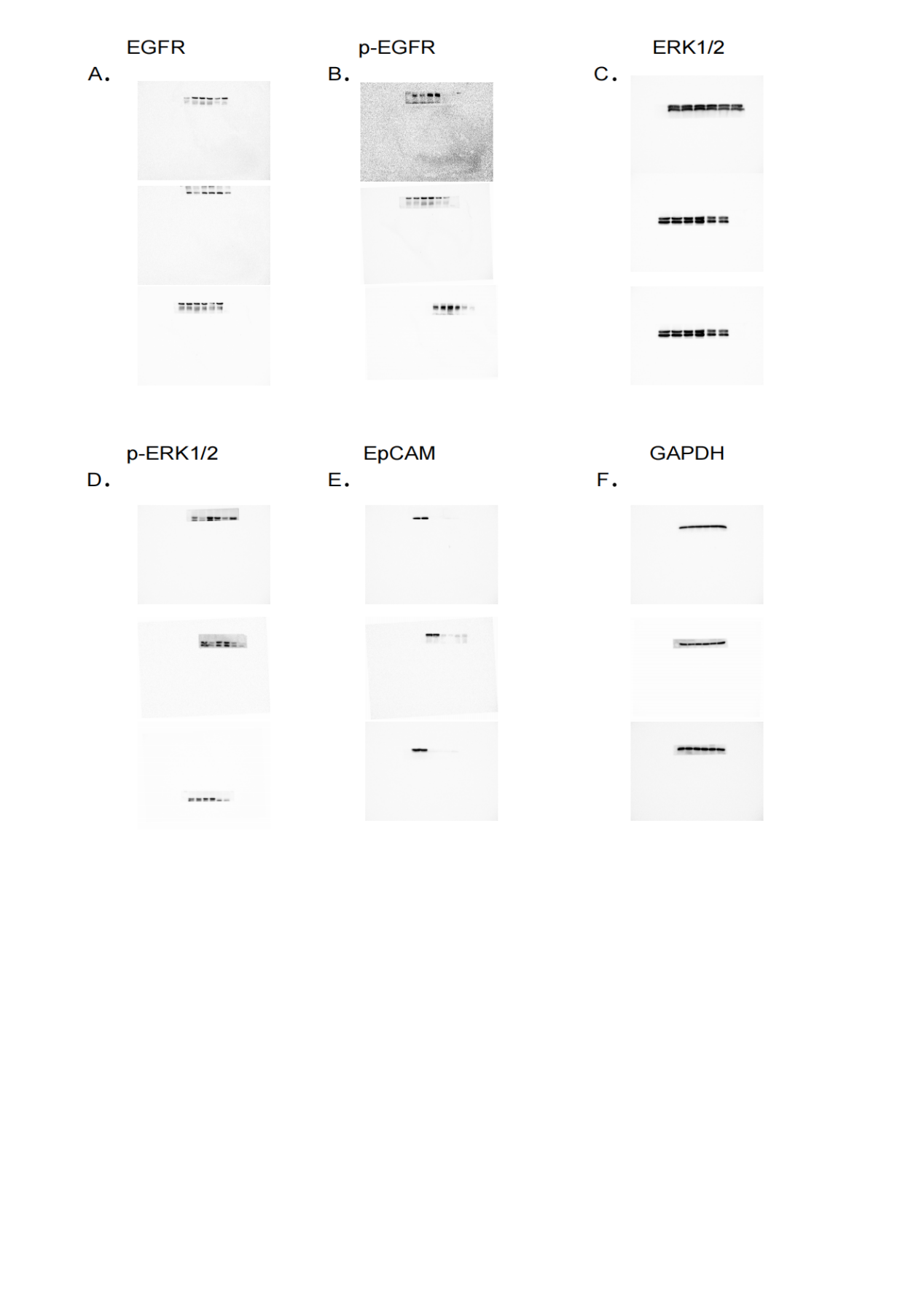
**

**Figure S35. Unedited blot and gel images of Figure 1M were shown**

**A-F**. Western blots in original figures of (A) EGFR, (B) p-EGFR, (C) ERK1/2, (D) p-ERK1/2, (E) EpCAM and (F) GAPDH respectively.
