# Supplementary material for: Single-cell transcriptomics reveals EpCAM regulates the development and morphology of intestinal epithelium via controlling the EGFR pathway
Source: Genes Dis. 2026 Feb 9;13(5):102072. doi: 10.1016/j.gendis.2026.102072 (PMC13157056; doi:10.1016/j.gendis.2026.102072)
Supplement: Multimedia component 38 [file mmc38.docx]

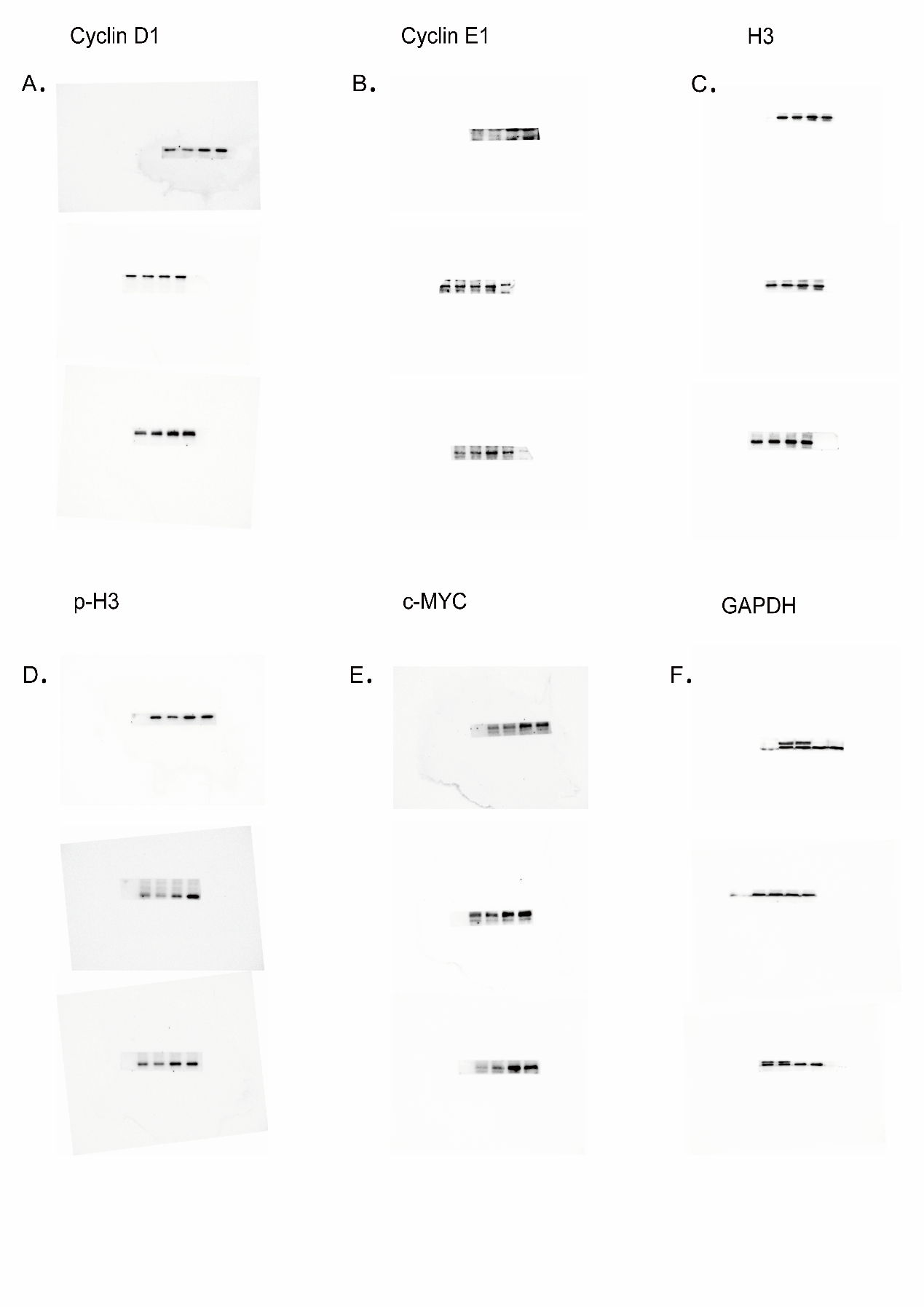


**Figure S36. Unedited blot and gel images of Figure S17 were shown**

**A-F**. Western blots in original figures of (A) Cyclin D1, (B) Cyclin E1, (C) H3, (D) p-H3 (E) c-MYC and (F) GAPDH respectively. H3, Histone 3; p-H3, p-Histone 3.
