# Supplementary material for: Single-cell transcriptomics reveals EpCAM regulates the development and morphology of intestinal epithelium via controlling the EGFR pathway
Source: Genes Dis. 2026 Feb 9;13(5):102072. doi: 10.1016/j.gendis.2026.102072 (PMC13157056; doi:10.1016/j.gendis.2026.102072)
Supplement: Multimedia component 39 [file mmc39.docx]

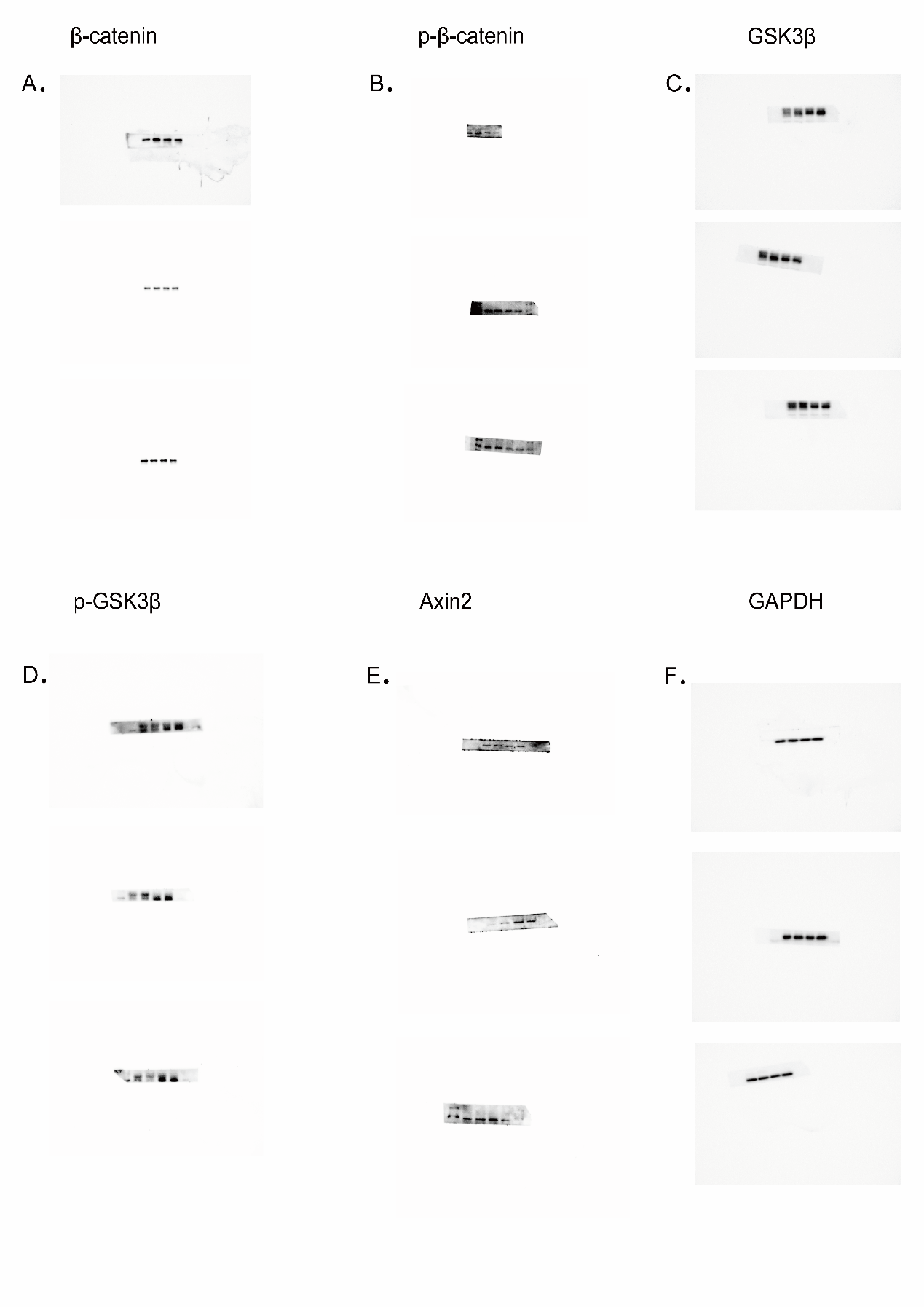


**Figure S37. The Unedited blot and gel images of Figure S30 were shown**

**A-F**. The Western blots in original figures of (A) β-catenin, (B) p-β-catenin, (C) GSK3β, (D) p-GSK3β, (E) AXIN2 and (F) GAPDH respectively.
